# TDP43 is a newly identified substrate for PS1, enhancing the expression of APP following cleavage

**DOI:** 10.1038/s41420-025-02340-z

**Published:** 2025-02-23

**Authors:** Hanlan Yin, Yuxiang Wang, Zhichao Ren, Zixuan Xiao, Yan Zhang, Yibo Wang, Zining Guo, Lu Chen, Xinlu Bao, Yingshuo Bei, Xueqi Fu, Linlin Zeng

**Affiliations:** Key Laboratory for Molecular Enzymology and Engineering of Ministry of Education, School of Life Science, Jilin University, Changchun, 130012 China

**Keywords:** Neuroscience, Proteases

## Abstract

Alzheimer’s disease (AD) has been comprehensively studied; however, most research has focused on Aβ plaque deposition and Tau protein phosphorylation. Emerging evidence suggests that TDP43 may be significantly involved AD and potentially worsening its pathology. To investigate the role of TDP43 in the pathological development of AD, we employed the STRING protein network interaction tool to identify potential relationships between TDP43 and other proteins, including PS1 and APP. Subsequent co-immunoprecipitation experiments were conducted, and the results indicated that TDP43 could interact with PS1. Further studies have shown that the interaction between the two would also lead to the loss of nuclear localization of TDP43. We also found that overexpression or knockdown of PS1 in both primary cells, HeLa and NSC34 cells indicated that TDP43 is likely to be a substrate of PS1. Subsequent use of the L685,458 and z-VAD, the PS1 mutant plasmids D257A and D385A, and bioinformatics approaches demonstrated that PS1 is dependent on γ-secretase and caspase activity to cleave TDP43, and that the cleavage site is at amino acid 315 of TDP43. Besides, our study demonstrated that the interaction of TDP43 with PS1 in primary cells, HeLa and NSC34 cells can promote APP expression, resulting in elevated Aβ levels. Finally, we investigated whether the interaction between TDP43 and PS1 affects the expression of other PS1 substrates, Notch and E-cadherin. Our results demonstrated that TDP43 cleaved by PS1 only promoted APP expression and had no effect on other PS1 substrates. In conclusion, these results suggest that TDP43 is a new substrate of PS1 and that TDP43 cleaved by PS1 promotes APP expression, which leads to increased Aβ content, which may explain why TDP43 promotes AD development. These insights enhance our understanding of TDP43’s role in AD development.

## Introduction

Alzheimer’s disease (AD) is the leading cause of dementia in the elderly worldwide, characterized by progressive and irreversible brain degeneration, which results in cognitive impairment and memory loss. Therefore, it has become an expensive and deadly disease, which has increased the burden of society in this century [1, 2]. Current estimation indicates that about 55 million people have AD worldwide, and this number is expected to increase to over 130 million in the next 30 years, with the highest incidence in developing countries [3]. Although comprehensive research has been conducted over the century since AD was first identified, its pathogenesis remains incompletely understood. The amyloid hypotheses posit that alterations in β-amyloid protein production and processing are key initiating factors for AD pathogenesis [4–6]. In this pathway, APP is cleaved by β-secretase and γ-secretase into Aβ_1-40_ and Aβ_1-42_ variants. It has been observed that Aβ_1-40_ is more abundant; however, Aβ_1-42_ has stronger hydrophobic properties, is more toxic, and is prone to aggregation [7, 8].

The term “γ-secretase” was coined in 1993 to describe the protease activity. After more than a decade of research, it was identified that γ-secretase was a complex of four subunits: Presenilin (PS1/PS2), Nicastrin, Aph-1, and Pen-2, assembled in a 1:1:1:1 stoichiometry [8–10]. In the cells, presenilins undergo autoproteolysis and predominantly exist as N-terminal fragments (PS-NTF, ~30 kDa) and C-terminal fragments (PS-CTF, ~20 kDa). These fragments are highly stable, and their levels are tightly regulated by protein degradation and complex formation. Mutations at Asp257/Asp385 in the 6^th^ and 7^th^ transmembrane regions of PS1 reduce about 60% of Aβ secretion [11]. Pathogenic mutations in presenilins that cause AD are primarily located in or near the transmembrane regions and invariably increase the Aβ42/Aβ40 ratios in cells and transgenic mice [12, 13]. However, γ-secretase/PS1 has many substrates other than APP, such as Notch and E-cadherin, whose normal processing is critical for organismal health [14, 15]. Therefore, inhibiting γ-secretase activity to reduce Aβ production can cause significant side effects, which can also cause death. For instance, the γ-secretase inhibitor, semagacestat, reduces plasma Aβ levels but promotes cognitive decline as well as increases the risk of skin cancer, immune system abnormalities, and gastrointestinal symptoms, which are all attributed to the inhibition of Notch cleavage [16]. Therefore, specific modulation rather than complete inhibition of γ-secretase activity on APP cleavage might be a more effective alternative for reducing Aβ levels and treating the disease. AD is a well-known age-related disease, and literature has identified that TDP-43 (TAR DNA-binding protein 43, TDP-43) proteinopathy is also age-related. TDP-43 is associated with many neurodegenerative diseases and several studies have indicated that the severity of neuropathology and cognitive decline varies in AD patients, potentially due to the co-occurrence of AD and TDP-43 proteinopathy. This data suggests that TDP-43 could be a novel therapeutic target for AD [17].

TDP-43 protein is encoded by the TARDBP gene and is a heterogeneous nuclear ribonucleoprotein located, with two RNA recognition motifs, nuclear localization and export signals, as well as a glycine-rich domain involved in protein-protein interactions. Furthermore, it has been associated with various cellular functions such as exon skipping, RNA stability, RNA transport, splicing, translation, and microRNA processing [18]. Similar to tau protein, TDP-43 proteinopathy is characterized by pathological hyperphosphorylation and abnormal TDP-43 aggregation in the neuronal cytoplasm and/or nuclear inclusions. Research has revealed that TDP-43 proteinopathy may cause cognitive dysfunction in AD patients [19]. Furthermore, amyloid-β plaque deposition and tau pathology in AD patients are affected by TDP-43 expression [20]. Moreover, TDP-43 overexpression in the APP/PS1 mouse model has been associated with increased pathological tau protein, suggesting a potential link between TDP-43 and tau pathology in AD [21]. In addition, TDP-43 interacts with mitochondrial proteins crucial for mitophagy, thereby altering mitochondrial morphology in TDP-43-expressing mice in the APP/PS1 background [22]. PS1 is involved in the cutting process of APP, thus affecting the production of Aβ via its γ-secretase activity. In recent years, it has been found that TDP43 also plays an important role in AD. For example, the consumption of TDP43 in the forebrain tissue of AD mice can aggravate neurodegeneration and is related to the increase of Aβ. TDP43 can also interact with APP or Aβ [23]. However, the interaction and mechanism between TDP43 and PS1 have been unclear. Overall, the literature suggests that TDP-43 plays a critical role in the pathological mechanisms of neurodegenerative diseases, specifically in AD. Further research is warranted to fully understand how TDP-43 influences amyloid-β plaque deposition, tau pathology, and mitochondrial function in AD patients. Research on complex interactions among TDP-43, APP, and PS1 in AD pathogenesis will provide valuable insights into potential therapeutic targets for AD.

## Results

### Interaction between TDP43 and PS1

**Fig. 1:**
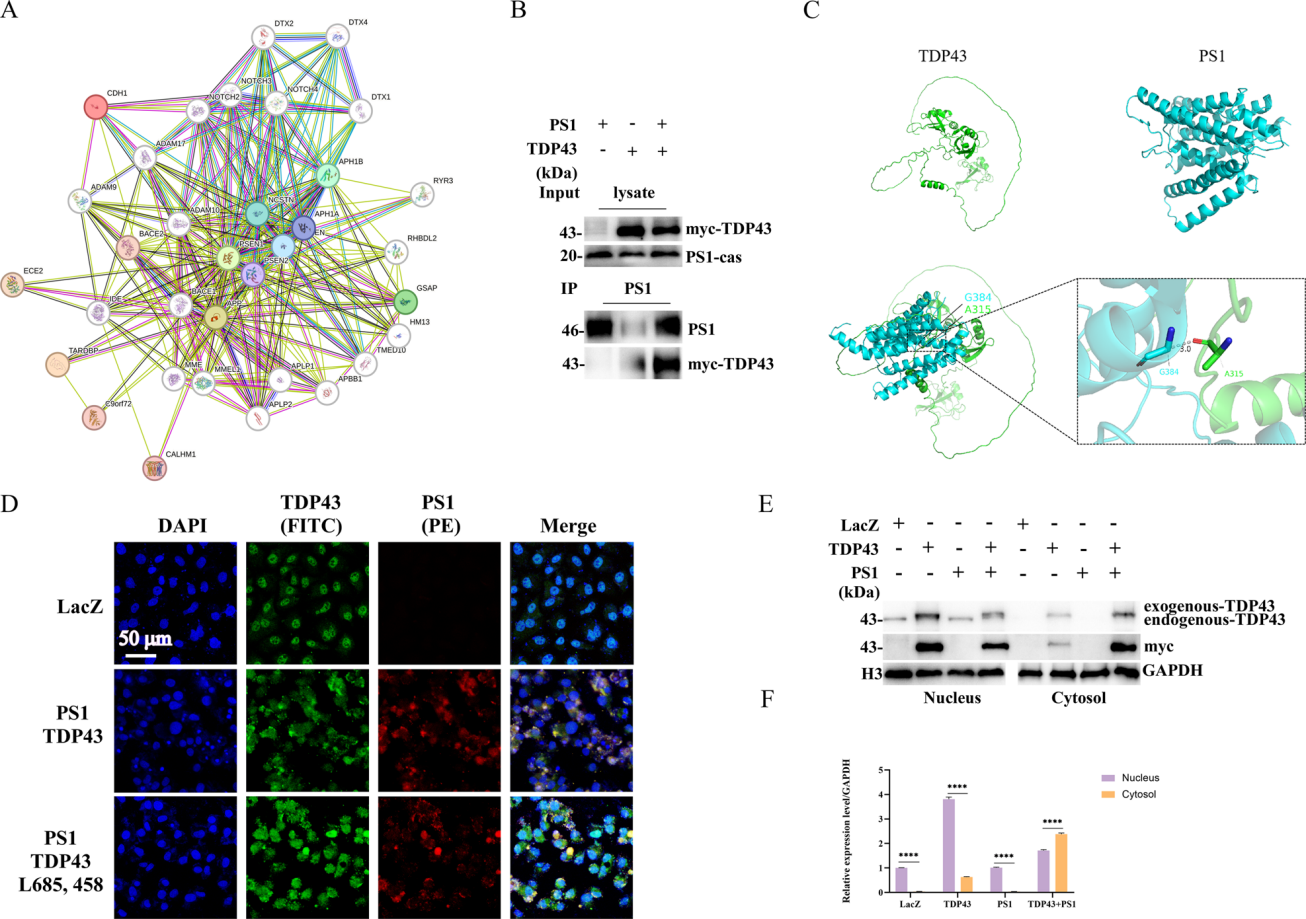
Interaction between TDP43 and PS1. **A** PPI network interaction between PS1, APP, and TDP43. The representative image of the protein-protein interaction (PPI) network of PS1, APP, and TDP43, indicating potential interactions among these proteins. **B** Co-immunoprecipitation (Co-IP) of TDP43 and PS1. HeLa cells were co-transfected with TDP43 and PS1 plasmids. After sample collection, a Co-IP assay was performed using a PS1 antibody, followed by western blotting with a c-Myc antibody to detect the interaction between TDP43 and PS1. **C** Bioinformatics prediction of the binding site between TDP43 and PS1. The z-DOCK online platform predicted the optimal binding position between TDP43 and PS1. The surface interactions of the two proteins were subsequently analyzed using Discovery Studio. **D** Immunofluorescence co-localization of TDP43 and PS1. HeLa cells were transfected with LacZ as a blank control (Group 1) and co-transfected with TDP43 and PS1 (Group 2), as well as TDP43 and PS1 with the addition of γ-secretase inhibitor L685,458 1 h before transfection (Group 3). After 24 h, immunofluorescence analysis was performed to detect the interaction between TDP43 and PS1. DAPI was used to stain the cell nuclei, TDP43 was labeled with FITC to emit green fluorescence, and PS1 was labeled with PE to emit red fluorescence. The merge image shows the integration of all three channels. **E** Impact of the interaction of TDP43 and PS1 on the nuclear localization of TDP43. TDP43 and PS1 were overexpressed in NSC34 cells, after which the nucleus and cytosol were isolated. Finally, the expression of TDP43 in the nucleus and cytosol was detected by WB. **F** Grayscale values of endogenous TDP43. The grayscale values in the graphs were calculated using Image J software analysis, and data are presented as mean ± SD. Two-way ANOVA with post hoc Bonferroni correction, compared with nucleus. (*n* = 3 independent experiments; *p < 0.05, **p < 0.01, ***p < 0.001, ****p < 0.0001, ns not significant).

STRING database (https://cn.string-db.org/) was employed to assess a potential relationship between TDP43, APP, and PS1, which indicated a potential functional co-expression between TDP43 and PS1 (Fig. 1A) [24]. Therefore, TDP43-Myc and PS1-Flag were co-transfected into HeLa cells to validate their interaction through Co-IP experiments, which confirmed their mutual interaction (Fig. 1B). Moreover, protein immunofluorescence co-localization revealed co-localization between TDP43 and PS1 (Fig. 1D). Furthermore, Z-DOCK was employed to assess the interaction between TDP43 and PS1. The results suggested a potential interaction between the two proteins (Fig. 1C). In addition, DS was utilized for surface analysis of the TDP43 and PS1 complex, which revealed several hydrogen bond formations, such as between PS1’s GLY384 and TDP43’s ALA315 (Supplementary Table 1).

It has been demonstrated that the loss of TDP43 nuclear localization results in the accumulation of cytoplasmic TDP43 aggregates, which further contributes to increased cellular stress, cell death, and the formation of neurodegenerative lesions [25]. To investigate whether the interaction between TDP43 and PS1 affects the nuclear localization of TDP43, we transfected both plasmids into NSC34 cells, extracted nuclear and cytoplasmic proteins, and observed that the interaction between TDP43 and PS1 contributed to the loss of nuclear localization of TDP43 through WB experiments (Fig. 1E and F).

### Construction of point mutant plasmids for TDP43

The data indicated that TDP43-A315T, TDP43-G348C and TDP43-N390D were primarily associated with ALS [26, 27]. Furthermore, this study predicted that the amino acid at position 315 is the site of interaction between TDP43 and PS1. Moreover, DS was used for performing virtual amino acid mutations, which indicated that the G348C and N390D mutations did not affect the affinity, while the A315T mutation resulted in a tighter binding between TDP43 and PS1 (Supplementary Fig. 2 and Supplementary Table 2, 3 and 6). In addition, we constructed plasmids carrying point mutations TDP43-A315T, TDP43-G348C, and TDP43-N390D. The sequencing results were aligned with the template sequences using SnapGene software, and the analysis indicated successful plasmid construction (Supplementary Fig. 1).

### TDP43 is a substrate of PS1

**Fig. 2:**
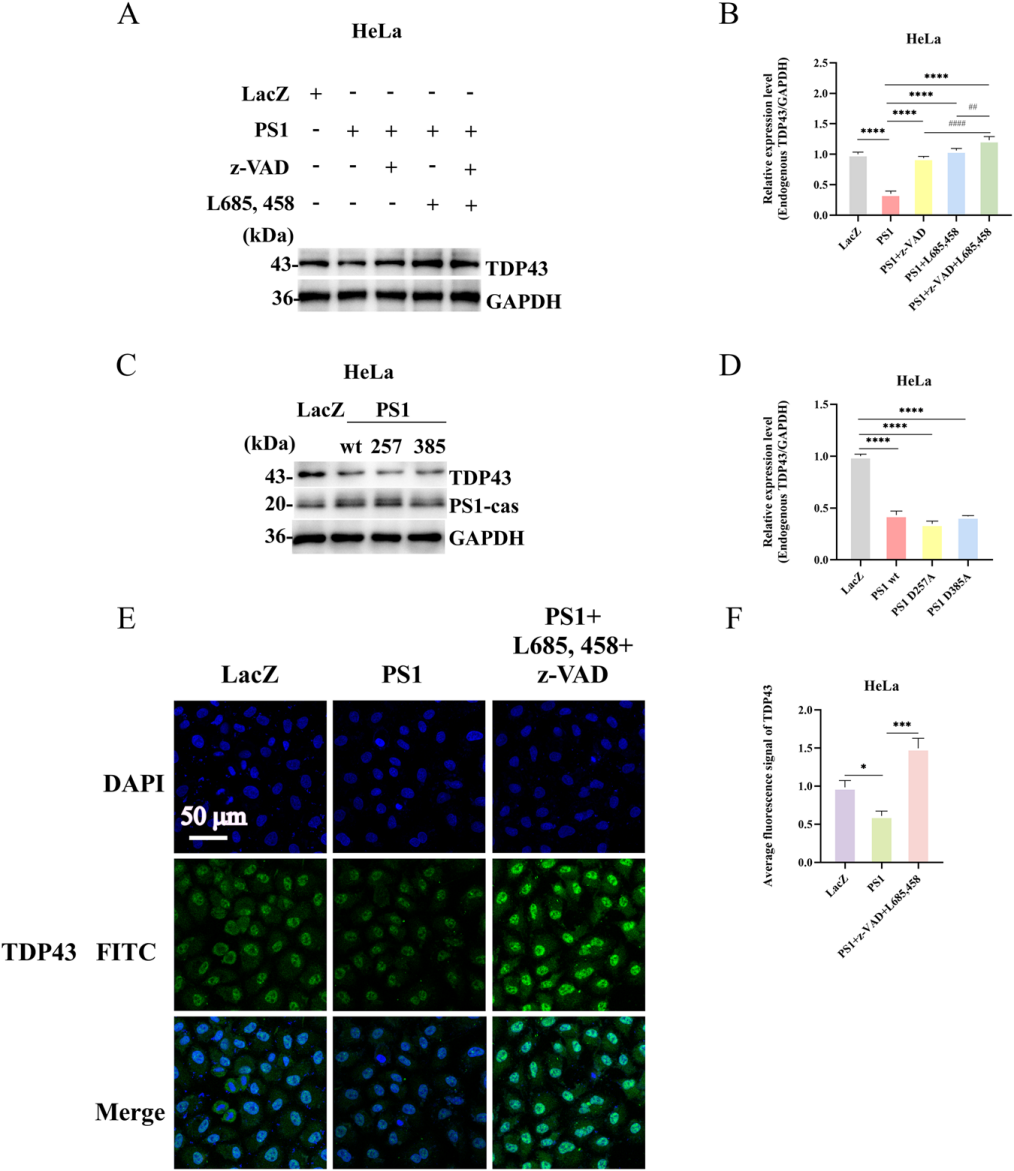
PS1 overexpression decreases the levels of TDP43 and γ-secretase inhibition promotes TDP43-43KD accumulation. **A** PS1 Cleavage of TDP43. HeLa cells were seeded in 35-mm dishes for 24 h. Then, one group was transfected with LacZ as a control, the second with 4 μg of PS1 plasmid, and the remaining three groups were pre-treated for 1 h with z-VAD, L685,458, and with their combination before transfection. Endogenous TDP43 levels were detected by western Blot analysis (Panel 1). The membranes in the top panels were reprobed with GAPDH antibodies to indicate the relative sample loading (bottom panels). **C** Effect of PS1 mutants on TDP43. HeLa cells were transfected with LacZ, PS1, PS1 D257A, and PS1 D385A for 24 h. Then, western blotting was performed to assess the levels of TDP43, and PS1-cas to verify that PS1 had been transfected into cells (panel 2). **E** Immunofluorescence detection of TDP43 levels after PS1 overexpression and inhibitor treatment. HeLa cells were transfected with LacZ as a blank control (Lane 1), PS1 alone (Lane 2), and pre-treated with z-VAD and L685,458 for 1 h before PS1 transfection (Lane 3). After 24 h, TDP43 levels were detected by immunofluorescence analysis. The TDP43 fluorescence intensity was decreased in the PS1 group, while, it increased in the inhibitor-treated group. DAPI was used to stain the nuclei, and TDP43 was labeled with FITC to emit green fluorescence. The merge image shows the integration of both channels. **B**–**F** Grayscale values of endogenous TDP43. The grayscale values in the graphs were calculated using Image J software analysis, and data are the mean ± SD of at least three independent experiments (*p < 0.05, **p < 0.01, ***p < 0.001, ****p < 0.0001, ns: not significant). The use of # indicates a comparison with the PS1+z-VAD + L685,458 group (# < 0.05, ## < 0.01, ### < 0.001, #### < 0.0001).

**Fig. 3:**
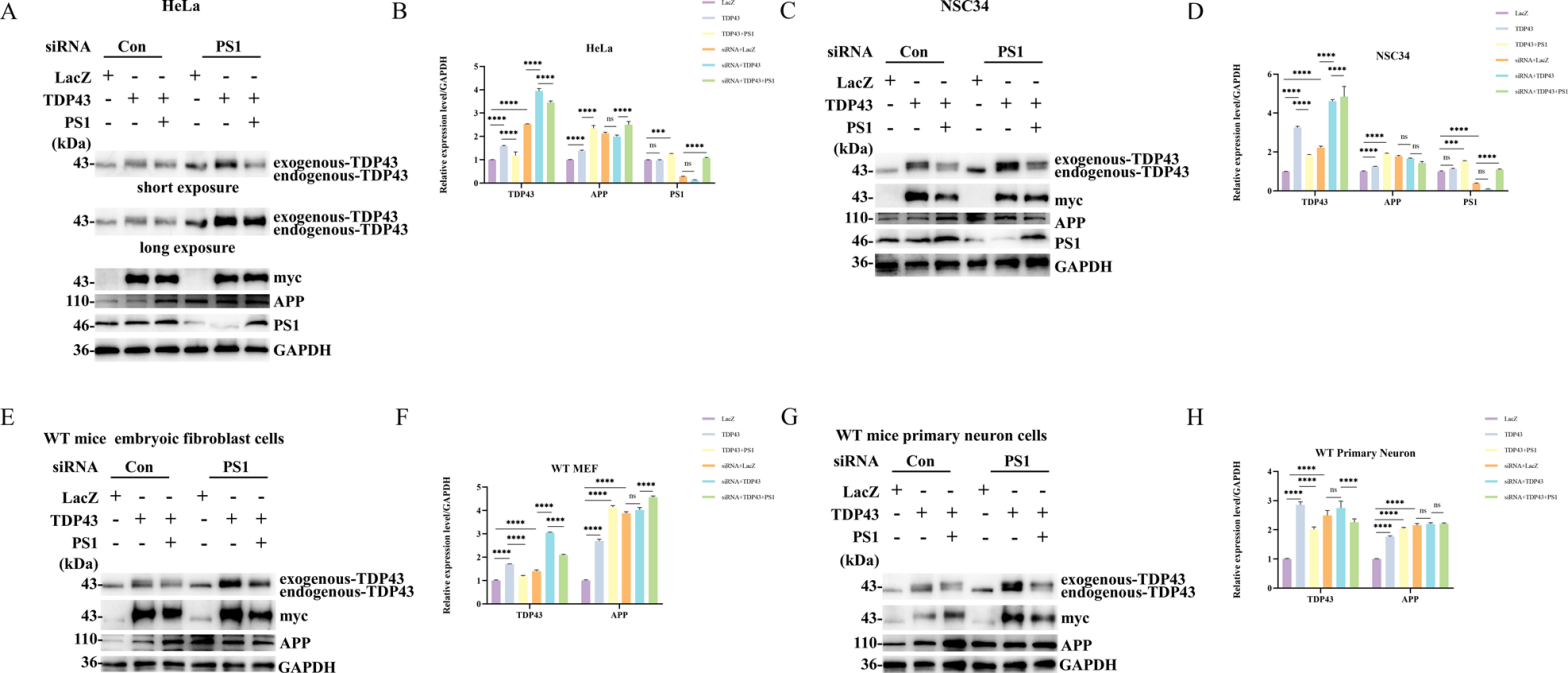
Effect of PS1 knockdown on APP and TDP43 expression. **A** Effect of whether to knock down PS1 in HeLa cells on APP and TDP43 expression. It examines the impact of TDP43 overexpression on APP expression in HeLa cells following transfection with NC and siRNA. Additionally, it investigates the alterations in TDP43 (panel 1 and 2) and APP (panel 4) expression in HeLa cells after backfilling with PS1. Relative protein level was calculated respectively by band intensity against GAPDH unless noted otherwise in this study. **C** Effect of whether to knock down PS1 in NSC34 cells on APP and TDP43 expression. The western blot of TDP43 (panel 1) and APP (panel 3) expression in NSC34 cells following siRNA knockdown of PS1 for 24 h, followed by transfection of TDP43 and PS1. **E** The effect of transfection of TDP43 and PS1 on TDP43 and APP expression in WT MEF cells following knockdown of PS1. Mice MEF cells were extracted from WT female mouse embryos at 11.5 days of gestation and subsequently transfected with siRNA for 24 h to knock down the expression of PS1 in WT MEF cells. Following this, the cells were transfected with TDP43 and PS1, the expression of TDP43 (panel 1) and APP (panel 3) was detected after 24 h, and the results were compared with those of the NC group. **G** siRNA-mediated knockdown of PS1 in primary neuron cells. NGF-induced differentiation of WT MEF cells into primary neuron cells. Then siRNA knockdown of PS1 in primary neuron cells was followed by transfection with TDP43 and PS1. The expression of TDP43 and APP was detected by WB. **B** and **D** Grayscale values of total TDP43, APP and PS1. **F** and **H** Grayscale values of total TDP43 and APP. The data are the mean ± SD of at least three independent experiments (*p < 0.05, **p < 0.01, ***p < 0.001, ****p < 0.0001, ns not significant).

To investigate the role of TDP43 in the predicted interaction between TDP43 and PS1, PS1 was overexpressed in HeLa cells. WB analysis indicated a decreased TDP43-43KD after PS1 overexpression (Fig. 2A, Panel 1). We then overexpressed TDP43 and PS1 in NSC34, HeLa, primary fibroblasts, and primary neurons. WB analysis revealed that the level of TDP43-43KD in the TDP43 and PS1 co-transfection group was lower than that in the TDP43 single transfection group. Subsequently, PS1 expression was silenced in NSC34, HeLa, primary fibroblasts, and primary neurons by siRNA transfection. WB results indicated an increase in TDP43-43KD content. Following PS1 replenishment, TDP43-43KD expression was diminished in these cells. Collectively, these findings substantiate that TDP43 is a novel PS1 substrate (Panel 1 of Fig. 3A, C, E and G).

### The cleavage of TDP43 is dependent on the γ-secretase and caspase activities of PS1

Given that PS1 exhibits both γ-secretase and caspase activities, we sought to elucidate the mechanism by which PS1 de-cleaves TDP43. TDP43-43 KD levels significantly increased after treatment with the broad-spectrum caspase inhibitor z-VAD and the γ-secretase inhibitor L685458, with the L685458 group exhibiting a more pronounced increase than the z-VAD group. Moreover, co-treatment with z-VAD and L685,458 significantly increased TDP43 even more (Fig. 2A, B). Furthermore, PS1 overexpressing as well as L685,458 and z-VAD treated HeLa cells were subjected to immunofluorescence experiments. The results revealed that the fluorescence intensity of TDP43 in the PS1 overexpression group was lower than in the LacZ group. However, in the L685,458 and z-VAD treated groups, the fluorescence intensity of TDP43 was increased (Fig. 2E, F). These findings suggest that PS1-mediated cleavage of TDP43 may not solely depend on the γ-secretase activity of PS1 but may also involve its caspase activity.

A substantial body of evidence from numerous studies has demonstrated that the active site of γ-secretase of PS1 is situated at positions 257 and 385 aspartic acid [28]. Consequently, PS1-WT, PS1-D257A and PS1-D385A were transfected into HeLa cells, which revealed reduced levels of TDP43 (Fig. 2C, D). This further indicates that PS1-mediated cleavage of TDP43 is not solely dependent on the γ-secretase activity of PS1.

### Alanine at position 315 of TDP43 is the site of cleavage by PS1

**Fig. 4:**
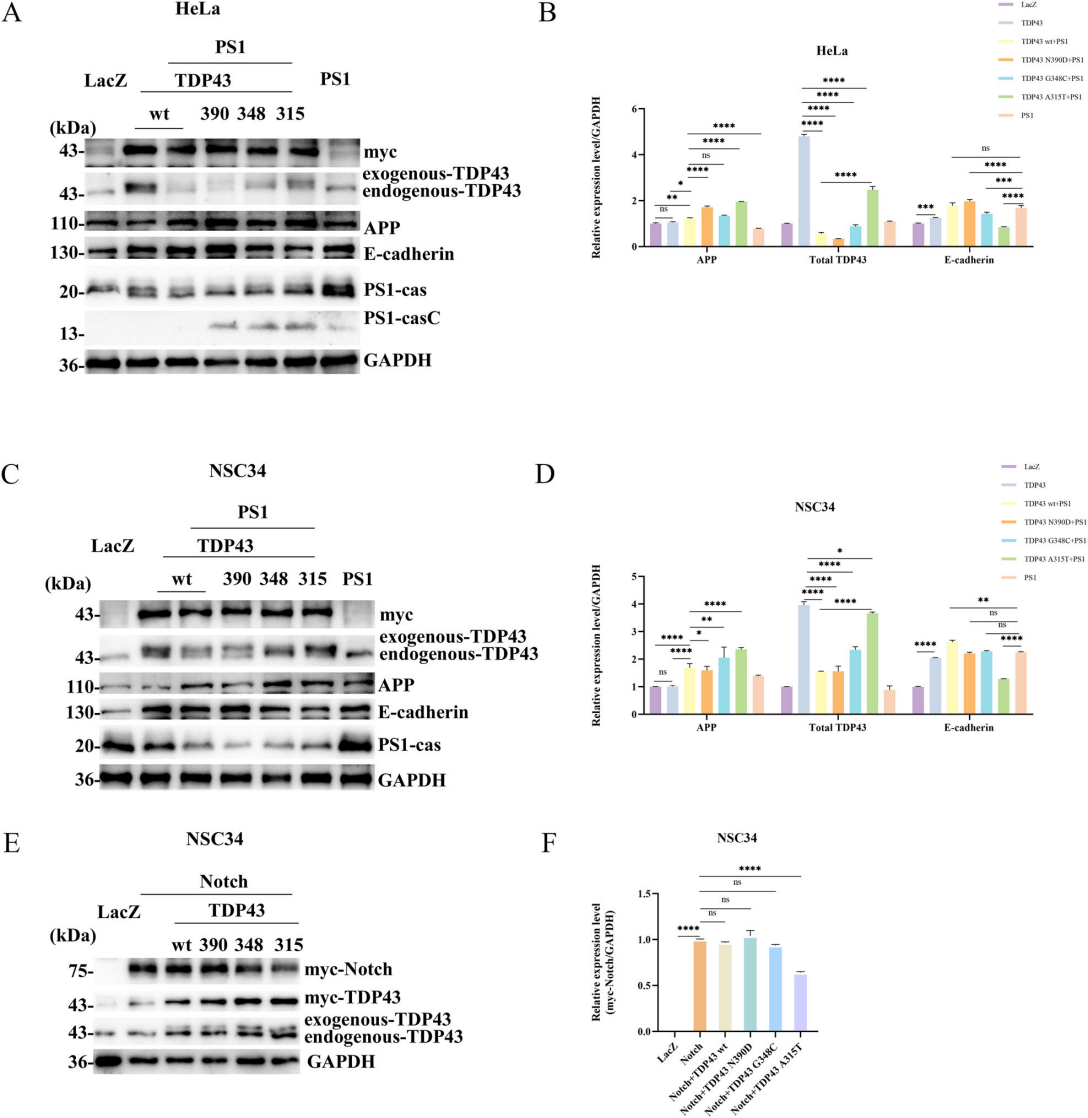
The effect of TDP43 and PS1 interaction on PS1/γ-secretase substrates APP, E-cadherin, and Notch. **A** and **C** TDP43 and PS1 interaction promotes APP expression without affecting E-cadherin levels. In the co-transfection groups, HeLa cells (**A**) and NSC34 cells (**C**) were seeded in 35-mm dishes and initially transfected with 4 μg of either TDP43-WT, -N390D, -G348C, or -A315T mutants. After 24 h, the cells were transfected with 4 μg of PS1. For the single transfection groups, HeLa cells were transfected with 4 μg of LacZ, TDP43, or PS1 after adhesion. After 24 h, cell samples were collected, and levels of APP (panel 3), E-cadherin (panel 4), and TDP43 (panel 5) were detected by western blotting. Myc was used to verify that TDP43 was transfected into cells. **E** TDP43 and PS1 interaction does not inhibit Notch cleavage; however, A315T mutant inhibits Notch cleavage. In the co-transfection groups, NSC34 cells were seeded in 35-mm dishes and simultaneously transfected with Notch. After 24 h, the cells were transfected with TDP43-WT, -N390D, -G348C, or -A315T. Samples were collected and myc-Notch levels were detected by western blotting. **B**, **D** and **F** Grayscale values of total TDP43, APP, and E-cadherin in HeLa Cells (**B**); Grayscale values of total TDP43, APP, and E-cadherin in NSC34 Cells (**D**); Grayscale values of myc-Notch in NSC34 Cells (**F**). The grayscale values are the mean ± SD of at least three independent experiments (*p < 0.05, **p < 0.01, ***p < 0.001, ****p < 0.0001, ns not significant).

In the preceding section, we demonstrated that PS1 is dependent on γ-secretase and caspase activities for the cleavage of TDP43. To investigate site at which TDP43 is cleaved, PS1-WT was co-transfected with TDP43-WT, TDP43-N390D, TDP43-G348C, and TDP43-A315T in HeLa and NSC34 cells. There was a significant increase in the levels of TDP43-43KD in the PS1-WT and TDP43-A315T co-transfection group compared to the PS1-WT and TDP43-WT co-transfected group (Panel 2 of Fig. 4A and C). This suggests that the cleavage site of TDP43 by PS1 is likely near the amino acid 315 of TDP43, consistent with the results of bioinformatics analysis.

### The interaction between TDP43 and PS1 increases the levels of APP, which in turn increases the Aβ levels

**Fig. 5:**
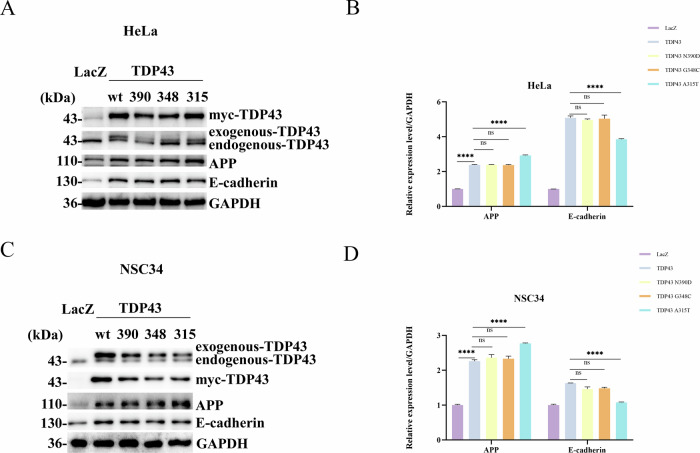
Effects of TDP43 and its three mutants on PS1/γ-secretase substrates. **A**–**C. TDP43 and its mutants increased APP levels in HeLa and NSC34 cells, while the A315T mutant affected E-cadherin cleavage**. HeLa cells (**A**) and NSC34 (**C**) were seeded in 35-mm dishes and transfected with 4 μg of either LacZ, TDP43-WT, -N390D, -G348C, or -A315T for 24 h before samples were collected, and levels of APP (Panel 3) and E-cadherin (Panel 4) were detected by western blotting. **B**, **D** The grayscale values for APP and E-cadherin in HeLa and NSC34. The grayscale values for APP and E-cadherin in HeLa (**B**) and NSC34 (**D**) are the mean ± SD of at least three independent experiments (*p < 0.05, **p < 0.01, ***p < 0.001, ****p < 0.0001, ns not significant).

**Fig. 6:**
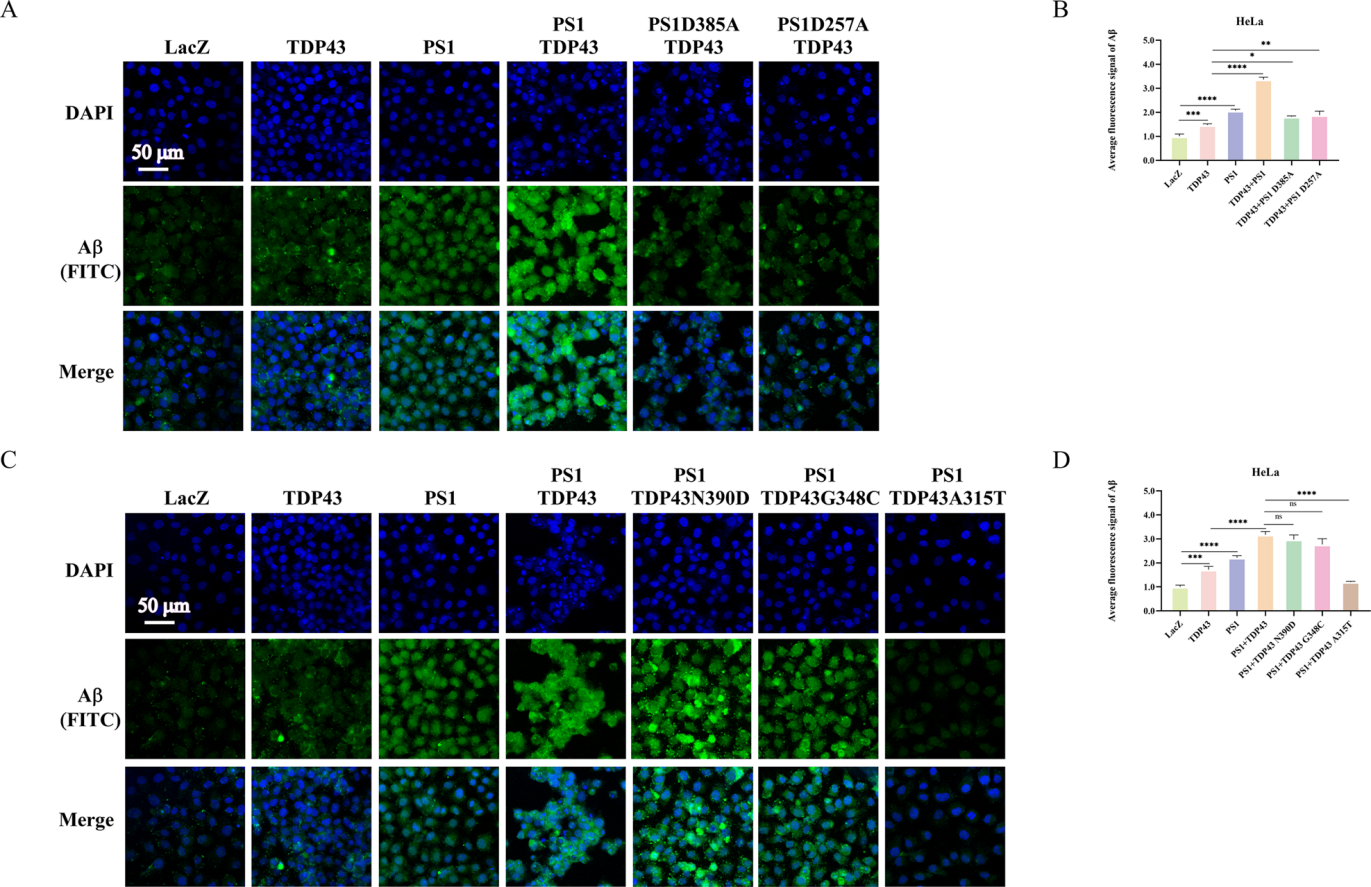
Interaction between TDP43 and PS1 increases Aβ levels. **A** Interaction of PS1 and its mutants with TDP43 increases Aβ levels. HeLa cells were transfected with LacZ, TDP43, and PS1, as well as co-transfected with TDP43 and PS1-WT or TDP43 and PS1-mutants (D257A and D385A). Aβ levels were detected using immunofluorescence, with DAPI marking cell nuclei and FITC marking Aβ (green fluorescence). The merge images show the integration of both channels. **C** Interaction of TDP43-WT, -N390D, and -G348C with PS1 promotes Aβ production, while TDP43-A315T mutant inhibits Aβ production. HeLa cells were transfected with LacZ, TDP43, PS1, or co-transfected with PS1 and TDP43-WT, -N390D, -G348C, or -A315T. Aβ levels were detected *via* immunofluorescence, with DAPI marking cell nuclei and FITC marking Aβ (green fluorescence). The merge images show the integration of both channels. **B**, **D** Statistical graphs illustrating Aβ fluorescence intensity. The fluorescence intensity of Aβ and the total area of all cells in the DAPI channel were calculated using the ImageJ software. The ratio of Aβ fluorescence intensity to the total area of the cells is the average fluorescence intensity of Aβ. Date are the mean ± SD of at least three independent experiments (*p < 0.05, **p < 0.01, ***p < 0.001, ****p < 0.0001, ns not significant).

It is well-known that APP is a crucial substrate of PS1, and Aβ is one of the major culprits in AD pathogenesis. To investigate whether the interaction between TDP43 and PS1 affects the levels of APP and Aβ by overexpressing TDP43 and PS1 in HELA, NSC34, primary fibroblasts and primary neurons cells. The results showed that the APP content was significantly increased in the group co-transfected with TDP43 and PS1 compared with the LACZ group (Panel 3 of Fig. 3A, C, E and G). Subsequently, TDP43-WT, TDP43-N390D, TDP43-G348C, and TDP43-A315T were transfected into HeLa and NSC34 cells. The WB analysis revealed a significant increase in the levels of APP in the transfected groups compared to the control group. The TDP43-A315T group indicated the highest levels of APP (Panel 3 of Fig. 5A and C). Furthermore, PS1-WT was co-transfected with TDP43-WT and the three-point mutation plasmids in HeLa and NSC34 cells. The WB revealed that the co-transfection of TDP43-WT, TDP43-N390D, TDP43-G348C, or TDP43-A315T with PS1-WT significantly increased APP levels compared to the groups only transfected with TDP43 or PS1. Moreover, the co-transfection of TDP43-A315T with PS1-WT showed the highest levels of APP among all groups (Panel 3 of Fig. 4A and B). These data forwarded a question of whether APP increase results from the inhibition of its cleavage or its increased expression due to the interaction between TDP43 and PS1. To answer this question, TDP43-WT was co-transfected with PS1-WT, PS1-D385A, or PS1-D257A in HeLa cells, and Aβ levels were detected through immunofluorescence. The results showed an increase in Aβ levels compared to the LacZ group (Fig. 6A). Then, PS1-WT was co-transfected with TDP43-WT, and the three-point mutation plasmids in HeLa cells, and Aβ levels were detected again *via* immunofluorescence, which revealed that the co-transfection groups had higher Aβ levels than the groups transfected with only TDP43-WT or PS1-WT. Moreover, all these groups showed higher Aβ levels compared to the LacZ group. However, the co-transfection of TDP43-A315T with PS1-WT decreased Aβ levels (Fig. 6C).

Subsequently, siRNA was used to silence PS1 expression in HELA, NSC34, WT mice embryoic fibroblast cells and WT mice primary neuron cells. This was followed by the overexpression of TDP43 and PS1. Immunofluorescence analysis demonstrated that the Aβ content in the group co-transfected with TDP43 and PS1 was higher than that in the LACZ and TDP43 groups (Supplementary Fig. 4A, C, E and G). Finally, TDP43 was transfected into AD primary neuron cells, and WB and immunofluorescence analyses demonstrated a significant increase in both APP and Aβ content (Supplementary Fig. 5C and E).

In summary, the elevated levels of Aβ are due to the cleavage of TDP43 following its interaction with PS1, which subsequently promotes APP expression. Consequently, APP is cleaved to form more Aβ, thereby contributing to the development of AD. Bioinformatics analysis indicates that the mutations TDP43-N390D, TDP43-G348C, PS1-D385 A, and PS1-D257A do not affect the interaction between TDP43 and PS1 (Supplementary Figs. 2 and 3, Supplementary Table 2, 3, 4 and 5). Therefore, N390D and G348C can still interact with PS1 to promote APP expression and cleavage, and PS1-D385A and PS1-D257A can cleave TDP43 in a caspase-dependent manner, leading to increased Aβ levels. In contrast, the A315T mutation results in a stronger binding affinity to PS1(Supplementary Fig. 2, Supplementary Table 6), inhibiting APP cleavage and thus reducing Aβ levels.

### The TDP43-PS1 complex only affects the levels of APP without influencing the γ-secretase cleavage of other substrates

In addition to APP, E-cadherin and NOTCH are also crucial substrates of PS1 [29]. The above data indicated that the interaction between TDP43 and PS1 promotes APP expression. However, whether this interaction also affects other PS1 substrates were evaluated by transfecting TDP43-WT and TDP43 point mutation plasmids (TDP43 A315T, TDP43 G348C, TDP43 N390D) into HeLa and NSC34 cells. The results of WB experiments revealed that compared to the control group, the TDP43-WT, TDP43-N390D, and TDP43-G348C transfection groups had increased levels of E-cadherin, which were reduced in the TDP43-A315T transfection group (Panel 4 of Fig. 5A and C). Moreover, PS1-WT was co-transfected with TDP43-WT and the three-point mutation plasmids in HeLa and NSC34 cells. The WB results showed that compared to the LacZ group, all co-transfection groups had increased E-cadherin levels, except for the co-transfection group of TDP43-A315T and PS1, where E-cadherin levels decreased (Panel 4 of Fig. 4A and C). Due to the difficulty in observing the cleavage of endogenous Notch, this study investigated whether TDP43 affects NOTCH cleavage by co-transfecting Notch with TDP43-WT and the three-point mutation plasmids in NSC34 cells. The data indicated that TDP43-WT, TDP43-N390D, and TDP43-G348C did not affect Notch cleavage; however, it was inhibited by TDP43-A315T (Panel 1 of Fig. 4E). The results demonstrate that the interaction between TDP43 and PS1 does not affect other substrates of PS1. However, A315T inhibits the γ-secretase activity of PS1, which increases APP levels and decreases Aβ levels, as well as inhibits NOTCH and E-cadherin cleavage.

## Discussion

In neurodegenerative diseases, the main pathological features include neuronal degeneration and the presence of numerous inclusions in surviving cells. The N-terminus of TDP-43 protein contains two tandem RNA recognition motifs, while the C-terminus is an intrinsically disordered region rich in glycine. Under pathological conditions, the C-terminus of TDP-43 undergoes fragmentation, and these fragments readily form inclusions, promoting the progression of neurodegenerative diseases such as AD. For instance, TDP-35 not only aggregates on its own but also exerts a seeding effect, causing nuclear TDP-43 to mislocalize and accumulate in the cytoplasm, accelerating the fibrillization of AD-related peptide Aβ_1-40_, thus promoting AD progression [30, 31]. In this study, we report that TDP-43 functions as a novel PS1 substrate, relying not only on PS1’s γ-secretase activity but also on its caspase activity. The fragments of TDP-43 generated by PS1 cleavage specifically regulate APP expression and Aβ levels, potentially contributing to AD development. Previous genetic analyses of some familial and sporadic amyotrophic lateral sclerosis (ALS) cases have identified over 50 missense mutations in the *TARDBP* gene [32]. In this study, we selected the TDP-43 mutants TDP-43-N390D, TDP-43-G348C, and TDP-43-A315T, which have been shown to cause ALS [30, 33]. However, their impact on AD remains unknown. Our preliminary findings indicate that TDP-43-N390D and TDP-43-G348C increase Aβ levels, but further experiments are needed to determine whether they promote Aβ fibrillation.

Several small molecule inhibitors targeting γ-secretase have been developed; however, they were not successful in clinical trials for AD treatment [34, 35]. The failure of these clinical trials may be attributed to the strong inhibition of γ-secretase activity by these inhibitors, which promotes fatal side effects, thus, preventing their clinical use. Therefore, the development of strategies that selectively regulate APP cleavage by γ-secretase/PS1 without affecting other substrates, especially Notch is urgently required. Studies have shown that downregulation of TMP21, a component of γ-secretase, increases Aβ levels without affecting Notch cleavage. However, TMP21 overexpression does not reduce Aβ levels and can cause additional side effects, such as growth retardation, neurological issues, and premature death [36]. Here, it was reported that TDP43 interacts with PS1 to upregulate APP levels, while also increasing the levels of Aβ. Therefore, TDP43 can modulate the substrate selectivity of γ-secretase/PS1, selectively regulating the production of both APP and Aβ. The important role of PS1 in the hydrolysis of APP is mainly dependent on its gamma-secretase activity. The overexpression of mutant PS1D385A and PS1D257A can block the hydrolysis of APP to produce Aβ, PS1D385A changes the γ-secretase activity of PS1, and PS1D257A has little effect on the activity of PS1 gamma-secretase [37]. Overexpression of TDP43 or cytoplasmic mislocalization caused by mutations are major factors involved in regulating the development of neurodegenerative diseases. TDP43 mutation can affect mitochondrial function and regulate autophagy pathway. The three mutant plasmids TDP43-A315T, TDP43-G348C and TDP43-N390D mentioned in this paper are located in the C-terminal region, which is rich in glycine. It is closely related to the nuclear transport of TDP43 and causes the aggregation of pathological fragments of TDP43 in the cytoplasm leading to neurotoxicity. However, it was also revealed that mutation of the 315^th^ amino acid of TDP43 inhibits the cleavage of APP, Notch, and E-cadherin, indicating that the activity of γ-secretase/PS1 is affected. According to the results of molecular docking experiments, the interaction site between TDP43 and PS1 is near the 315 site of TDP43. After PS1 is detached from TDP43, TDP43 or other substrates are cut. However, after the mutation of the 315 site of TDP43, the binding with PS1 is more firm, and the dissociation between PS1 and TDP43 is weakened. This weakens PS1’s cleavage of substrates, including TDP43, and thus blocks the hydrolysis of these substrates. Therefore, the co-expression of TDP43 and PS1 will increase APP content, and the co-transfer of TDP43G348C and N390D mutant plasmid with PS1 will not affect APP content, but the co-transfer of TDP43A315T with PS1 will increase APP content. Because PS1 dissociates from TDP43, TDP43 promotes the expression of APP protein after being cut by PS1, and TDP43A315T mutation enhances the binding of PS1-TDP43, blocking the cutting of TDP43, preventing the way that TDP43 promotes the expression of APP, and also blocking the cutting of APP by PS1. Therefore, after TDP43A315T and PS1 co-transfer, APP protein content increased and Aβ production decreased. However, TDP43G348C and N390D did not affect the interaction between TDP43 and PS1, and had little effect on the hydrolysis of APP compared with the wild-type plasmid TDP43. This also suggests that for using TDP43 as a therapeutic target for AD, its normal function should not be interfered with. This can be achieved by modulating TDP43 levels or inhibiting the interaction between TDP43 and PS1 to regulate Aβ levels.

In summary, this study revealed that TDP43 can interact with PS1, and the interaction site is alanine at position 315 of TDP43, which is cleaved by PS1’s γ-secretase and caspase activities. Furthermore, TDP43 does not affect the expression of Notch and E-cadherin; however, it promotes the expression of APP, thereby increasing the Aβ content. However, while the cleavage of TDP43 by PS1 leads to elevated Aβ levels, it remains unclear whether this promotes Aβ aggregation or if TDP43 itself aggregates after being cleaved. Therefore, we aim to conduct further experiments in the future to explore these aspects and elucidate the detailed mechanisms by which TDP43 contributes to the pathological development of AD.

## Materials and methods

L685,458 was purchased from MedChem Express (NJ, United States) and Z-VAD-FMK from Selleck (TX, United States). DAPI solution (1 mg/mL) and lipofectamine gene transfection reagent were acquired from Meilunbio (Dalian, China). Opti-MEM was bought from Gibco, Thermo Fisher Scientific (MA, United States). The serine protease inhibitor PMSF (100 mM) was provided by Solarbio (Beijing, China). The complete protease inhibitor cocktail tablets and protein A/G Magnetic Beads were acquired from Bimake (TX, United States). Anti-H3 antibody was synthesized by ABclonal (Wuhan, China). ExKine^TM^ Nuclear Protein Extraction Kit was synthesized by Abbkine (Wuhan, China). Mouse β-NGF, Poly-D-Lysine, B-27 supplement (50×) and Neurobasal^TM^ medium were bought from Thermo Fisher Scientific (MA, United States). Anti-APP antibody was synthesized by Wuhan Baiyixin Biotechnology (Wuhan, China). Anti-GAPDH Polyclonal Antibody, Goat Anti-Mouse IgG/FITC, Goat Anti-Rabbit IgG/PE and 10% Normal Goat Serum were from Bioss (Beijing, China). Presenilin 1 (D39D1) was provided by Cell Signaling Technology (MA, United States). TARDBP (H-8) was obtained from Santa Cruz Biotechnology (TX, United States). c-MYC tag Monoclonal antibody and Goat anti-rabbit IgG (H + L)/HRP were from Proteintech Group (Wuhan, China). Goat anti-mouse IgG (H + L)/HRP was bought from Biodragon. NSC-34 cells were obtained from the BeNa Culture Collection (Suzhou, China). HeLa cells, LacZ, PS1 WT, PS1 D385A, and PS1 D257A plasmids were stored in the Fisher Laboratory, College of Life Sciences, Jilin University. The Human TARDBP Gene ORF cDNA clone expression plasmid and C-Myc tag pCMV 3-TARDBP-Myc plasmid were acquired from Sino Biological Inc (Beijing, China).

### Protein-protein docking and virtual amino acid saturation mutagenesis

For protein-protein docking, the ZDOCK server (https://zdock.wenglab.org) was employed using TDP-43 as a receptor and PS1 as a ligand. The structure of TDP-43 was predicted by AlphaFold [38, 39], while that of PS1 [Protein Data Bank (PDB) ID: 6IYC] was obtained from the PDB database (https://www.rcsb.org/) [40]. Discovery Studio 2018(DS) was employed to analyze the docking results of surface interactions, while for visualization, PyMol software was utilized. Virtual amino acid mutagenesis was performed using DS. The CHARMM force field was applied to the proteins by opening the docking results in DS. Then, the input was set to the TDP-43 complex, with the ligand chain set to TDP-43 and the mutation site set to single mutations. Furthermore, the TDP-43 amino acid positions 390, 348, and 315 were individually mutated to each of the 24 other standard amino acids using default settings. This process was repeated for PS1 by setting the ligand chain to PS1 and performing saturation mutagenesis at amino acid positions 385 and 257. Based on the calculated mutation energies, the alterations in binding affinity between the receptor and ligand were assessed.

### Construction of TDP-43 point mutation plasmids

The TDP-43 coding sequence was downloaded from NCBI. Furthermore, Primer Premier software (PREMIER Biosoft, Version 5, USA) was employed to design the point mutation primers for TDP-43 (Table 1). Site-directed mutagenesis PCR amplification was performed on the pCMV3-TARDBP-Myc plasmid. Subsequently, DpnI enzyme were added to the PCR product to digest the methylated template. The resulting product only comprised the amplified PCR fragments, which were then transformed into DH5α competent cells. Plasmid DNA was amplified and extracted from the DH5α *E. coli* and sent to Tianjin Jinweizhi Biotechnology for sequencing. SnapGene software was then utilized to compare the sequencing results with the TDP43 template.

**Table 1:** PCR primers for TDP43 point mutation.

PCR Primers		
Gene	Fw(5′-3′)	Rw(5′-3′)
TDP43-A315T	GATGAACTTTGGTACGTTCAGCATTAATC	GATTAATGCTGAACGTACCAAAGTTCATC
TDP43-G348C	GCCAGCAGAACCAGTCATGCCCATC	GGTTATTACCCGATGGGCATGACTGG
TDP43-N390D	GGTTGGGGATCAGCATCCGATGCAGGGTCGGGC	CACTGCCCGACCCTGCATCGGATGCTGATCCCC

### Cell Culture and Transfection

The NSC-34 mouse motor neuron cells and HeLa cervical cancer cells were used in this study and cultured in high glucose DMEM supplemented with 10% fetal bovine serum and 1% penicillin-streptomycin at 37 °C in a 5% CO_2_ incubator. For transfection, 80% confluent cells seeded in 35 mm dishes were used. Plasmid DNA (in micrograms) was mixed with Transfection Reagent at a ratio of 1:2. For cell co-transfecting TDP43 and PS1, the transfection ratio of the two plasmids is 1:1.

### Extraction of WT and AD model mice embryoic fibroblast (MEF) cells

The AD model mice were SPF-grade APP/PS1 transgenic mice, all of which were purchased from Shanghai Model Organisms Center, Inc. Wild-type (WT) mice were also SPF-grade and purchased from Beijing SPF Biotechnology Co., Ltd. All experimental animals were housed and cared for in accordance with the requirements of the Welfare Ethics Committee, College of Life Sciences, Jilin University. Ethics review acceptance number: YNPZSY2023005. IACUC Issue number: (2023) YNPZSY (0201). The experiments were conducted in compliance with the regulations of the People’s Republic of China on the management of laboratory animals. All mouse strains used in this study were C57BL/6 J. We used CO_2_ euthanasia to euthanize mice to reduce pain and discomfort. To obtain pregnant AD mice, AD females were bred with males. Similarly, to obtain pregnant WT mice, WT females were bred with males. Subsequently, we randomly euthanized AD females and WT females that were 11.5 days pregnant and immersed them in 75% ethanol for 15 min. The uterus was then removed and washed three to five times with ice-cold PBS containing penicillin-streptomycin antibiotic in order to remove residual blood, stripped of its membranes, and the fetal mice were immersed individually in DMEM complete medium to await processing. One fetal rat was removed from the 75% alcohol solution and placed in a dish containing PBS. The head, limbs, and viscera were removed and the remaining skin tissue was clipped and washed two or three times with PBS. Subsequently, 3 mL of 0.25% pancreatic enzyme was added, followed by digestion at 37 °C for 20 min. During this process, the dish was shaken every 5 min, after which complete medium was added to terminate digestion. The supernatant was removed by centrifugation at 1000 × g for 5 min, after which the cells were resuspended in DMEM complete medium containing 15% FBS. Subsequently, the cells were transferred to 100 mm dishes and incubated in a 37 °C CO_2_ incubator for 1 week, with medium changes performed every 3 days.

### NGF induces MEF cells to differentiate into primary neuron cell

MEF cells were seeded into six-well plates that had been coated with poly-D-Lysine (25 μg/mL in PBS) and cultured in DMEM complete medium for 24 h. Following adherence to the substrate, the medium was replaced with Neurobasal neuron-specific medium (1% penicillin-streptomycin, 1% L-glutamine, and 2% B27). Subsequently, NGF (final concentration of 50 ng/mL) was introduced to induce differentiation for 24 h. The medium composition consisted of 1% penicillin-streptomycin, 1% L-glutamine, 2% B27, and 96% Neurobasal, with NGF (final concentration of 50 ng/mL) added to induce differentiation for 24 h. Whether MEF cells was induced in primary neuron cells was ultimately determined through the implementation of immunofluorescence staining for MAP2 and β-tubulin.

### The silencing of PS1 by siRNA

The siRNA sequence of PS1 is shown in Table 2. Transfection of siRNA resulted in the silencing of PS1 expression in HELA, NSC-34, primary fibroblasts, and sensory neurons. Firstly, the cells were inoculated in 6-well plates, and the siRNAs were transfected when the cell density reached 70% and 100 pmol of siRNA was diluted with 5 μL of KEL-R Transfection Reagent, which was diluted using 100 μL of OPTI-MEM, respectively. The solutions were incubated at room temperature for 5 min, and then the dilutions of siRNA and KEL-R Transfection Reagent were mixed, blended gently, and left to stand at room temperature for 20 min. Finally, the mixture was added to the cells and gently shaken.

**Table 2:** The siRNA sequence of PS1.

PS1 siRNA	
Species	5’-3'
human	GGUCCACUUCGUAUGCUGGTT
	CCAGCAUACGAAGUGGACCTT
mouse	UGGAUGUUUCUUCUUUGAUUAUCTT
	GAUAAUCAAAGAAGAAACAUCCATT

### Extraction of nuclear proteins

A total of 2 × 10^7^ NSC34 cells were collected, washed with 1 mL ice-cold PB, and centrifuged at 500 × g for 5 min. The supernatant was discarded, and the cells were washed with ice-cold PBS and centrifuged at 500 × g for 5 min, after which the supernatant was removed, CESA was added, and vortexing and mixing were conducted to ensure a complete cell suspension. The samples were then placed on ice and incubated for 15 min, which resulted in cellular swelling. CESB was then added, vortexed, and mixed, and the sample was again incubated on ice for 2 min. The mixture was centrifuged at 4 °C and 16,000 × g for 5 min. The supernatant was transferred to a 5KD ultrafiltration tube for ultrafiltration concentration, thus obtaining the cytoplasmic extract. Ice NES was added to the precipitate, vortexed, and mixed. Subsequently, the precipitate was allowed to stand on ice for 30 min, during which time it was vortexed every 10 min for 15 s. Thereafter, centrifugation was performed at 4 °C and 16,000 × g for 5 min, after which the supernatant was transferred to a new EP tube, which contained the nuclear protein extract.

### Western Blotting (WB)

The cells were lysed using PARP protein lysis buffer supplemented with 100x protease inhibitors cocktail and 1000x PMSF, followed by sonication at 4 °C for 20 s. The proteins in the samples were quantified using the BCA method. The sample loading volume was calculated and 20 μg of protein was subjected to SDS-PAGE electrophoresis. Primary antibodies: anti-H3 (1:1000), anti-APP (1:1000), PS1 (1:1000), Myc (1:5000), TDP43 (1:1000), GAPDH (1:3000), and E-cadherin (1:1000). Secondary antibodies: goat anti-rabbit IgG (H + L)/HRP (1:2000) for APP, PS1, and GAPDH, while goat anti-mouse IgG (H + L)/HRP (1:2000) for TDP43, Myc, and E-cadherin.

### Co-immunoprecipitation (Co-IP) analysis

TDP43-MYC, PS1-FLAG, PS1-FLAG, and TDP43-MYC were individually transfected into HeLa cells for 24 h. Cell lysates were prepared using the IP Lysis Buffer containing protease inhibitors (1% NP-40, 150 mM NaCl, 50 mM pH 7.4 Tris, and 10% glycerol dissolved in ddH2O). The samples were incubated overnight at 4 °C with PS1 antibody(2 μg) in the IP Binding Buffer (150 mM NaCl, 50 mM pH 7.0 Tris, and 10% glycerol dissolved in water) containing 1 μL of 1 mM DTT. The next day, each IP sample were mixed with 25 μL Protein A/G Magnetic beads and incubated for 2 h at 4°C with constant agitation. The beads were then washed with IP Washing Buffer (1:1 mixture of IP Lysis Buffer and IP Binding Buffer). Then, each sample was mixed with 20 μL of 1x Loading Buffer containing protease inhibitors and heated at 100 °C for 10 min, centrifuged for 10 min at 16,000 g, followed by the collection of supernatants. The samples were subjected to WB using anti-PS1 and Myc antibodies.

### Protein immunofluorescence co-localization

The slides were placed in a 24-well plate, cells were seeded and transfected with TDP43 plasmid. After adherence, the cells were transfected with PS1 plasmid for 24 h, then, washed with ice-cold PBS, fixed with 4% paraformaldehyde for 30 min. Then, the cells were permeabilized with 0.3% Triton X-100 for 5 min, blocked with 5% lamb serum and 0.1% Tween20 in PBS for 45 min, and incubated overnight at 4 °C with anti-PS1 and TDP43 antibodies (1:300). The next day, cells were washed with PBST and treated with fluorescent secondary antibodies for 2 h in the dark, followed by four times washing with PBST. Then, the slides observed under a confocal microscope (LSM710, Zeiss, Germany).

### Statistical analysis

Statistical data analysis was performed with GraphPad Prism version 7.0 (GraphPad Software) on at least three biological replicates for each experiment (see figure legends for details). Data satisfy homogeneity of variance test and are presented as the mean standard deviation (SD). The data were analysed for normal distribution. Differences between multiple groups were checked using one-way ANOVA and two-way ANOVA with post hoc Bonferroni correction. Differences between two groups were analysed by a two-tailed unpaired Student’s *t*-test. *p* < 0.05 was considered statistically significant.

## Supplementary information

supplemental data.

## Data Availability

The data supporting the findings of this study are available from the corresponding author upon reasonable request.
