# Supplementary material for: TDP43 is a newly identified substrate for PS1, enhancing the expression of APP following cleavage
Source: Cell Death Discov. 2025 Feb 23;11:76. doi: 10.1038/s41420-025-02340-z (PMC11847911; doi:10.1038/s41420-025-02340-z)

## Supplementary Figure 1 Sequencing results of TDP43 point mutations

**A. Sequence alignment of the TDP43 N390D point mutation** The asparagine codon AAT at position 390 of TDP43 is mutated to GAT. **B. Sequence alignment of the TDP43 G348C point mutation** The glycine codon GGC at position 348 of TDP43 is mutated to TGC. **C. Sequence alignment of the TDP43 A315T point mutation** The alanine codon GCG at position 315 of TDP43 is mutated to ACG.

## Supplementary Figure 2 depicts the mutational energies of amino acid substitutions at positions 390, 348, and 315 of TDP43

**A. The mutation energy when asparagine at position 390 of TDP43 is substituted with the remaining 24 amino acids** The mutation energy for the asparagine at position 390 being mutated to aspartic acid is -0.17 Kcal/mol, with no impact on the affinity between TDP43 and PS1 complex. **B. The mutation energy when glycine at position 348 of TDP43 is substituted with the remaining 24 amino acids** The mutation energy for glycine at position 348 being mutated to cysteine is 0 Kcal/mol, with no impact on the affinity between TDP43 and PS1 complex. **C. The mutation energy when alanine at position 315 of TDP43 is substituted with the remaining 24 amino acids** The mutation energy for alanine at position 315 being mutated to serine is -2.88 Kcal/mol, indicating an enhanced affinity between TDP43 and PS1.

## Supplementary Figure 3 the mutational energies of saturated amino acid substitutions at positions 385 and 257 of PS1 are presented

**A. The mutational energy when aspartic acid at position 385 of PS1 is substituted with the remaining 24 amino acids** The mutational energy for the substitution of aspartic acid at position 385 with alanine is 0.05 Kcal/mol, with no impact on the affinity between the TDP43 and PS1 complex. **B. The mutational energy when aspartic acid at position 257 of PS1 is substituted with the remaining 24 amino acids** The mutational energy for the substitution of aspartic acid at position 257 with alanine is 0.08 Kcal/mol, with no impact on the affinity between the TDP43 and PS1 complex.

## Supplementary Figure 4 Effect of transfecting TDP43 and PS1 following the knockdown of PS1 on Aβ content

**A and C. Effect of TDP43 overexpression in PS1 knockdown HELA and NSC34 cells on Aβ content.** The initial step involved the knockdown of PS1 in HeLa and NSC34 cells using siRNA. This was followed by the transfection of TDP43 and PS1, and the subsequent detection of Aβ content in HeLa (**A**) and NSC34 (**C**) cells through immunofluorescence. DAPI was used to stain the cell nuclei and Aβ was labeled with FITC to emit green fluorescence. **E and G. Effect of TDP43 overexpression in PS1 knockdown primary cells on Aβ content.** The initial step involved the knockdown of PS1 in MEF and primary neuron cells using siRNA. This was followed by the transfection of TDP43 and PS1, and the detection of Aβ content in MEF (**E**) and primary neuron cells (**G**) through immunofluorescence. DAPI was used to stain the cell nuclei and Aβ was labeled with FITC to emit green fluorescence. **B, D, F and H. Statistical graphs illustrating Aβ fluorescence intensity.** The fluorescence intensity of Aβ and the total area of all cells in the DAPI channel were calculated using the ImageJ software. The ratio of Aβ

fluorescence intensity to the total area of the cells is the average fluorescence intensity of Aβ in HeLa (**B**), NSC34 (**D**), MEF (**F**) and primary neuron cells (**H**). Date are the mean ± SD of at least three independent experiments. (*p < 0.05, **p < 0.01, ***p < 0.001, ****p < 0.0001, ns: not significant).

## Supplementary Figure 5 Overexpression of TDP43 has been observed to results in an increase in the levels of both APP and Aβ in AD primary neuron cells

**A and B. Morphological changes that occur in MEF cells before and after they are induced to differentiate into primary neuron cells.** MEF cells and NGF-induced primary neuron cells were selected for immunofluorescence experiments with MAP2 and β-tubulin: MAP2 labeling of neuron cells, red fluorescence with R-PE, β-tubulin labeling of the cytoskeleton, green fluorescence with FITC, and DAPI labeling of the nucleus (**A**). The fluorescence intensity of MAP2 and the total area of all cells in the DAPI channel were calculated using the ImageJ software. The ratio of MAP2 fluorescence intensity to the total area of the cells is the average fluorescence intensity of MAP2. Date are the mean ± SD of at least three independent experiments. Differences between two groups were analysed by a two-tailed unpaired Student's *t* test (*p < 0.05, **p < 0.01, ***p < 0.001, ****p < 0.0001, ns: not significant). **C - F. Overexpression of TDP43 in AD results in elevated levels of APP and Aβ.** TDP43 was transfected into AD primary neurons, and 24 h later, the levels of APP were quantified by WB (**C**), while the levels of Aβ were determined by immunofluorescence (**E**). The grayscale values for APP in AD primary neuron cells (**D**) are the mean ± SD of at least three independent experiments. Differences between two groups were analysed by a two-tailed unpaired Student's *t* test (*p < 0.05, **p < 0.01, ***p < 0.001, ****p < 0.0001, ns: not significant). The average fluorescence intensity of Aβ (**F**) are the mean ± SD of at least three independent experiments (*p < 0.05, **p < 0.01, ***p < 0.001, ****p < 0.0001, ns: not significant).

**Supplementary Table 1 Interface interaction analysis of TDP43-PS1 complex**
**Supplementary Table 2 N390 Mutation Energy**
**Supplementary Table 3 G348 Mutation Energy**
**Supplementary Table 4 D385 Mutation Energy**
**Supplementary Table 5 D257 Mutation Energy**
**Supplementary Table 6 A315 Mutation Energy**

A

N390D

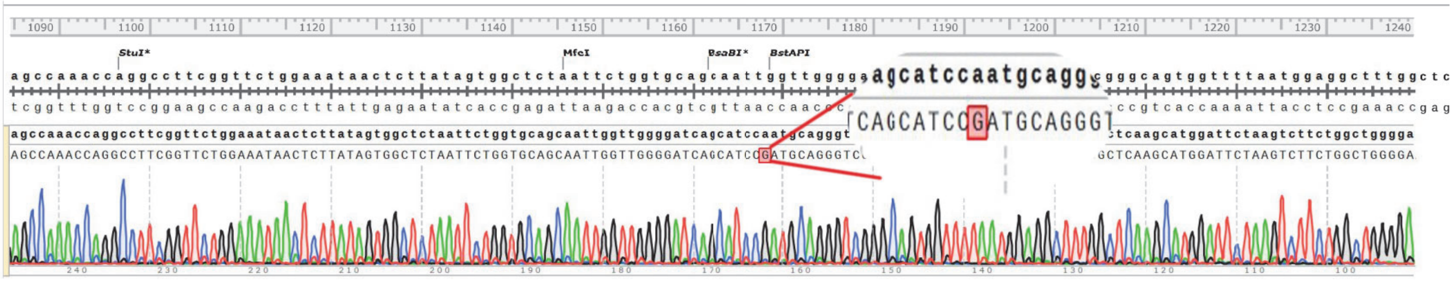


B

G348C

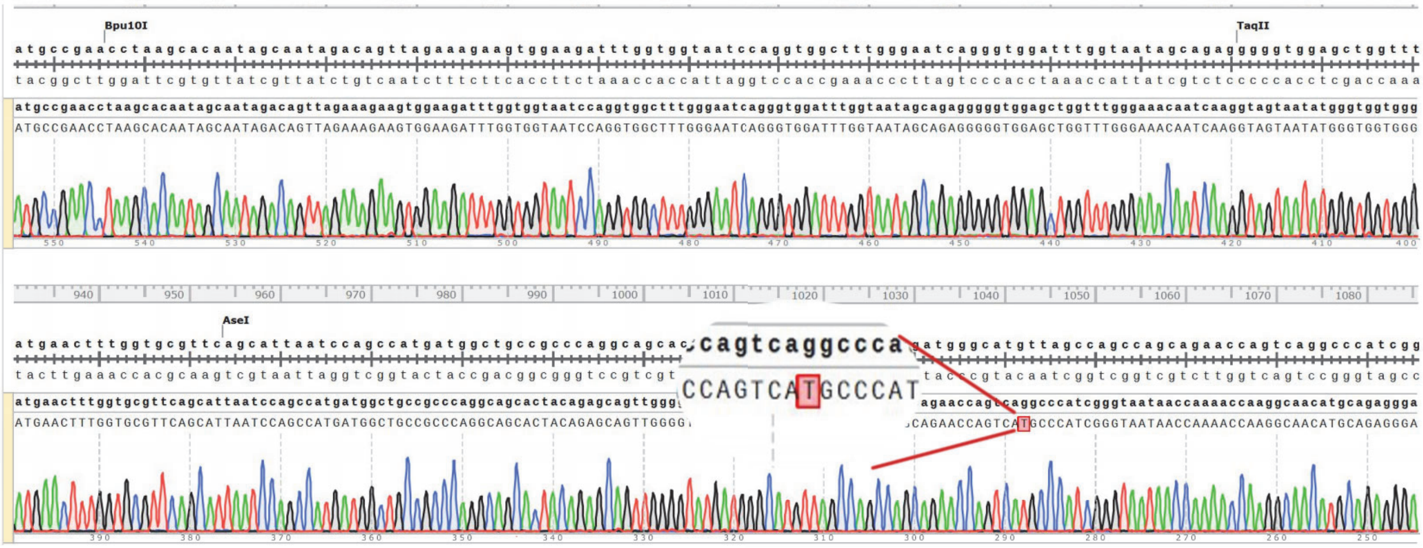


C

A315T

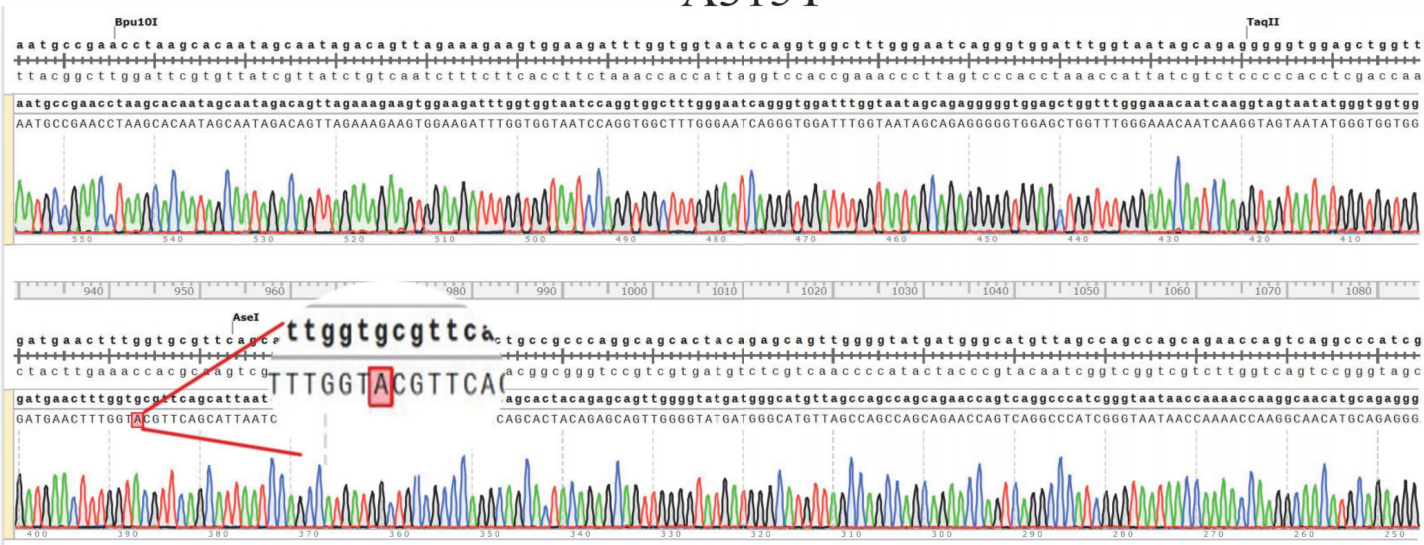


**Supplementary Figure 1 Sequencing results of TDP43 point mutations**

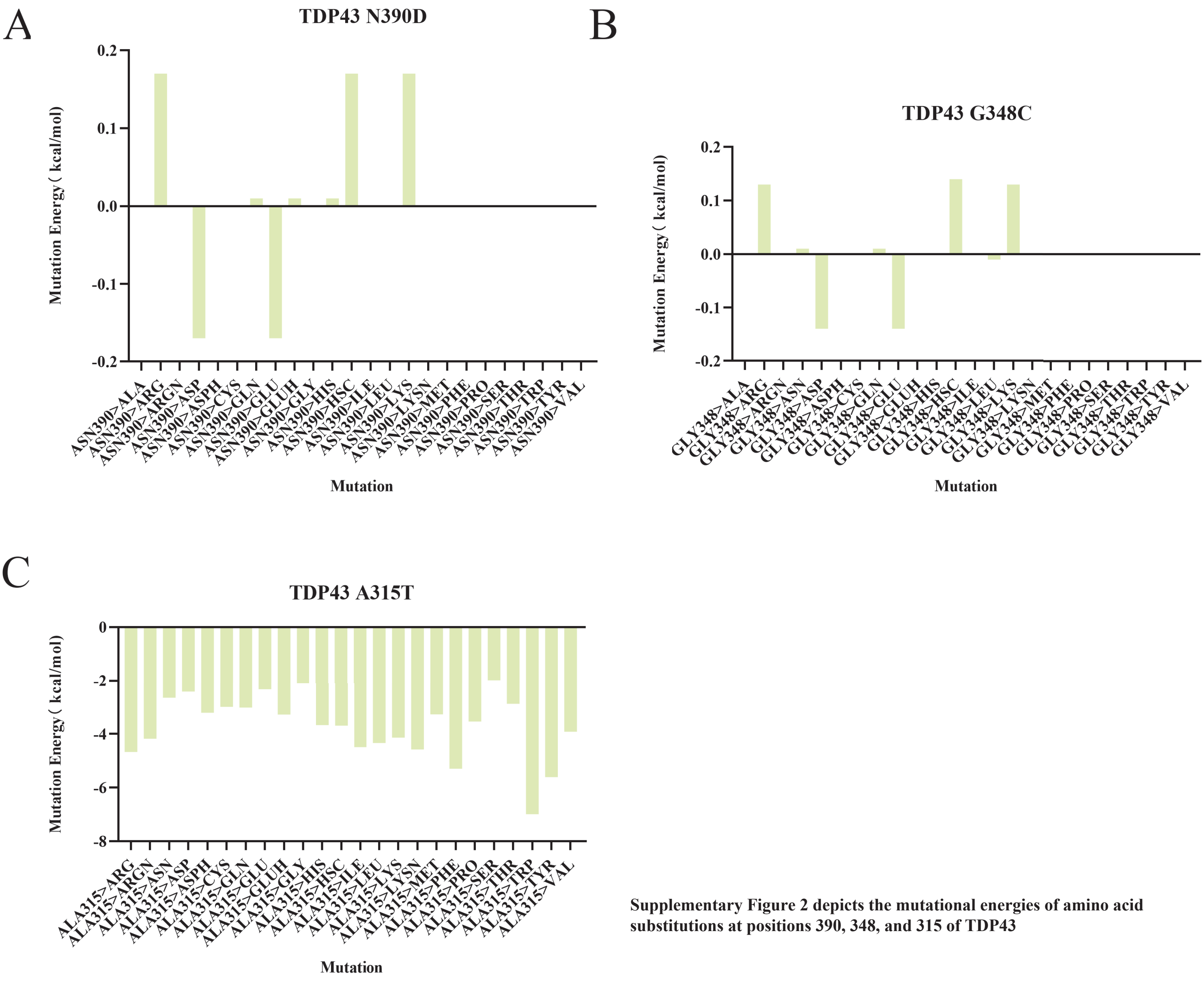


**Supplementary Figure 2 depicts the mutational energies of amino acid substitutions at positions 390, 348, and 315 of TDP43**

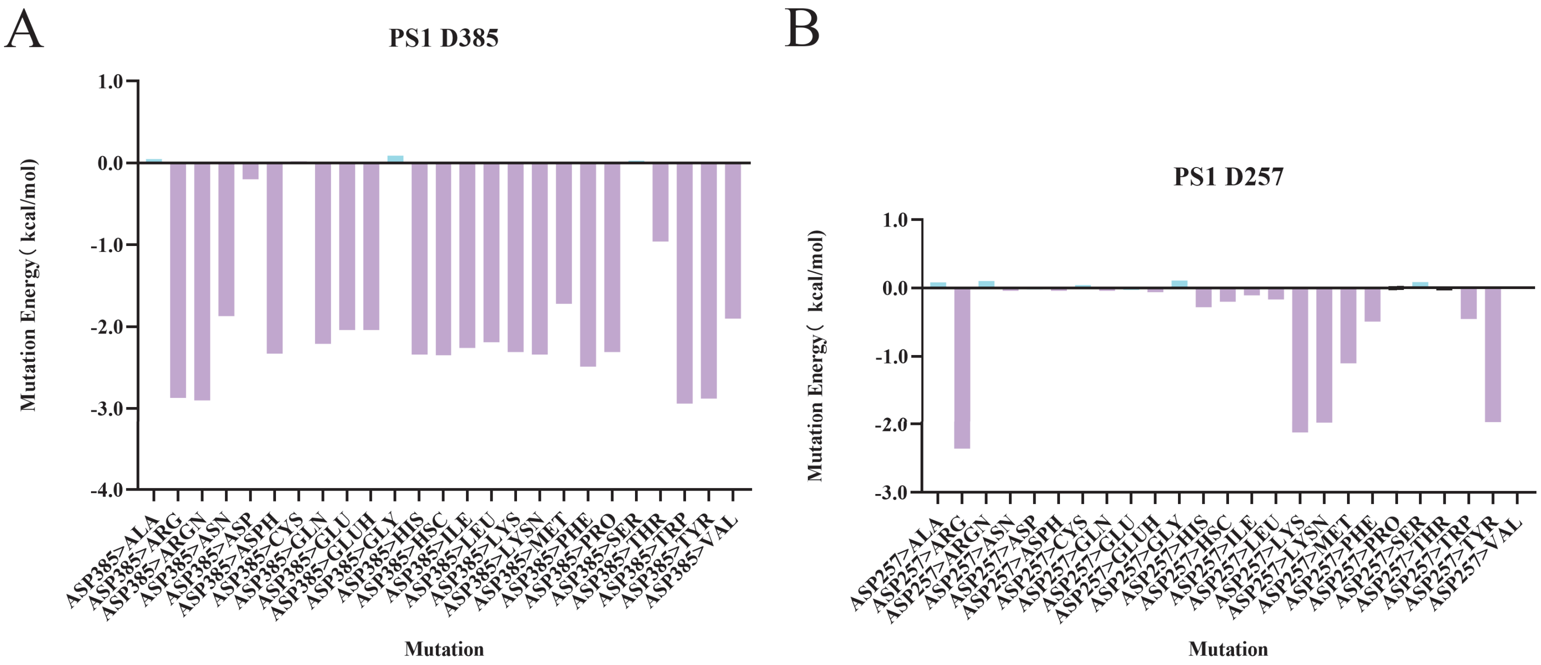


**Supplementary Figure 3 the mutational energies of saturated amino acid substitutions at positions 385 and 257 of PS1 are presented**

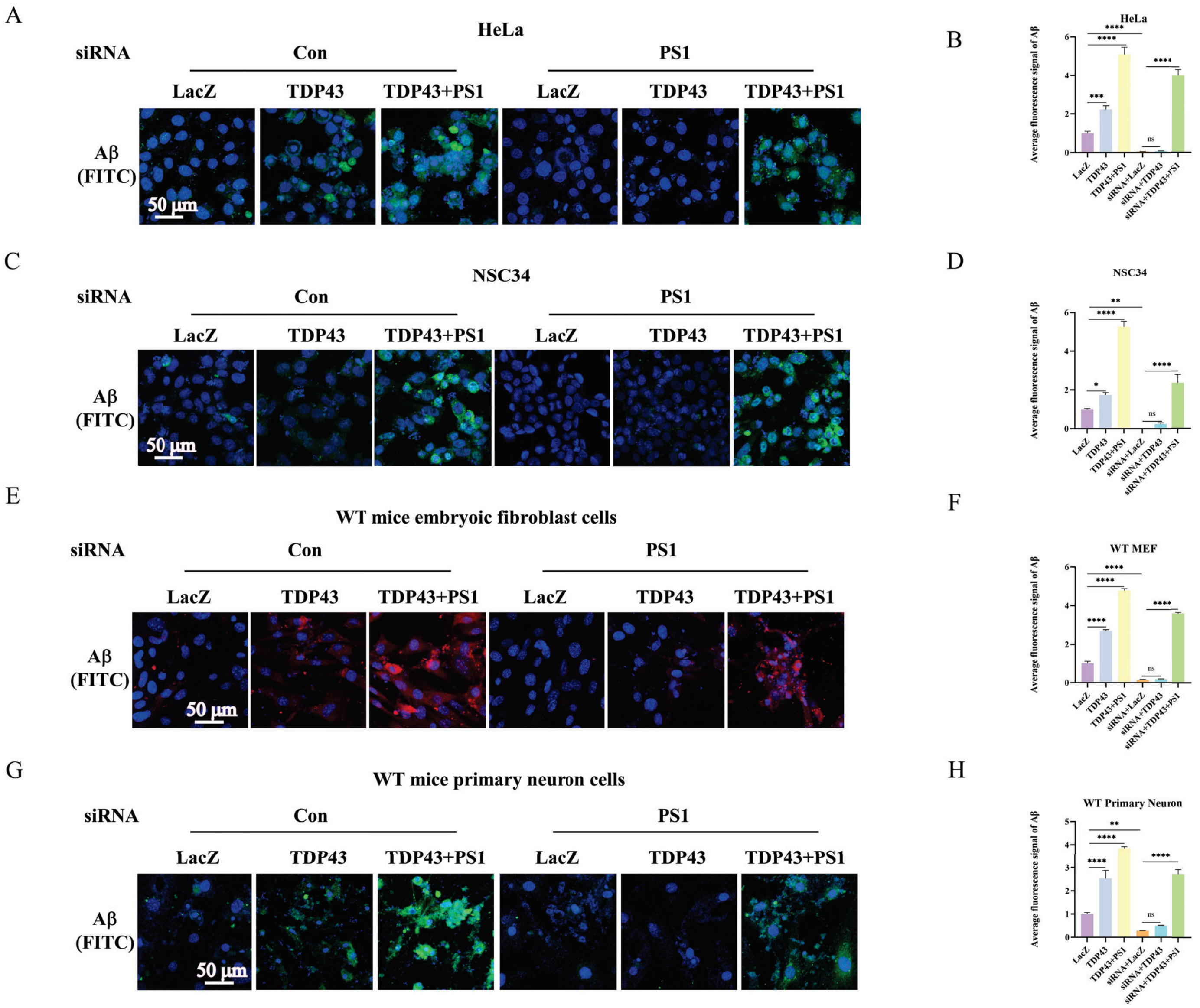
A
HeLa
siRNA
Con
PS1
LacZ
TDP43
TDP43+PS1
LacZ
TDP43
TDP43+PS1
Aβ (FITC)
50 μm
B
HeLa
Average fluorescence signal of Aβ
LacZ
TDP43
TDP43+PS1
siRNA+LacZ
siRNA+TDP43
siRNA+TDP43+PS1
C
NSC34
siRNA
Con
PS1
LacZ
TDP43
TDP43+PS1
LacZ
TDP43
TDP43+PS1
Aβ (FITC)
50 μm
D
NSC34
Average fluorescence signal of Aβ
E
WT mice embryoic fibroblast cells
siRNA
Con
PS1
LacZ
TDP43
TDP43+PS1
LacZ
TDP43
TDP43+PS1
Aβ (FITC)
50 μm
F
WT MEF
Average fluorescence signal of Aβ
G
WT mice primary neuron cells
siRNA
Con
PS1
LacZ
TDP43
TDP43+PS1
LacZ
TDP43
TDP43+PS1
Aβ (FITC)
50 μm
H
WT Primary Neuron
Average fluorescence signal of Aβ

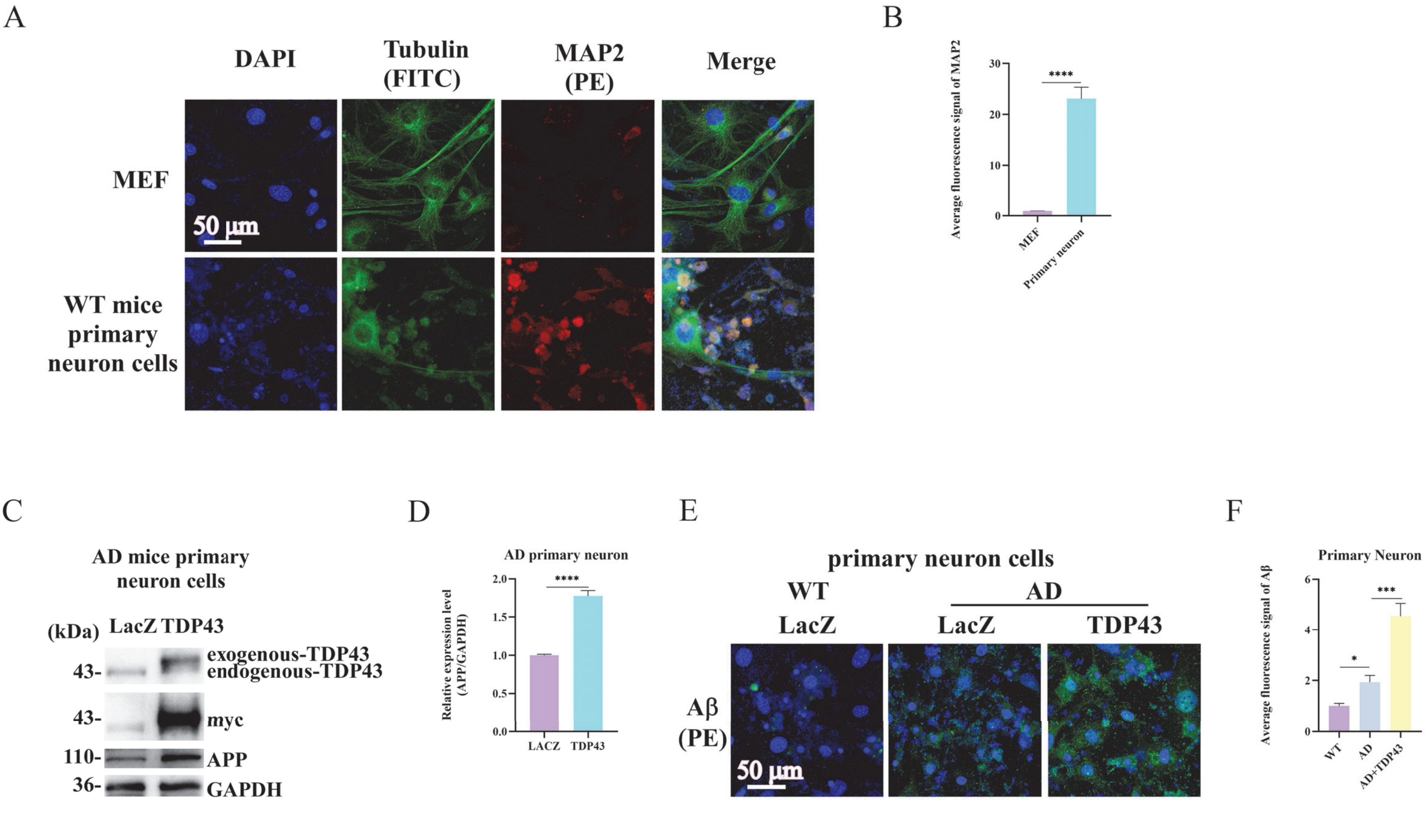


**Supplementary Figure 5 Overexpression of TDP43 has been observed to results in an increase in the levels of both APP and Aβ in AD primary neuron cells**

**Supplementary Table 1 Interface interaction analysis of TDP43-PS1 complex**

| ResidueName | A:LYS140 | A:PHE194 | A:ARG227 | A:PHE229 | A:ASN265 | A:SER266 | A:ASN267 | A:LEU270 |
|---|---|---|---|---|---|---|---|---|
| B:THR74 | - | - | - | - | - | - | - | - |
| B:LEU75 | - | - | - | - | - | - | - | - |
| B:GLY78 | - | - | - | - | - | - | - | - |
| B:VAL82 | - | - | - | - | - | - | - | - |
| B:ILE114 | - | - | - | - | - | - | - | - |
| B:TYR115 | - | - | - | - | - | - | - | - |
| B:THR116 | - | - | - | - | - | - | - | - |
| B:ASN135 | - | - | - | - | - | - | - | - |
| B:ILE138 | - | - | - | - | - | - | - | - |
| B:MET139 | - | - | - | - | - | - | - | - |
| B:VAL142 | - | - | - | - | - | - | - | - |
| B:VAL145 | - | - | - | - | - | - | - | - |
| B:MET146 | - | - | - | - | - | - | - | - |
| B:LYS160 | - | Pi | N | N | - | - | - | - |
| B:HIS163 | - | - | Pi | - | - | - | - | - |
| B:ALA164 | - | - | N | - | - | - | - | - |
| B:LEU171 | - | - | - | - | - | N | - | - |
| B:LEU173 | - | - | - | - | - | - | - | - |
| B:PHE175 | - | - | - | - | - | N | N | N |
| B:PHE176 | - | - | - | - | - | - | - | Pi |
| B:PHE179 | - | - | - | - | - | - | - | Pi |
| B:VAL193 | - | - | - | - | - | - | - | - |
| B:ASP194 | N | - | - | - | - | - | - | - |
| B:TYR195 | - | - | - | - | - | - | - | - |
| B:ILE196 | N | - | - | - | - | - | - | - |
| B:VAL198 | - | - | - | - | - | - | - | - |
| B:ALA199 | - | - | - | - | - | - | - | - |
| B:ILE202 | - | - | - | - | - | - | N | N |
| B:TRP203 | - | - | - | - | N | - | H | - |
| B:VAL207 | - | - | - | - | - | - | N | - |
| B:ILE229 | - | - | - | - | - | - | - | - |
| B:MET233 | - | - | - | - | - | - | - | - |
| B:VAL236 | - | - | - | - | - | - | - | - |
| B:PHE237 | - | - | - | - | - | - | - | - |
| B:TYR240 | - | - | - | - | - | - | - | - |
| B:LEU241 | - | - | - | - | - | - | - | - |
| B:LEU268 | - | - | - | - | - | - | - | - |
| B:VAL272 | - | - | - | - | - | - | - | - |
| B:THR281 | - | - | - | - | - | - | - | - |
| B:LEU282 | - | - | - | - | - | - | - | - |
| B:PHE283 | - | - | - | - | - | - | - | - |
| B:PRO284 | - | - | - | - | - | - | - | - |
| B:ALA285 | - | - | - | - | - | - | - | - |
| B:LEU286 | - | - | - | - | - | - | - | - |
| B:ILE287 | - | - | - | - | - | - | - | - |

| | | | | | | | | |
|---|---|---|---|---|---|---|---|---|
| B:SER289 | - | - | - | - | - | - | - | - |
| B:THR291 | - | - | - | - | - | - | - | - |
| B:GLY378 | - | - | - | - | - | - | - | - |
| B:VAL379 | - | - | - | - | - | - | - | - |
| B:LYS380 | - | - | - | - | - | - | - | - |
| B:LEU381 | - | - | - | - | - | - | - | - |
| B:GLY382 | - | - | - | - | - | - | - | - |
| B:LEU383 | - | - | - | - | - | - | - | - |
| B:GLY384 | - | - | - | - | - | - | - | - |
| B:ILE387 | - | - | - | - | - | - | - | - |
| B:PHE388 | - | - | - | - | - | - | - | - |
| B:ASP403 | - | - | - | - | - | - | - | - |
| B:TRP404 | - | - | - | - | - | - | - | - |
| B:ASN405 | - | - | - | - | - | - | - | - |
| B:ILE408 | - | - | - | - | - | - | - | - |
| B:ALA409 | - | - | - | - | - | - | - | - |
| B:VAL412 | - | - | - | - | - | - | - | - |
| B:ILE416 | - | - | - | - | - | - | - | - |
| B:CYS419 | - | - | - | - | - | - | - | - |
| B:LEU422 | - | - | - | - | - | - | - | - |
| B:LEU423 | - | - | - | - | - | - | - | - |
| B:LEU425 | - | - | - | - | - | - | - | - |
| B:ALA426 | - | - | - | - | - | - | - | - |
| B:ILE427 | - | - | - | - | - | - | - | - |

## AminoAcid composition

| ResidueTypes | Percentages A | Percentages at Interface |
|---|---|---|
| A | 7.17 | 8.04 |
| C | 1.66 | 0.89 |
| D | 4.14 | 1.79 |
| E | 4.14 | 0.89 |
| F | 5.93 | 8.93 |
| G | 9.66 | 12.5 |
| H | 1.38 | 0.89 |
| I | 6.07 | 9.82 |
| K | 4.69 | 2.68 |
| L | 9.38 | 12.5 |
| M | 3.72 | 6.25 |
| N | 4.55 | 8.04 |
| P | 3.59 | 1.79 |
| Q | 4.28 | 0.89 |
| R | 3.72 | 2.68 |
| S | 7.86 | 4.46 |
| T | 4.97 | 3.57 |
| V | 8 | 8.93 |
| W | 1.66 | 1.79 |

| | | |
|---|---|---|
| Y | 3.45 | 2.68 |
| X | 0 | 0 |

## PI Intreactions

| ReceptorResi | LigandResidu | Interaction Co | Distance | Type |
|---|---|---|---|---|
| B:MET233 | A:PHE316 | B:MET233:SI | 5.1655 | Pi-Sulfur |
| B:PHE179 | A:LEU270 | A:LEU270:O | 2.2759 | Pi-Lone Pair |
| B:PHE283 | A:PHE313 | A:PHE313 - I | 5.8622 | Pi-Pi T-shaped |
| B:TYR195 | A:ARG272 | A:ARG272:C | 5.2671 | Pi-Amide Stacked |
| B:LYS160 | A:PHE194 | A:PHE194 - I | 5.1405 | Pi-Alkyl |
| B:ALA285 | A:PHE313 | A:PHE313 - I | 4.5214 | Pi-Alkyl |
| B:HIS163 | A:ARG227 | B:HIS163 - A | 4.7065 | Pi-Alkyl |
| B:PHE176 | A:LEU270 | B:PHE176 - A | 5.1275 | Pi-Alkyl |
| B:PHE179 | A:LEU270 | B:PHE179 - A | 5.1645 | Pi-Alkyl |
| B:TYR195 | A:ARG275 | B:TYR195 - A | 4.7674 | Pi-Alkyl |
| B:TRP404 | A:ALA297 | B:TRP404 - A | 3.7125 | Pi-Alkyl |
| B:TRP404 | A:ALA297 | B:TRP404 - A | 5.2367 | Pi-Alkyl |

## Hydrogen Bonds

| ReceptorResi | LigandResidu | Interaction Co | Distance | Type |
|---|---|---|---|---|
| B:TRP203 | A:ASN267 | A:ASN267:N | 2.9667 | Conventional |
| B:TYR240 | A:ASN319 | A:ASN319:N | 3.3964 | Conventional |
| B:ILE202 | A:GLU271 | B:ILE202:N - | 3.2526 | Conventional |
| B:GLY378 | A:GLY309 | A:GLY309:C | 3.072 | Carbon |
| B:GLY384 | A:ALA315 | B:GLY384:C. | 3.0389 | Carbon |

## Contact Surface Area

| Residue | Contact Surfa | Polar Contact | Nonpolar Contact Surface Area |
|---|---|---|---|
| A:LYS140 | 9.80765 | 9.80766 | 0 |
| A:PHE194 | 16.0651 | 0 | 16.0651 |
| A:ARG227 | 35.421299 | 31.5436 | 3.87777 |
| A:PHE229 | 0.55397 | 0 | 0.553967 |
| A:ASN265 | 16.7083 | 16.7083 | 0 |
| A:SER266 | 0.830948 | 0 | 0.830952 |
| A:ASN267 | 85.585403 | 71.182198 | 14.4032 |
| A:LEU270 | 89.101303 | 21.7943 | 67.307098 |
| A:GLU271 | 100.223999 | 71.971802 | 28.2523 |
| A:ARG272 | 4.00146 | 1.7856 | 2.21587 |
| A:SER273 | 8.9987 | 8.16775 | 0.830952 |
| A:GLY274 | 17.431601 | 10.2301 | 7.20158 |
| A:ARG275 | 18.441799 | 8.74737 | 9.69443 |
| A:GLY295 | 10.2284 | 5.79663 | 4.43174 |
| A:GLY296 | 39.925301 | 35.493599 | 4.43174 |
| A:ALA297 | 32.926102 | 12.4293 | 20.496799 |

| | | | |
|---|---|---|---|
| A:GLY298 | 50.159698 | 37.972401 | 12.1873 |
| A:LEU299 | 17.1611 | 0.265072 | 16.896 |
| A:GLY300 | 37.472099 | 33.594398 | 3.87777 |
| A:ASN301 | 5.91653 | 2.31575 | 3.60079 |
| A:ASN302 | 26.1689 | 17.3055 | 8.86348 |
| A:GLN303 | 8.22729 | 6.84239 | 1.38492 |
| A:GLY304 | 37.055302 | 33.454601 | 3.60079 |
| A:SER305 | 11.2858 | 6.85404 | 4.43174 |
| A:ASN306 | 66.006203 | 47.171299 | 18.8349 |
| A:MET307 | 95.991798 | 27.6579 | 68.333801 |
| A:GLY308 | 32.148701 | 26.886 | 5.26269 |
| A:GLY309 | 19.3412 | 1.06029 | 18.280899 |
| A:GLY310 | 13.2714 | 0.530143 | 12.7413 |
| A:MET311 | 86.754501 | 13.1837 | 73.5709 |
| A:ASN312 | 35.2271 | 30.518299 | 4.70872 |
| A:PHE313 | 127.633003 | 6.86857 | 120.764999 |
| A:GLY314 | 37.677799 | 28.814301 | 8.86348 |
| A:ALA315 | 23.856501 | 17.7628 | 6.09364 |
| A:PHE316 | 45.702301 | 0 | 45.702301 |
| A:SER317 | 57.166302 | 40.8242 | 16.341999 |
| A:ILE318 | 19.1471 | 12.2225 | 6.92459 |
| A:ASN319 | 56.330898 | 48.575298 | 7.75555 |
| A:PRO320 | 56.1096 | 1.54383 | 54.5658 |
| A:ALA321 | 52.4142 | 9.20474 | 43.209499 |
| A:MET322 | 8.99753 | 3.18086 | 5.81666 |
| A:MET323 | 35.7738 | 0 | 35.7738 |
| A:ALA324 | 2.21587 | 0 | 2.21587 |
| B:THR74 | 23.520599 | 12.1642 | 11.3563 |
| B:LEU75 | 1.03726 | 0.760265 | 0.276984 |
| B:GLY78 | 15.4396 | 1.59043 | 13.8492 |
| B:VAL82 | 11.6333 | 0 | 11.6333 |
| B:ILE114 | 42.655998 | 11.9108 | 30.745199 |
| B:TYR115 | 4.89119 | 1.29041 | 3.60079 |
| B:THR116 | 10.8024 | 0 | 10.8024 |
| B:ASN135 | 5.32186 | 5.32186 | 0 |
| B:ILE138 | 10.3369 | 2.02737 | 8.30951 |
| B:MET139 | 51.379299 | 1.32536 | 50.054001 |
| B:VAL142 | 28.712099 | 1.01369 | 27.6984 |
| B:VAL145 | 5.53967 | 0 | 5.53968 |
| B:MET146 | 5.26163 | 0 | 5.26164 |
| B:LYS160 | 15.9102 | 3.44593 | 12.4643 |
| B:HIS163 | 26.9077 | 3.64111 | 23.2666 |
| B:ALA164 | 0.265072 | 0.265072 | 0 |
| B:LEU171 | 0.830955 | 0 | 0.830952 |
| B:LEU173 | 7.47856 | 0 | 7.47856 |
| B:PHE175 | 33.2621 | 11.6574 | 21.6047 |
| B:PHE176 | 14.6208 | 1.04864 | 13.5722 |

| | | | |
|---|---|---|---|
| B:PHE179 | 39.6087 | 0 | 39.6087 |
| B:VAL193 | 5.8287 | 5.8287 | 0 |
| B:ASP194 | 3.04106 | 3.04106 | 0 |
| B:TYR195 | 67.290802 | 32.944801 | 34.346001 |
| B:ILE196 | 6.37062 | 0 | 6.37063 |
| B:VAL198 | 7.21938 | 2.78764 | 4.43174 |
| B:ALA199 | 14.1199 | 3.59451 | 10.5254 |
| B:ILE202 | 10.7788 | 0.253422 | 10.5254 |
| B:TRP203 | 50.847198 | 1.26711 | 49.580101 |
| B:VAL207 | 1.64999 | 0.265072 | 1.38492 |
| B:ILE229 | 9.69443 | 0 | 9.69443 |
| B:MET233 | 21.024099 | 0 | 21.024099 |
| B:VAL236 | 2.02737 | 2.02737 | 0 |
| B:PHE237 | 55.950699 | 0 | 55.950699 |
| B:TYR240 | 9.3588 | 6.58897 | 2.76984 |
| B:LEU241 | 14.9571 | 0 | 14.9571 |
| B:LEU268 | 8.30951 | 0 | 8.30951 |
| B:VAL272 | 1.10793 | 0 | 1.10793 |
| B:THR281 | 2.76984 | 0 | 2.76984 |
| B:LEU282 | 6.09364 | 0 | 6.09364 |
| B:PHE283 | 9.35212 | 4.36642 | 4.98571 |
| B:PRO284 | 1.26711 | 1.26711 | 0 |
| B:ALA285 | 4.05475 | 4.05475 | 0 |
| B:LEU286 | 37.4818 | 13.9382 | 23.5436 |
| B:ILE287 | 44.4175 | 2.03902 | 42.378502 |
| B:SER289 | 1.52053 | 1.52053 | 0 |
| B:THR291 | 5.53968 | 0 | 5.53967 |
| B:GLY378 | 9.37661 | 9.37661 | 0 |
| B:VAL379 | 12.7412 | 0 | 12.7413 |
| B:LYS380 | 69.831299 | 25.513901 | 44.317402 |
| B:LEU381 | 2.42217 | 0.760265 | 1.6619 |
| B:GLY382 | 14.838 | 2.65072 | 12.1873 |
| B:LEU383 | 7.9015 | 2.91579 | 4.98571 |
| B:GLY384 | 24.1989 | 10.0727 | 14.1262 |
| B:ILE387 | 14.1262 | 0 | 14.1262 |
| B:PHE388 | 11.3563 | 0 | 11.3563 |
| B:ASP403 | 27.2493 | 12.5691 | 14.6801 |
| B:TRP404 | 30.5012 | 8.3425 | 22.158701 |
| B:ASN405 | 32.023399 | 26.7607 | 5.26269 |
| B:ILE408 | 46.828098 | 2.78764 | 44.040401 |
| B:ALA409 | 3.54149 | 1.04864 | 2.49285 |
| B:VAL412 | 44.270302 | 0.506843 | 43.763401 |
| B:ILE416 | 45.401501 | 0.530144 | 44.871399 |
| B:CYS419 | 33.6339 | 7.34923 | 26.2847 |
| B:LEU422 | 21.469601 | 10.3903 | 11.0793 |
| B:LEU423 | 34.8932 | 4.14795 | 30.745199 |
| B:LEU425 | 23.797001 | 0.253422 | 23.5436 |

| | | | |
|---|---|---|---|
| B:ALA426 | 26.760201 | 2.38565 | 24.374599 |
| B:ILE427 | 0.276977 | 0 | 0.276985 |

| A:GLU271 | A:ARG272 | A:SER273 | A:GLY274 | A:ARG275 | A:GLY295 | A:GLY296 | A:ALA297 | A:GLY298 | A:LEU299 | A:GLY300 |
|---|---|---|---|---|---|---|---|---|---|---|
| - | - | - | - | - | - | - | - | - | - | - |
| - | - | - | - | - | - | - | - | - | - | - |
| - | - | - | - | - | - | - | - | - | - | - |
| - | - | - | - | - | - | - | - | - | - | - |
| - | - | - | - | - | - | - | - | - | - | - |
| - | - | - | - | - | - | - | - | - | - | - |
| - | - | - | - | - | - | - | - | - | - | - |
| - | - | - | - | - | - | - | - | - | - | - |
| - | - | - | - | - | - | - | - | - | - | - |
| - | - | - | - | - | - | - | - | - | - | - |
| - | - | - | - | - | - | - | - | - | - | - |
| - | - | - | - | - | - | - | - | - | - | - |
| - | - | - | - | - | - | - | - | - | - | - |
| - | - | - | - | - | - | - | - | - | - | - |
| - | - | - | - | - | - | - | - | - | - | - |
| - | - | - | - | - | - | - | - | - | - | - |
| - | - | - | - | - | - | - | - | - | - | - |
| - | - | - | - | - | - | - | - | - | - | - |
| - | - | - | - | - | - | - | - | - | - | - |
| - | - | - | - | - | - | - | - | - | - | - |
| N | N | N | N | - | - | - | - | - | - | - |
| - | - | - | - | N | - | - | - | - | - | - |
| - | - | - | - | - | - | - | - | - | - | - |
| N | Pi | N | N | Pi | - | - | - | - | - | - |
| - | - | - | - | - | - | - | - | - | - | - |
| N | - | - | - | - | - | - | - | - | - | - |
| N | - | - | - | - | - | - | - | - | - | - |
| H | - | - | - | - | - | - | - | - | - | - |
| N | - | - | - | - | - | - | - | - | - | - |
| - | - | - | - | - | - | - | - | - | - | - |
| - | - | - | - | - | - | - | - | - | - | - |
| - | - | - | - | - | - | - | - | - | - | - |
| - | - | - | - | - | - | - | - | - | - | - |
| - | - | - | - | - | - | - | - | - | - | - |
| - | - | - | - | - | - | - | - | - | - | - |
| - | - | - | - | - | - | - | - | - | - | - |
| - | - | - | - | - | - | - | - | - | - | - |
| - | - | - | - | - | - | - | - | - | - | - |
| - | - | - | - | - | - | - | - | - | - | - |
| - | - | - | - | - | - | - | - | - | - | - |
| - | - | - | - | - | - | - | - | - | - | - |
| - | - | - | - | - | - | - | - | - | - | - |
| - | - | - | - | - | - | - | - | - | - | - |
| - | - | - | - | - | - | - | - | - | - | - |
| - | - | - | - | - | - | - | - | - | - | - |

| | | | | | | | | | | |
|---|---|---|---|---|---|---|---|---|---|---|
| - | - | - | - | - | - | - | - | - | - | - |
| - | - | - | - | - | - | - | - | - | - | - |
| - | - | - | - | - | - | - | - | - | - | - |
| - | - | - | - | - | - | - | - | - | - | - |
| - | - | - | - | - | - | - | - | - | - | - |
| - | - | - | - | - | - | - | - | - | - | - |
| - | - | - | - | - | - | - | - | - | - | - |
| - | - | - | - | - | - | - | - | - | - | - |
| - | - | - | - | - | - | - | - | - | - | - |
| - | - | - | - | - | - | - | - | - | - | - |
| - | - | - | - | - | - | - | - | - | - | - |
| - | - | - | - | - | N | N | N | - | - | - |
| - | - | - | - | - | - | N | Pi | N | N | - |
| - | - | - | - | - | - | N | N | N | N | - |
| - | - | - | - | - | - | - | N | N | N | N |
| - | - | - | - | - | - | - | - | N | N | N |
| - | - | - | - | - | - | - | - | - | N | N |
| - | - | - | - | - | - | - | - | - | - | - |
| - | - | - | - | - | - | - | - | - | - | - |
| - | - | - | - | - | - | - | - | - | - | - |
| - | - | - | - | - | - | - | - | - | - | - |
| - | - | - | - | - | - | - | - | - | - | - |
| - | - | - | - | - | - | - | - | - | - | - |
| - | - | - | - | - | - | - | - | - | - | - |

## Interface Interactions

| A:ASN301 | A:ASN302 | A:GLN303 | A:GLY304 | A:SER305 | A:ASN306 | A:MET307 | A:GLY308 | A:GLY309 | A:GLY310 | A:MET311 |
|---|---|---|---|---|---|---|---|---|---|---|
| - | - | - | - | - | N | N | - | - | - | - |
| - | - | - | - | - | - | N | - | - | - | - |
| - | - | - | - | - | N | N | - | - | - | - |
| - | - | - | N | N | N | N | - | - | - | - |
| - | - | - | - | - | - | - | - | - | - | - |
| - | - | - | - | - | - | - | - | - | - | - |
| - | - | - | - | - | - | - | - | - | - | - |
| - | - | - | - | - | - | - | - | - | - | - |
| - | - | - | - | - | - | - | - | - | - | - |
| - | - | - | - | - | - | - | - | - | - | - |
| - | - | - | - | - | - | - | - | - | - | - |
| - | - | - | - | - | - | - | - | - | - | - |
| - | - | - | - | - | - | - | - | - | - | - |
| - | - | - | - | - | - | - | - | - | - | - |
| - | - | - | - | - | - | - | - | - | - | - |
| - | - | - | - | - | - | - | - | - | - | - |
| - | - | - | - | - | - | - | - | - | - | - |
| - | - | - | - | - | - | - | - | - | - | - |
| - | - | - | - | - | - | - | - | - | - | - |
| - | - | - | - | - | - | - | - | - | - | - |
| - | - | - | - | - | - | - | - | - | - | - |
| - | - | - | - | - | - | - | - | - | - | - |
| - | - | - | - | - | - | - | - | - | - | - |
| - | - | - | - | - | - | - | - | - | - | - |
| - | - | - | - | - | - | - | - | - | - | - |
| - | - | - | - | - | - | - | - | - | - | - |
| - | - | - | - | - | - | - | - | - | - | - |
| - | - | - | - | - | - | - | - | - | - | - |
| - | - | - | - | - | - | - | - | - | - | - |
| - | - | - | - | - | - | - | - | - | - | - |
| - | - | - | - | - | - | - | - | - | - | - |
| - | - | - | - | - | - | - | - | - | - | - |
| - | - | - | - | - | - | - | - | - | - | - |
| - | - | - | - | - | - | - | - | - | - | - |
| - | - | - | - | - | - | - | - | - | - | - |
| - | - | - | - | - | - | - | - | - | - | - |
| - | - | - | - | - | - | - | - | - | - | - |
| - | - | - | - | - | - | - | - | - | - | - |
| - | - | - | - | - | - | - | - | - | - | N |
| - | - | - | - | - | - | - | - | - | - | N |
| - | - | - | - | - | - | - | - | - | - | - |
| - | - | - | - | - | - | - | - | - | - | - |
| - | - | - | - | - | - | - | - | - | - | - |
| - | - | - | - | - | - | - | - | - | - | - |
| - | - | - | - | - | - | - | - | - | - | N |

| | | | | | | | | | | |
|---|---|---|---|---|---|---|---|---|---|---|
| - | - | - | - | - | - | - | - | - | - | N |
| - | - | - | - | - | - | - | - | - | - | N |
| - | - | - | - | - | - | - | - | H | N | - |
| - | - | - | - | - | - | - | N | N | N | N |
| - | - | - | - | - | - | - | - | N | N | N |
| - | - | - | - | - | - | - | - | - | - | N |
| - | - | - | - | - | - | - | - | - | - | - |
| - | - | - | - | - | - | - | - | - | - | - |
| - | - | - | - | - | - | - | - | - | - | - |
| - | - | - | - | - | - | - | - | - | - | - |
| - | - | - | - | - | - | - | - | - | - | - |
| - | - | - | - | - | - | - | - | - | - | - |
| - | - | - | - | - | - | - | - | - | - | - |
| - | - | - | - | - | - | - | - | - | - | - |
| - | - | - | - | - | - | - | - | - | - | - |
| - | - | - | - | - | - | - | - | - | - | - |
| N | N | - | - | - | - | - | - | - | - | - |
| N | N | N | N | - | - | - | - | - | - | - |
| - | - | N | N | N | N | - | - | - | - | - |
| - | - | - | - | - | N | N | N | - | - | - |
| - | - | - | - | N | N | N | - | - | - | - |
| - | - | - | - | - | - | N | N | N | - | - |
| - | - | - | - | - | N | N | N | - | - | - |
| - | - | - | - | - | N | N | - | - | - | - |

| A:ASN312 | A:PHE313 | A:GLY314 | A:ALA315 | A:PHE316 | A:SER317 | A:ILE318 | A:ASN319 | A:PRO320 | A:ALA321 | A:MET322 |
|---|---|---|---|---|---|---|---|---|---|---|
| - | - | - | - | - | - | - | - | - | - | - |
| - | - | - | - | - | - | - | - | - | - | - |
| - | - | - | - | - | - | - | - | - | - | - |
| - | - | - | - | - | - | - | - | - | - | - |
| - | - | - | - | - | - | - | N | N | N | N |
| - | - | - | - | - | - | - | - | N | N | - |
| - | - | - | - | - | - | - | - | - | N | - |
| - | - | - | - | - | - | - | - | N | - | - |
| - | - | - | - | - | - | - | - | N | N | - |
| - | - | - | - | - | N | N | N | N | - | - |
| - | - | - | - | - | - | N | - | N | - | - |
| - | - | - | - | - | - | - | - | - | - | - |
| - | - | - | - | - | - | N | - | - | - | - |
| - | - | - | - | - | - | - | - | - | - | - |
| - | - | - | - | - | - | - | - | - | - | - |
| - | - | - | - | - | - | - | - | - | - | - |
| - | - | - | - | - | - | - | - | - | - | - |
| - | - | - | - | N | - | - | - | - | - | - |
| - | - | - | - | - | - | - | - | - | - | - |
| - | - | - | - | - | - | - | - | - | - | - |
| - | - | - | - | - | - | - | - | - | - | - |
| - | - | - | - | - | - | - | - | - | - | - |
| - | - | - | - | - | - | - | - | - | - | - |
| - | - | - | - | - | - | - | - | - | - | - |
| - | - | - | - | - | - | - | - | - | - | - |
| - | - | - | - | - | - | - | - | - | - | - |
| - | - | - | - | - | - | - | - | - | - | - |
| - | - | - | - | - | - | - | - | - | - | - |
| - | - | - | - | - | - | - | - | - | - | - |
| - | - | - | - | - | - | - | - | - | - | - |
| - | - | - | - | N | - | - | - | - | - | - |
| - | - | - | - | Pi | N | - | - | - | - | - |
| - | - | - | - | - | - | - | N | - | - | - |
| - | - | - | - | N | N | N | N | - | - | - |
| - | - | - | - | - | - | - | H | - | - | - |
| - | - | - | - | - | - | - | N | N | - | - |
| N | - | - | - | - | - | - | - | - | - | - |
| N | - | - | - | - | - | - | - | - | - | - |
| - | - | - | - | - | - | - | - | - | - | - |
| N | N | - | - | - | - | - | - | - | - | - |
| - | Pi | - | - | - | - | - | - | - | - | - |
| - | N | - | - | - | - | - | - | - | - | - |
| - | Pi | - | - | - | - | - | - | - | - | - |
| N | N | N | N | N | - | - | - | - | - | - |
| N | N | N | - | - | - | - | - | - | - | - |

| | | | | | | | | | | |
|---|---|---|---|---|---|---|---|---|---|---|
| - | - | - | - | - | - | - | - | - | - | - |
| - | - | - | - | - | - | - | - | - | - | - |
| - | - | - | - | - | - | - | - | - | - | - |
| - | - | - | - | - | - | - | - | - | - | - |
| N | - | - | - | - | - | - | - | - | - | - |
| N | - | - | - | - | - | - | - | - | - | - |
| N | N | N | - | - | - | - | - | - | - | - |
| N | N | N | N | - | - | - | - | - | - | - |
| - | - | N | H | - | - | - | - | - | - | - |
| - | - | - | N | N | N | - | - | - | - | - |
| - | - | - | N | - | N | - | - | - | - | - |
| - | - | - | - | - | - | - | - | - | - | - |
| - | - | - | - | - | - | - | - | - | - | - |
| - | - | - | - | - | - | - | - | - | - | - |
| - | - | - | - | - | - | - | - | - | - | - |
| - | - | - | - | - | - | - | - | - | - | - |
| - | - | - | - | - | - | - | - | - | - | - |
| - | - | - | - | - | - | - | - | - | - | - |
| - | - | - | - | - | - | - | - | - | - | - |
| - | - | - | - | - | - | - | - | - | - | - |
| - | - | - | - | - | - | - | - | - | - | - |
| - | - | - | - | - | - | - | - | - | - | - |
| - | - | - | - | - | - | - | - | - | - | - |
| - | - | - | - | - | - | - | - | - | - | - |

| A:MET323 | A:ALA324 |
|---|---|
| - | - |
| - | - |
| - | - |
| - | - |
| N | - |
| - | - |
| - | N |
| - | - |
| - | - |
| - | - |
| N | - |
| N | - |
| N | - |
| - | - |
| - | - |
| - | - |
| - | - |
| - | - |
| - | - |
| - | - |
| - | - |
| - | - |
| - | - |
| - | - |
| - | - |
| - | - |
| - | - |
| - | - |
| - | - |
| - | - |
| - | - |
| - | - |
| - | - |
| - | - |
| - | - |
| - | - |
| - | - |
| - | - |
| - | - |
| - | - |
| - | - |
| - | - |
| - | - |
| - | - |
| - | - |

| | |
|---|---|
| - | - |
| - | - |
| - | - |
| - | - |
| - | - |
| - | - |
| - | - |
| - | - |
| - | - |
| - | - |
| - | - |
| - | - |
| - | - |
| - | - |
| - | - |
| - | - |
| - | - |
| - | - |
| - | - |
| - | - |
| - | - |
| - | - |
| - | - |
| - | - |

**Supplementary Table 2 N390 Mutation Energy**

| Mutation | Mutation Energy | Effect of Mutation | VDW Term | Electrostatic Term | Entropy Term |
|---|---|---|---|---|---|
| A:ASN390>ALA | 0 | NEUTRAL | 0 | 0 | 0 |
| A:ASN390>ARG | 0.17 | NEUTRAL | 0 | 0.34 | 0 |
| A:ASN390>ARGN | 0 | NEUTRAL | 0 | 0 | 0 |
| A:ASN390>ASP | -0.17 | NEUTRAL | 0 | -0.34 | 0 |
| A:ASN390>ASPH | 0 | NEUTRAL | 0 | 0 | 0 |
| A:ASN390>CYS | 0 | NEUTRAL | 0 | 0 | 0 |
| A:ASN390>GLN | 0.01 | NEUTRAL | 0 | 0.01 | 0 |
| A:ASN390>GLU | -0.17 | NEUTRAL | 0 | -0.33 | 0 |
| A:ASN390>GLUH | 0.01 | NEUTRAL | 0 | 0.01 | 0 |
| A:ASN390>GLY | 0 | NEUTRAL | 0 | 0 | 0 |
| A:ASN390>HIS | 0.01 | NEUTRAL | 0 | 0.01 | 0 |
| A:ASN390>HSC | 0.17 | NEUTRAL | 0 | 0.35 | 0 |
| A:ASN390>ILE | 0 | NEUTRAL | 0 | 0 | 0 |
| A:ASN390>LEU | 0 | NEUTRAL | 0 | 0 | 0 |
| A:ASN390>LYS | 0.17 | NEUTRAL | 0 | 0.34 | 0 |
| A:ASN390>LYSN | 0 | NEUTRAL | 0 | 0 | 0 |
| A:ASN390>MET | 0 | NEUTRAL | 0 | 0 | 0 |
| A:ASN390>PHE | 0 | NEUTRAL | 0 | 0 | 0 |
| A:ASN390>PRO | 0 | NEUTRAL | 0 | 0 | 0 |
| A:ASN390>SER | 0 | NEUTRAL | 0 | 0 | 0 |
| A:ASN390>THR | 0 | NEUTRAL | 0 | 0 | 0 |
| A:ASN390>TRP | 0 | NEUTRAL | 0 | 0 | 0 |
| A:ASN390>TYR | 0 | NEUTRAL | 0 | 0 | 0 |
| A:ASN390>VAL | 0 | NEUTRAL | 0 | 0 | 0 |

| Non-polar Term |
| ---: |
| 0 |
| 0 |
| 0 |
| 0 |
| 0 |
| 0 |
| 0 |
| 0 |
| 0 |
| 0 |
| 0 |
| 0 |
| 0 |
| 0 |
| 0 |
| 0 |
| 0 |
| 0 |
| 0 |
| 0 |
| 0 |
| 0 |
| 0 |
| 0 |

**Supplementary Table 3 G348 Mutation Energy**

| Mutation | Mutation Energy | Effect of Mutation | VDW Term | Electrostatic Term | Entropy Term |
|---|---|---|---|---|---|
| A:GLY348>ALA | 0 | NEUTRAL | 0 | 0 | 0 |
| A:GLY348>ARG | 0.13 | NEUTRAL | 0 | 0.26 | 0 |
| A:GLY348>ARGN | 0 | NEUTRAL | 0 | 0 | 0 |
| A:GLY348>ASN | 0.01 | NEUTRAL | 0 | 0.01 | 0 |
| A:GLY348>ASP | -0.14 | NEUTRAL | 0 | -0.27 | 0 |
| A:GLY348>ASPH | 0 | NEUTRAL | 0 | 0 | 0 |
| A:GLY348>CYS | 0 | NEUTRAL | 0 | 0 | 0 |
| A:GLY348>GLN | 0.01 | NEUTRAL | 0 | 0.01 | 0 |
| A:GLY348>GLU | -0.14 | NEUTRAL | 0 | -0.27 | 0 |
| A:GLY348>GLUH | 0 | NEUTRAL | 0 | 0 | 0 |
| A:GLY348>HIS | 0 | NEUTRAL | 0 | 0 | 0 |
| A:GLY348>HSC | 0.14 | NEUTRAL | 0 | 0.27 | 0 |
| A:GLY348>ILE | 0 | NEUTRAL | 0 | 0 | 0 |
| A:GLY348>LEU | -0.01 | NEUTRAL | 0 | -0.01 | 0 |
| A:GLY348>LYS | 0.13 | NEUTRAL | 0 | 0.26 | 0 |
| A:GLY348>LYSN | 0 | NEUTRAL | 0 | 0 | 0 |
| A:GLY348>MET | 0 | NEUTRAL | 0 | 0 | 0 |
| A:GLY348>PHE | 0 | NEUTRAL | 0 | 0 | 0 |
| A:GLY348>PRO | 0 | NEUTRAL | 0 | 0 | 0 |
| A:GLY348>SER | 0 | NEUTRAL | 0 | 0 | 0 |
| A:GLY348>THR | 0 | NEUTRAL | 0 | 0 | 0 |
| A:GLY348>TRP | 0 | NEUTRAL | 0 | 0 | 0 |
| A:GLY348>TYR | 0 | NEUTRAL | 0 | 0 | 0 |
| A:GLY348>VAL | 0 | NEUTRAL | 0 | 0 | 0 |

| Non-polar Term |
| --- |
| 0 |
| 0 |
| 0 |
| 0 |
| 0 |
| 0 |
| 0 |
| 0 |
| 0 |
| 0 |
| 0 |
| 0 |
| 0 |
| 0 |
| 0 |
| 0 |
| 0 |
| 0 |
| 0 |
| 0 |
| 0 |
| 0 |
| 0 |
| 0 |

**Supplementary Table 4 D385 Mutation Energy**

| Mutation | Mutation Energy | Effect of Mutatior | VDW Term | Electrostatic Term | Entropy Term |
|---|---|---|---|---|---|
| B:ASP385>ALA | 0.05 | NEUTRAL | 0.29 | -0.18 | 0 |
| B:ASP385>ARG | -2.87 | STABILIZING | -5.46 | -0.24 | -2.00E-02 |
| B:ASP385>ARGN | -2.9 | STABILIZING | -5.42 | -0.31 | -4.00E-02 |
| B:ASP385>ASN | -1.87 | STABILIZING | -3.53 | -0.2 | -1.00E-02 |
| B:ASP385>ASP | -0.2 | NEUTRAL | -0.4 | 0 | 0 |
| B:ASP385>ASPH | -2.33 | STABILIZING | -4.44 | -0.18 | -2.00E-02 |
| B:ASP385>CYS | 0.02 | NEUTRAL | 0.24 | -0.19 | 0 |
| B:ASP385>GLN | -2.21 | STABILIZING | -4.2 | -0.18 | -2.00E-02 |
| B:ASP385>GLU | -2.04 | STABILIZING | -4.05 | -0.01 | -1.00E-02 |
| B:ASP385>GLUH | -2.04 | STABILIZING | -3.78 | -0.23 | -5.00E-02 |
| B:ASP385>GLY | 0.09 | NEUTRAL | 0.36 | -0.18 | 0 |
| B:ASP385>HIS | -2.34 | STABILIZING | -4.53 | -0.18 | 2.00E-02 |
| B:ASP385>HSC | -2.35 | STABILIZING | -4.71 | -0.01 | 1.00E-02 |
| B:ASP385>ILE | -2.26 | STABILIZING | -4.25 | -0.23 | -2.00E-02 |
| B:ASP385>LEU | -2.19 | STABILIZING | -4.11 | -0.21 | -4.00E-02 |
| B:ASP385>LYS | -2.31 | STABILIZING | -4.45 | -0.11 | -4.00E-02 |
| B:ASP385>LYSN | -2.34 | STABILIZING | -4.36 | -0.24 | -5.00E-02 |
| B:ASP385>MET | -1.72 | STABILIZING | -3.12 | -0.23 | -6.00E-02 |
| B:ASP385>PHE | -2.49 | STABILIZING | -4.78 | -0.22 | 1.00E-02 |
| B:ASP385>PRO | -2.31 | STABILIZING | -4.38 | -0.23 | -1.00E-02 |
| B:ASP385>SER | 0.03 | NEUTRAL | 0.24 | -0.18 | 0 |
| B:ASP385>THR | -0.96 | STABILIZING | -1.71 | -0.19 | -1.00E-02 |
| B:ASP385>TRP | -2.94 | STABILIZING | -5.51 | -0.21 | -0.1 |
| B:ASP385>TYR | -2.88 | STABILIZING | -5.51 | -0.21 | -2.00E-02 |
| B:ASP385>VAL | -1.9 | STABILIZING | -3.58 | -0.21 | -1.00E-02 |

| Non-polar Term |
| --- |
| 0 |
| 0 |
| 0 |
| 0 |
| 0 |
| 0 |
| 0 |
| 0 |
| 0 |
| 0 |
| 0 |
| 0 |
| 0 |
| 0 |
| 0 |
| 0 |
| 0 |
| 0 |
| 0 |
| 0 |
| 0 |
| 0 |
| 0 |
| 0 |
| 0 |

**Supplementary Table 5 D257 Mutation Energy**

| Mutation | Mutation Energy | Effect of Mutation | VDW Term | Electrostatic Term | Entropy Term |
|---|---|---|---|---|---|
| B:ASP257>ALA | 0.08 | NEUTRAL | 0.15 | -0.11 | 8.00E-02 |
| B:ASP257>ARG | -2.36 | STABILIZING | -4.69 | -0.11 | 5.00E-02 |
| B:ASP257>ARGN | 0.1 | NEUTRAL | 6.00E-02 | 0.02 | 7.00E-02 |
| B:ASP257>ASN | -0.04 | NEUTRAL | 0 | -0.09 | 0 |
| B:ASP257>ASP | -0.02 | NEUTRAL | 1.00E-02 | 0 | -3.00E-02 |
| B:ASP257>ASPH | -0.04 | NEUTRAL | 1.00E-02 | -0.09 | 0 |
| B:ASP257>CYS | 0.04 | NEUTRAL | 0.11 | -0.11 | 5.00E-02 |
| B:ASP257>GLN | -0.04 | NEUTRAL | -2.00E-02 | -0.07 | 1.00E-02 |
| B:ASP257>GLU | -0.03 | NEUTRAL | -0.13 | 0.05 | 1.00E-02 |
| B:ASP257>GLUH | -0.06 | NEUTRAL | -0.2 | -0.08 | 0.1 |
| B:ASP257>GLY | 0.11 | NEUTRAL | 0.19 | -0.11 | 9.00E-02 |
| B:ASP257>HIS | -0.28 | NEUTRAL | -0.35 | -0.08 | -8.00E-02 |
| B:ASP257>HSC | -0.2 | NEUTRAL | -0.32 | 0.07 | -9.00E-02 |
| B:ASP257>ILE | -0.11 | NEUTRAL | -9.00E-02 | -0.11 | -1.00E-02 |
| B:ASP257>LEU | -0.17 | NEUTRAL | -0.19 | -0.1 | -3.00E-02 |
| B:ASP257>LYS | -2.12 | STABILIZING | -4.18 | -0.02 | -2.00E-02 |
| B:ASP257>LYSN | -1.98 | STABILIZING | -3.82 | -0.1 | -2.00E-02 |
| B:ASP257>MET | -1.1 | STABILIZING | -2.07 | -0.11 | -1.00E-02 |
| B:ASP257>PHE | -0.49 | NEUTRAL | -0.76 | -0.1 | -7.00E-02 |
| B:ASP257>PRO | 0.03 | NEUTRAL | 7.00E-02 | -0.1 | 6.00E-02 |
| B:ASP257>SER | 0.08 | NEUTRAL | 0.15 | -0.11 | 8.00E-02 |
| B:ASP257>THR | 0.02 | NEUTRAL | 6.00E-02 | -0.1 | 5.00E-02 |
| B:ASP257>TRP | -0.46 | NEUTRAL | -0.62 | -0.1 | -0.13 |
| B:ASP257>TYR | -1.97 | STABILIZING | -3.64 | -0.18 | -8.00E-02 |
| B:ASP257>VAL | -0.01 | NEUTRAL | 3.00E-02 | -0.11 | 4.00E-02 |

| Non-polar Term |
| --- |
| 0 |
| 0 |
| 0 |
| 0 |
| 0 |
| 0 |
| 0 |
| 0 |
| 0 |
| 0 |
| 0 |
| 0 |
| 0 |
| 0 |
| 0 |
| 0 |
| 0 |
| 0 |
| 0 |
| 0 |
| 0 |
| 0 |
| 0 |
| 0 |
| 0 |

**Supplementary Table 6 A315 Mutation Energy**

| Mutation | Mutation Energy | Effect of Mutation | VDW Term | Electrostatic Term | Entropy Term |
|---|---|---|---|---|---|
| A:ALA315>ARG | -4.67 | STABILIZING | -14.47 | 1.4 | 2.33 |
| A:ALA315>ARGN | -4.18 | STABILIZING | -14.51 | 2.71 | 2.15 |
| A:ALA315>ASN | -2.65 | STABILIZING | -7.25 | 0.68 | 0.79 |
| A:ALA315>ASP | -2.42 | STABILIZING | -6.75 | 0.92 | 0.62 |
| A:ALA315>ASPH | -3.21 | STABILIZING | -7.82 | 0.4 | 0.62 |
| A:ALA315>CYS | -2.99 | STABILIZING | -6.2 | 0.08 | 9.00E-02 |
| A:ALA315>GLN | -3.02 | STABILIZING | -9.87 | 0.87 | 1.85 |
| A:ALA315>GLU | -2.33 | STABILIZING | -10.44 | 3.16 | 1.64 |
| A:ALA315>GLUH | -3.28 | STABILIZING | -9.65 | 0.42 | 1.67 |
| A:ALA315>GLY | -2.1 | STABILIZING | -4.37 | 0.04 | 8.00E-02 |
| A:ALA315>HIS | -3.67 | STABILIZING | -9.56 | 0.64 | 0.99 |
| A:ALA315>HSC | -3.69 | STABILIZING | -10.35 | 1.34 | 1.02 |
| A:ALA315>ILE | -4.49 | STABILIZING | -10.38 | 0.13 | 0.79 |
| A:ALA315>LEU | -4.34 | STABILIZING | -10.27 | 0.19 | 0.88 |
| A:ALA315>LYS | -4.14 | STABILIZING | -12.89 | 1.12 | 2.18 |
| A:ALA315>LYSN | -4.58 | STABILIZING | -13.3 | 0.58 | 2.22 |
| A:ALA315>MET | -3.27 | STABILIZING | -9 | 0.22 | 1.4 |
| A:ALA315>PHE | -5.3 | STABILIZING | -12.01 | 0.35 | 0.66 |
| A:ALA315>PRO | -3.54 | STABILIZING | -7.15 | -0.1 | 0.1 |
| A:ALA315>SER | -1.98 | STABILIZING | -5.79 | 0.13 | 1.06 |
| A:ALA315>THR | -2.88 | STABILIZING | -7.53 | 0.09 | 1.05 |
| A:ALA315>TRP | -6.99 | STABILIZING | -16.05 | 0.62 | 0.91 |
| A:ALA315>TYR | -5.61 | STABILIZING | -13.63 | 0.85 | 0.97 |
| A:ALA315>VAL | -3.92 | STABILIZING | -8.76 | 0.03 | 0.56 |

| Non-polar Term |
| ---: |
| 0 |
| 0 |
| 0 |
| 0 |
| 0 |
| 0 |
| 0 |
| 0 |
| 0 |
| 0 |
| 0 |
| 0 |
| 0 |
| 0 |
| 0 |
| 0 |
| 0 |
| 0 |
| 0 |
| 0 |
| 0 |
| 0 |
| 0 |
| 0 |

F1B INPUT lysate Myc

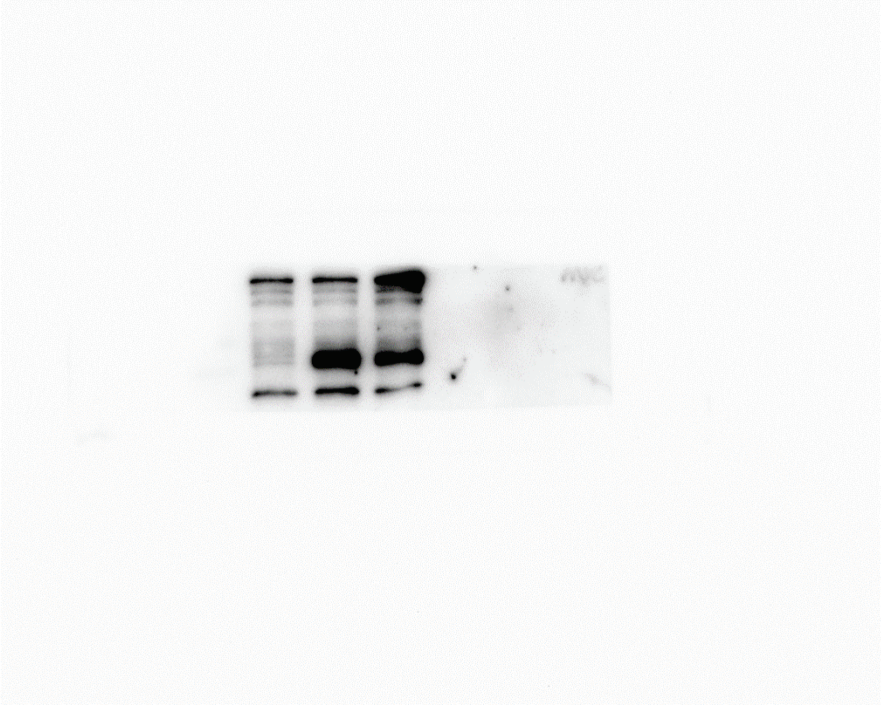

F1B Myc

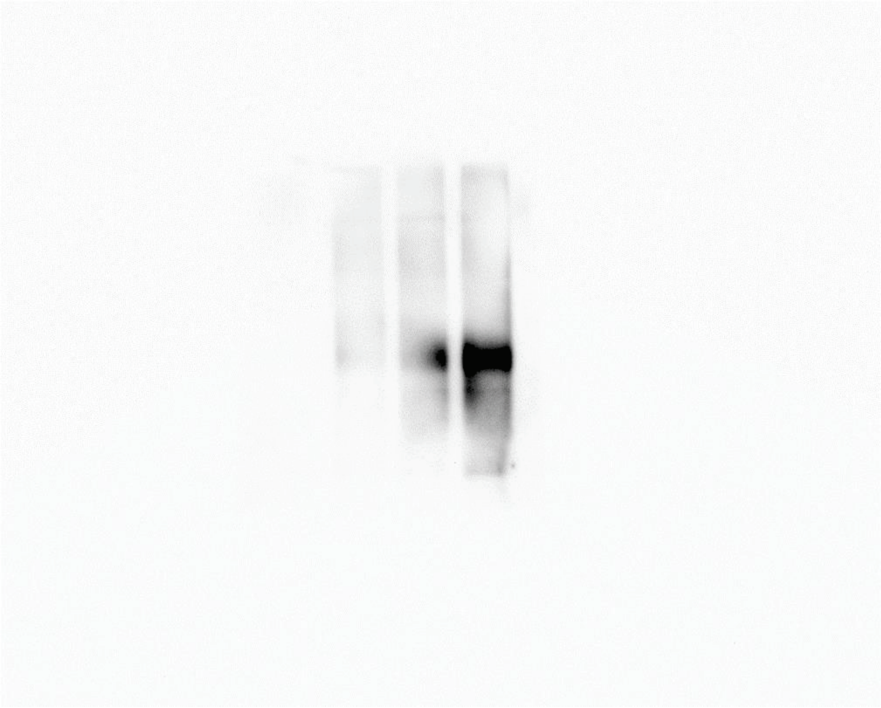

F1B PS1

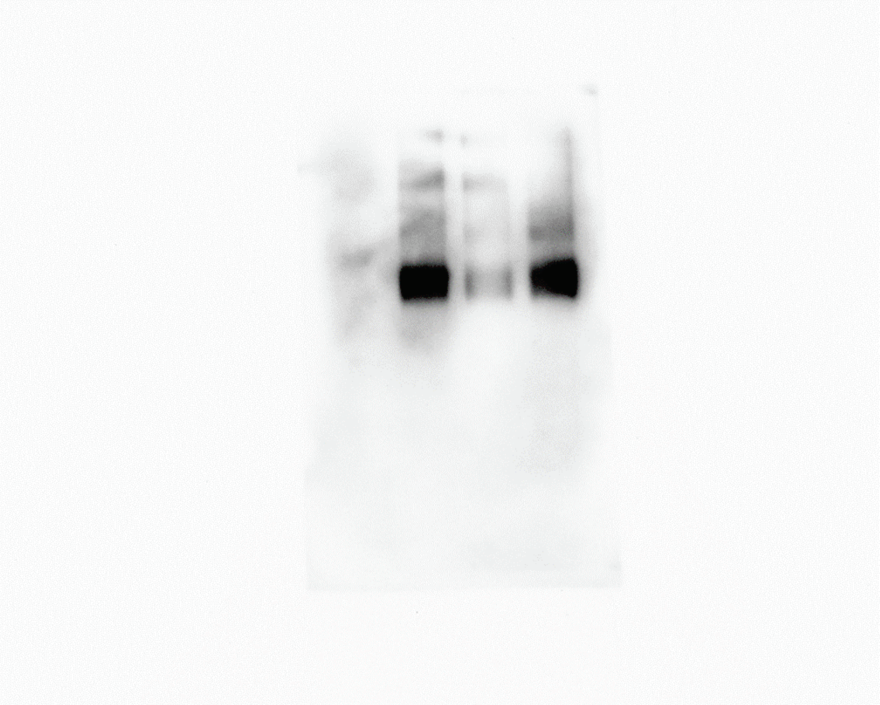

F1B INPUT lysate PS1

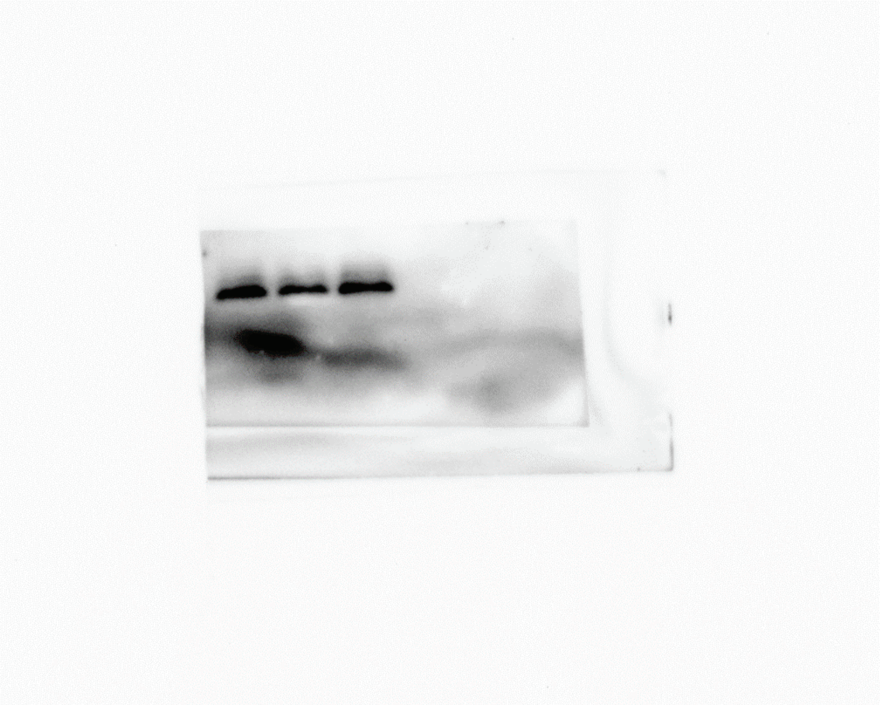

F1E H3

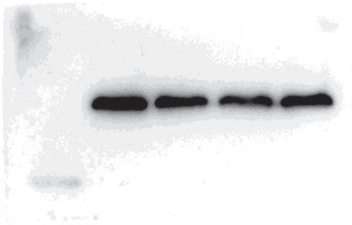

F1E GAPDH

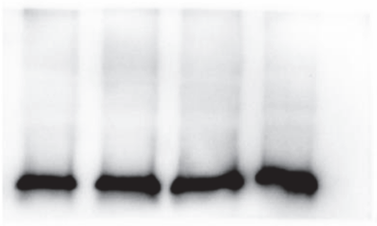

F1E TDP43

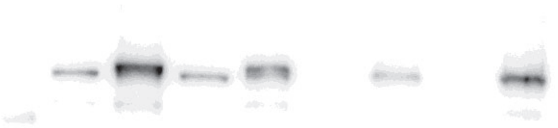

F1E MYC

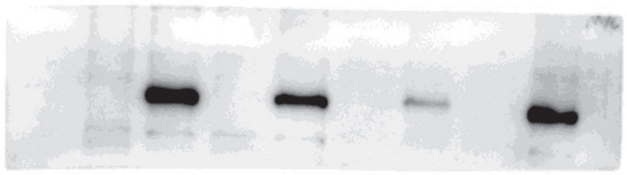

F2A GAPDH

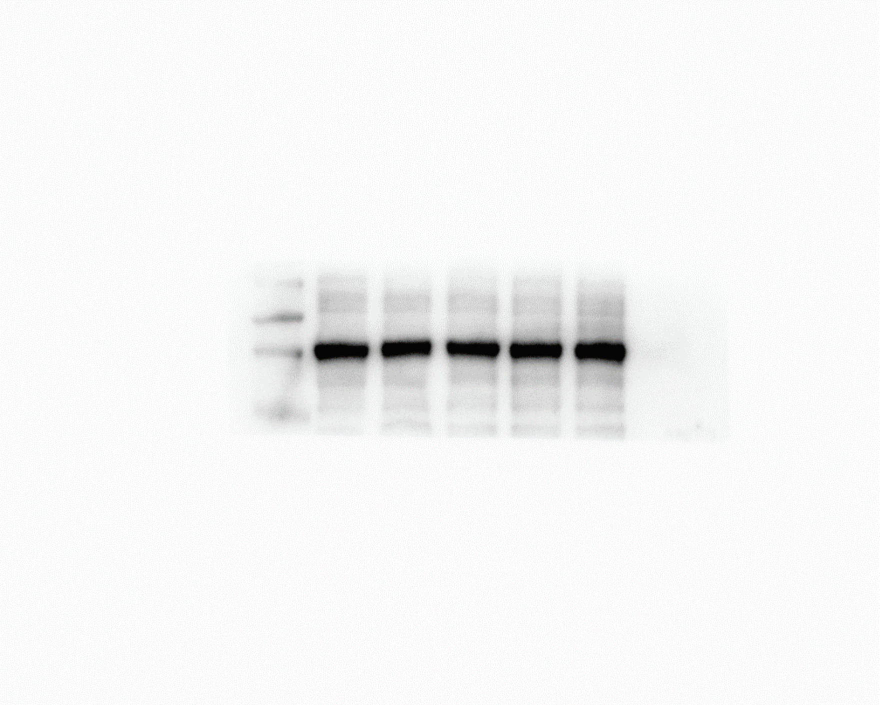

F2A TDP43

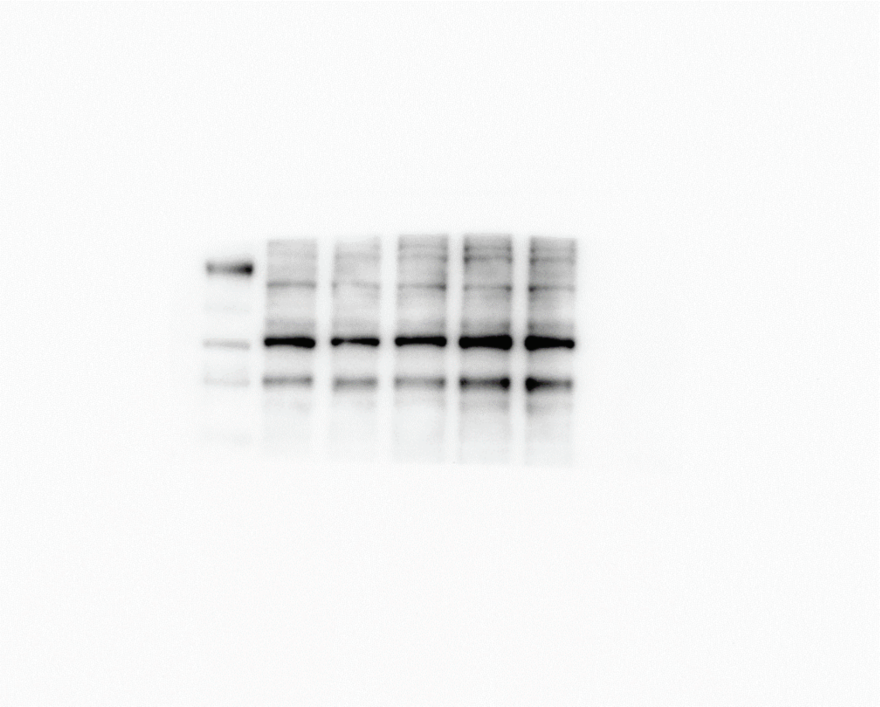

F2C GAPDH

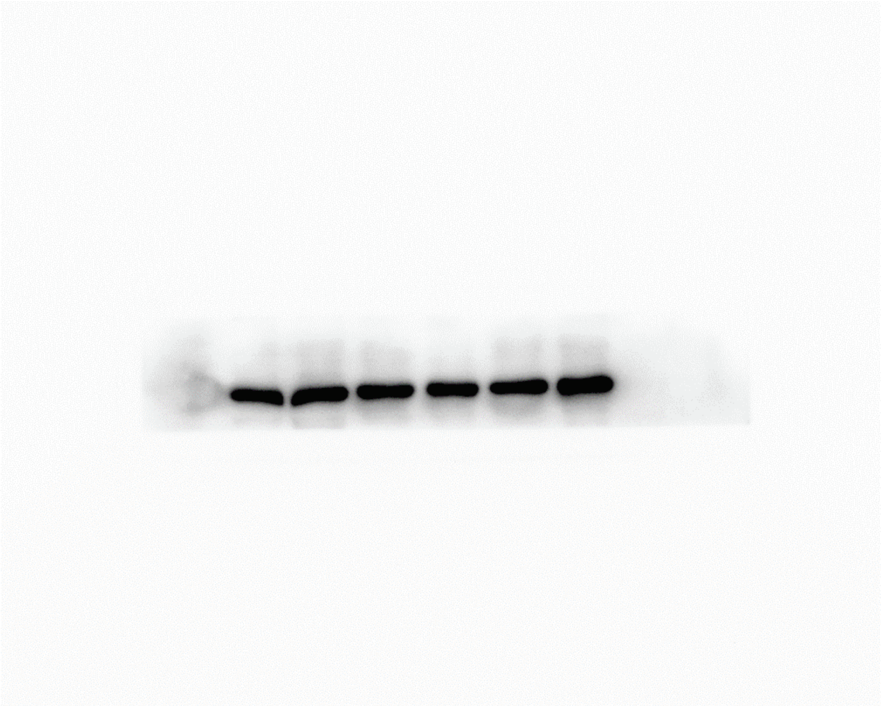

F2C PS1

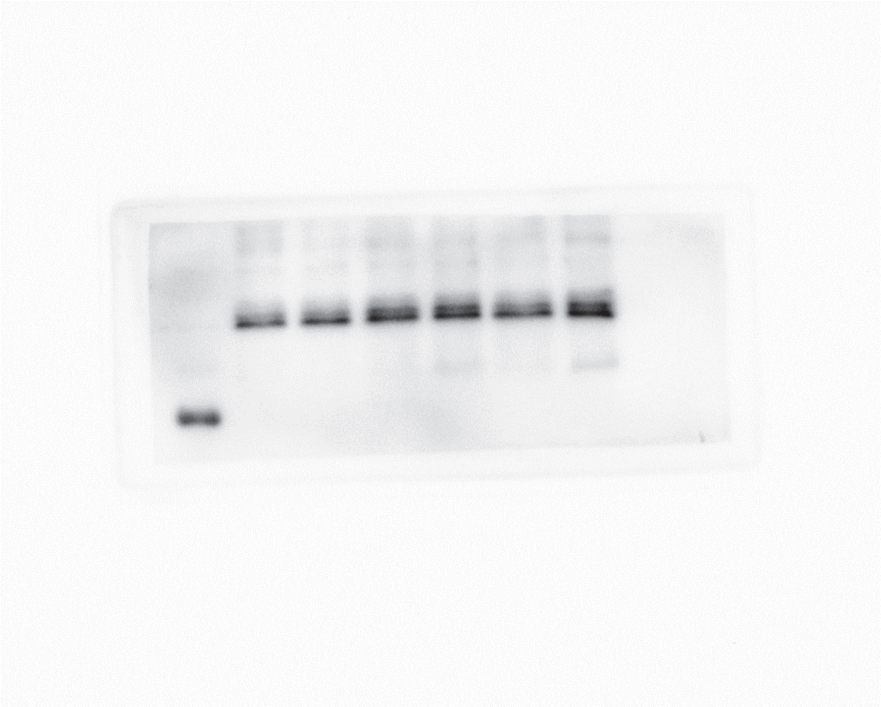

F2C TDP43

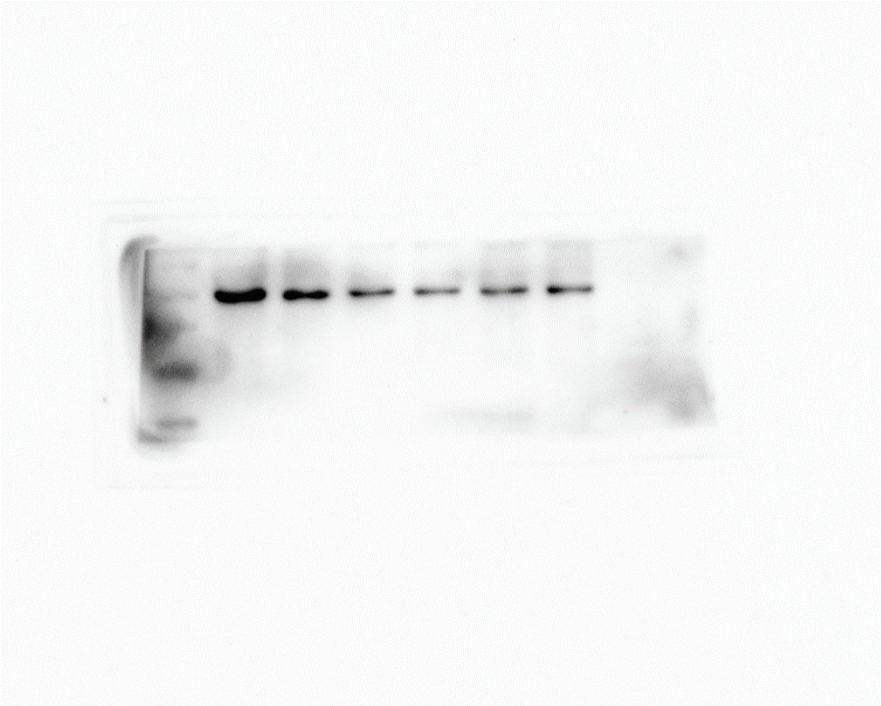

F3A HELA TDP43

Long exposure

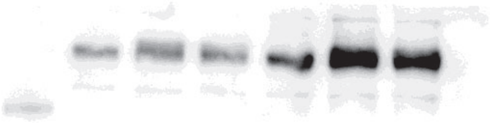

Short exposure

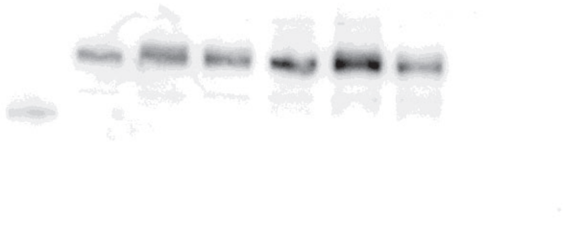

F3A HELA MYC

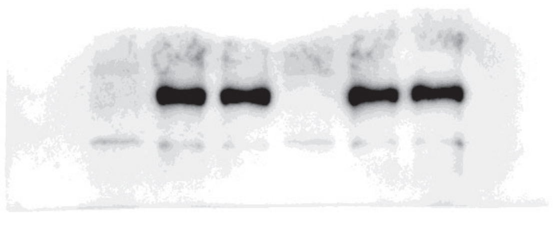

F3A HELA APP

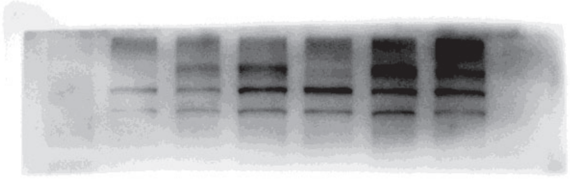

F3A HELA PS1

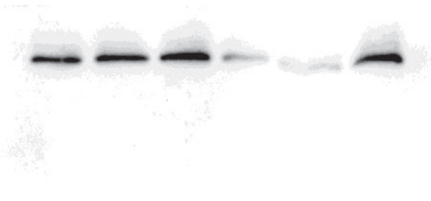

F3A HELA GAPDH

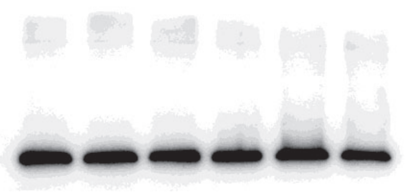

F3C NSC34 TDP43

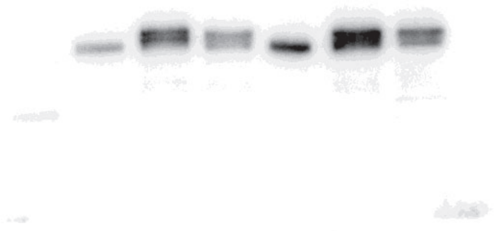

F3C NSC34 MYC

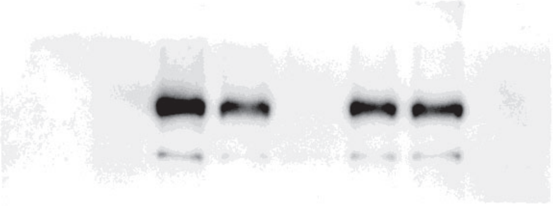

F3C NSC34 APP

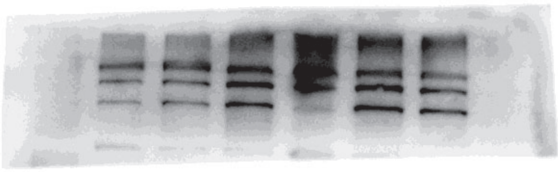

F3C NSC34 PS1

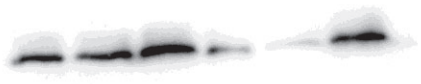

F3C NSC34 GAPDH

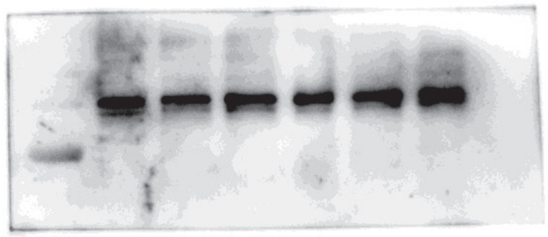

F3E MEF TDP43

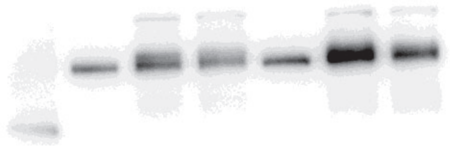

F3E MEF MYC

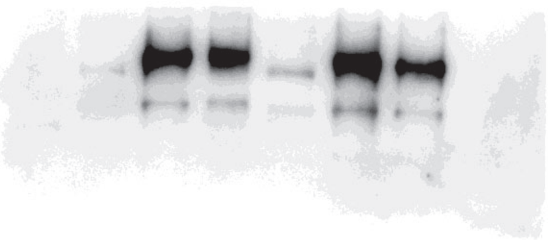

F3E MEF GAPDH

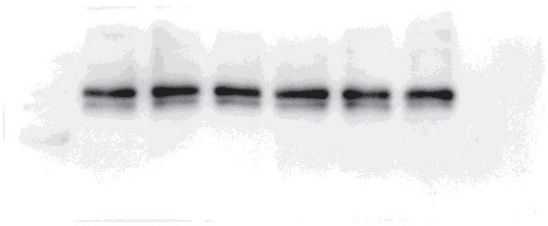

F3E MEF APP

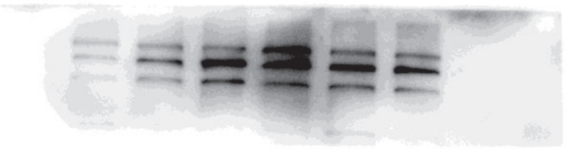

F3G GAPDH

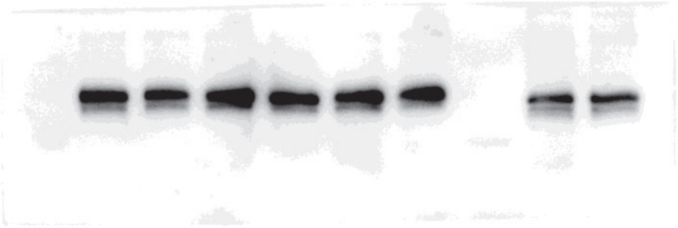

F3G TDP43

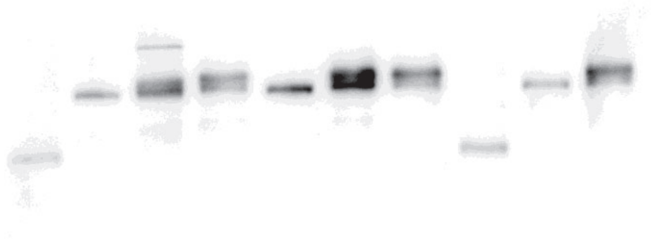

F3G MYC

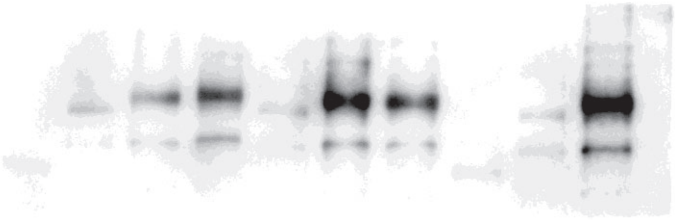

F3G APP

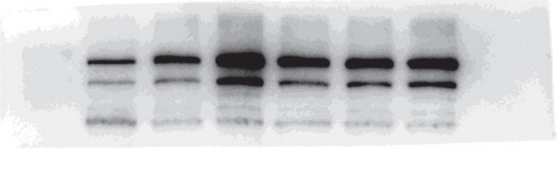

F4A HELA APP

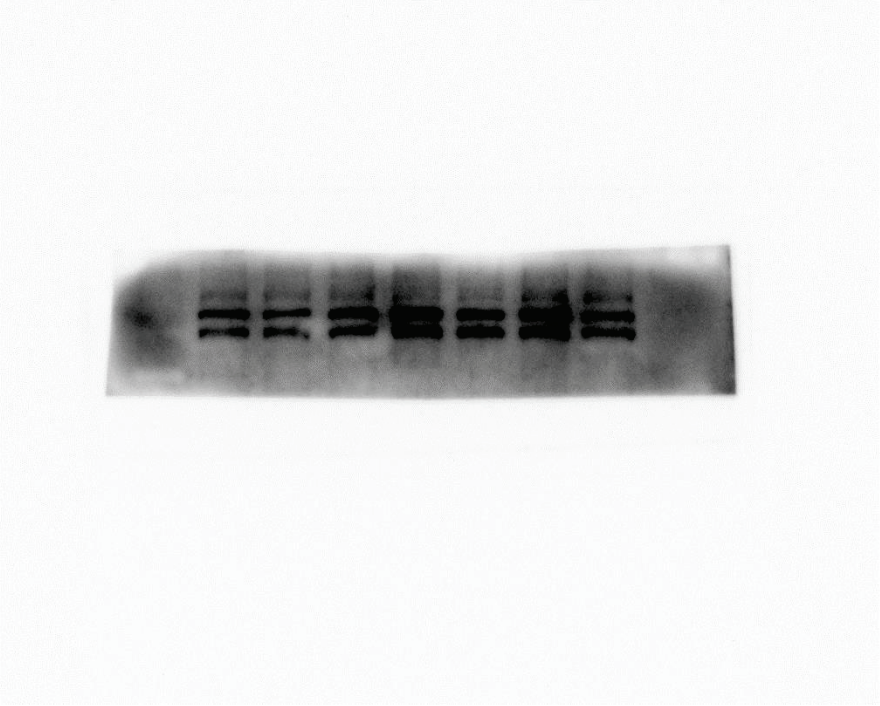

F4A HELA E-cadherin

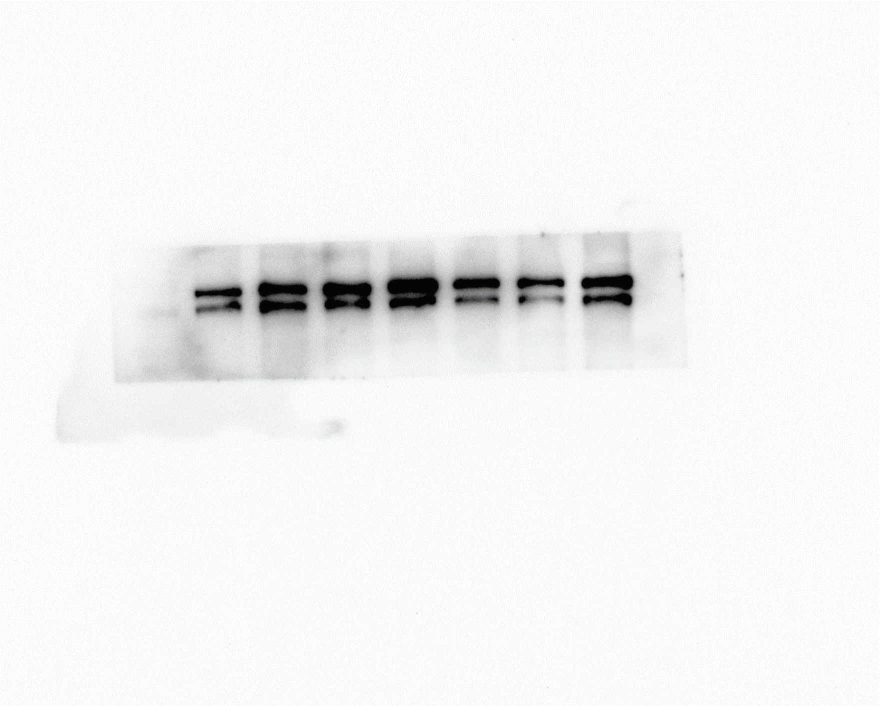

F4A HELA GAPDH

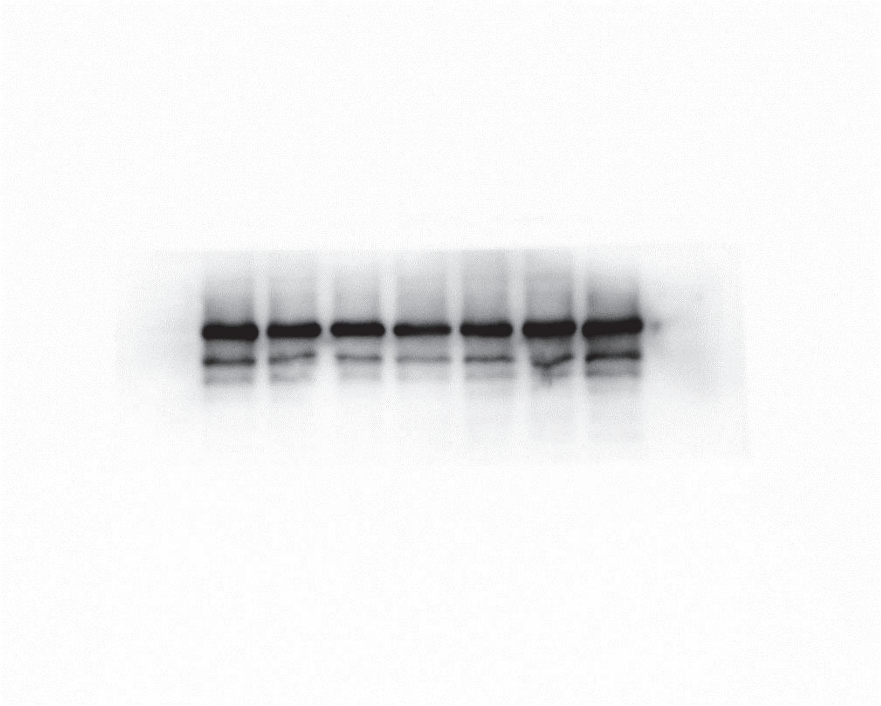

F4A HELA MYC

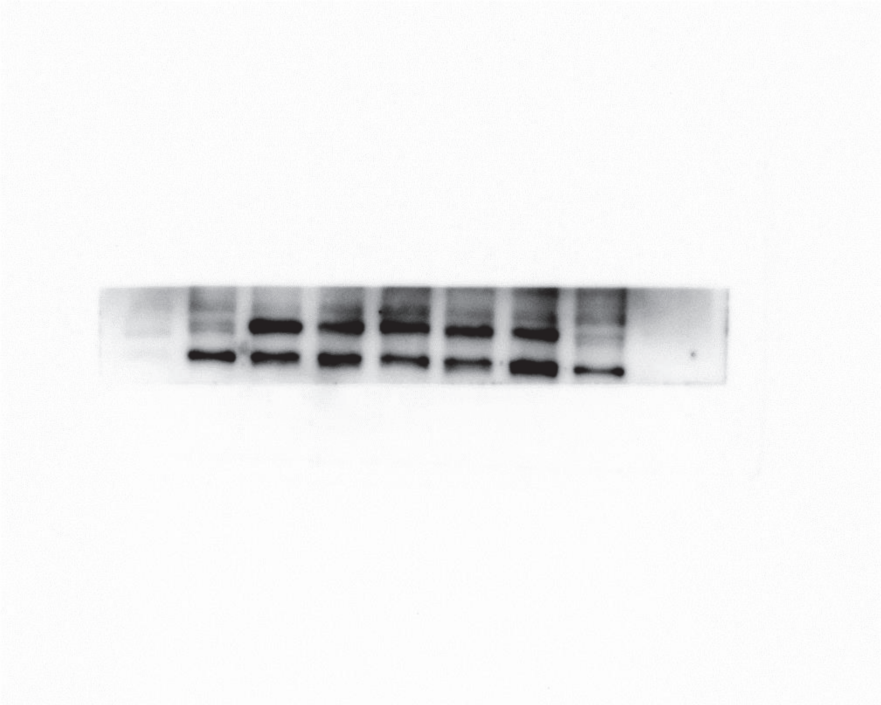

F4A HELA PS1 13KD



F4A HELA PS1

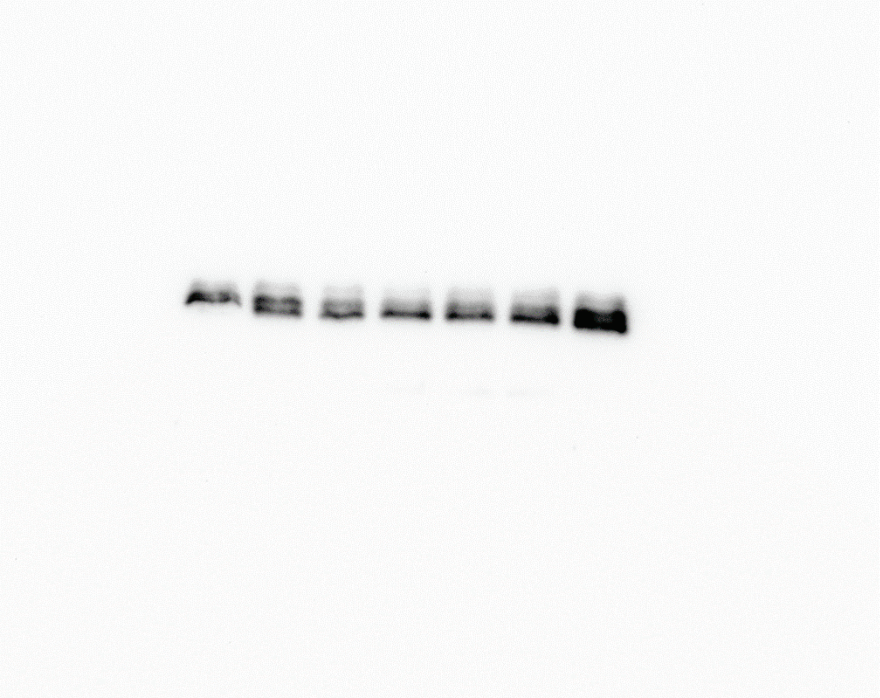

F4A HELA TDP43

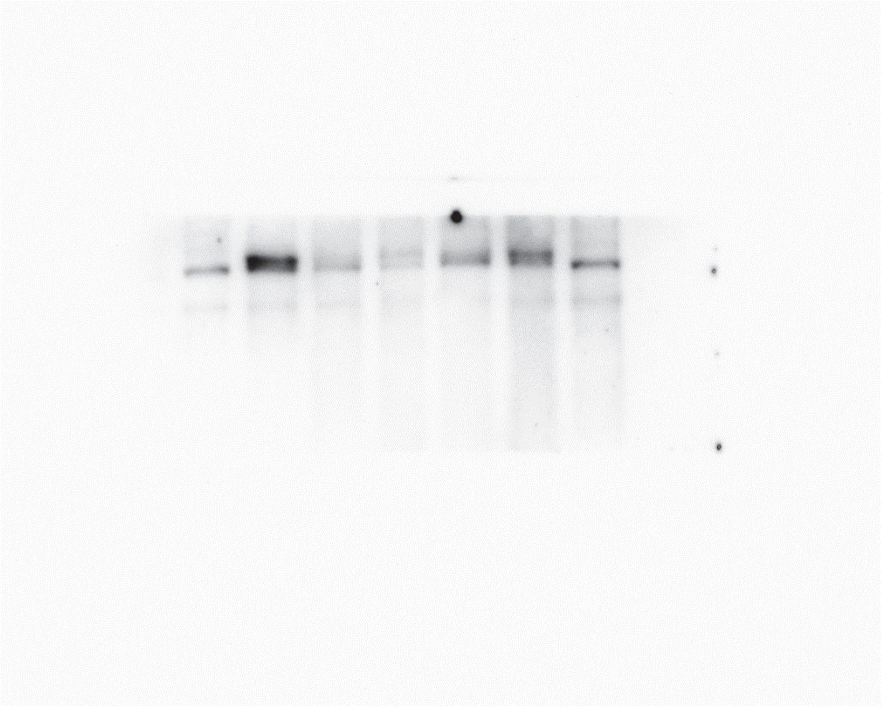

F4C NSC34 E-cadherin

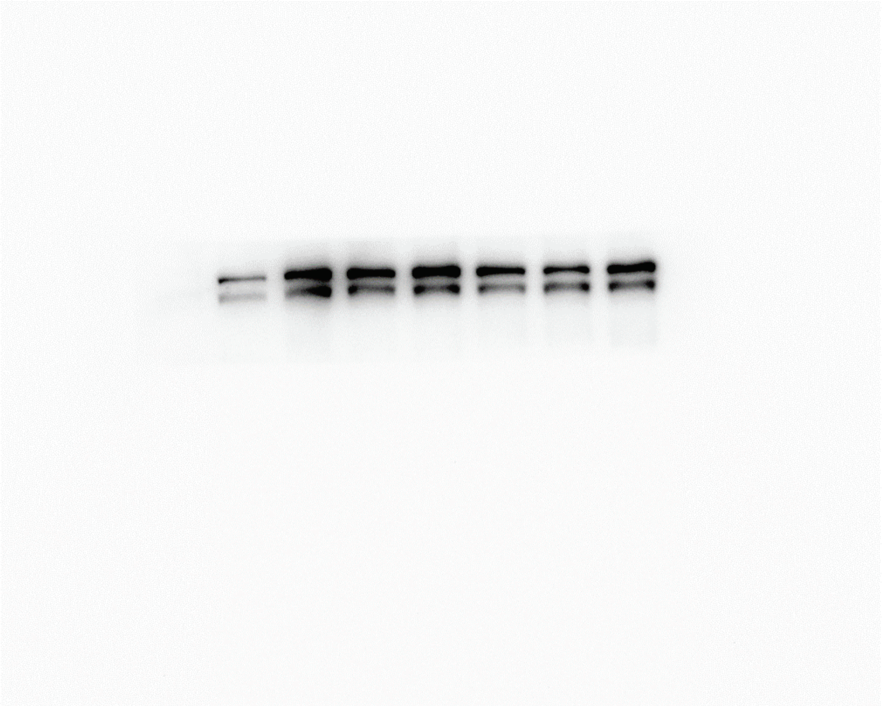

F4C NSC34 GAPDH

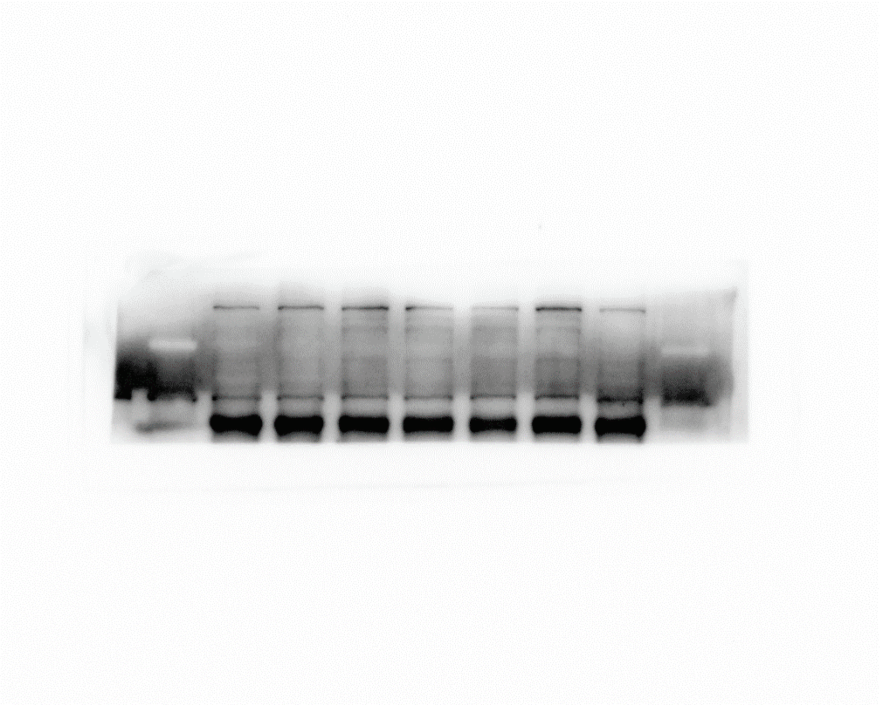

F4C NSC34 APP

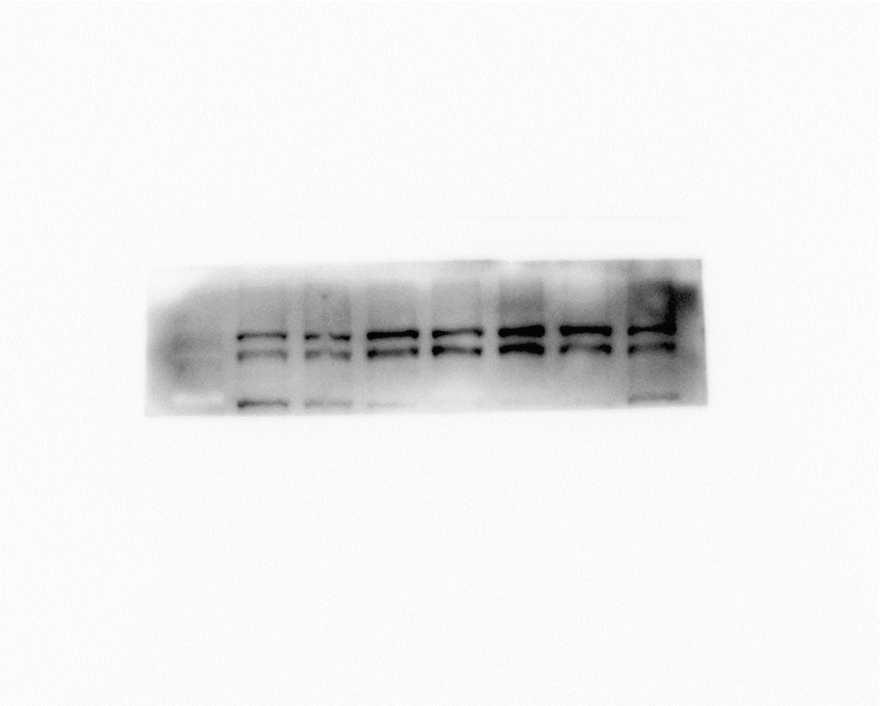

F4C NSC34 MYC

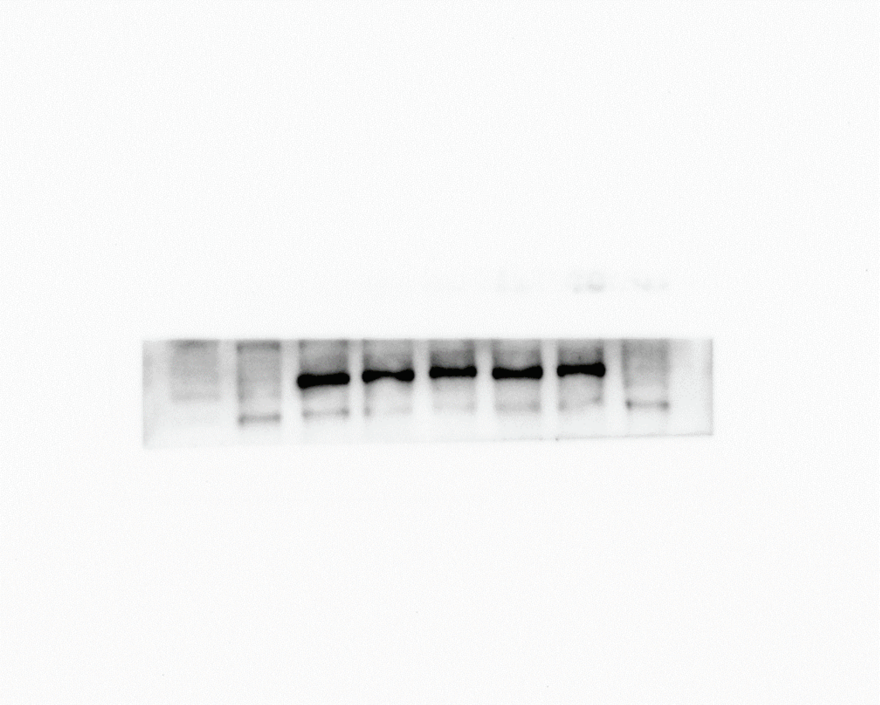

F4C NSC34 PS1

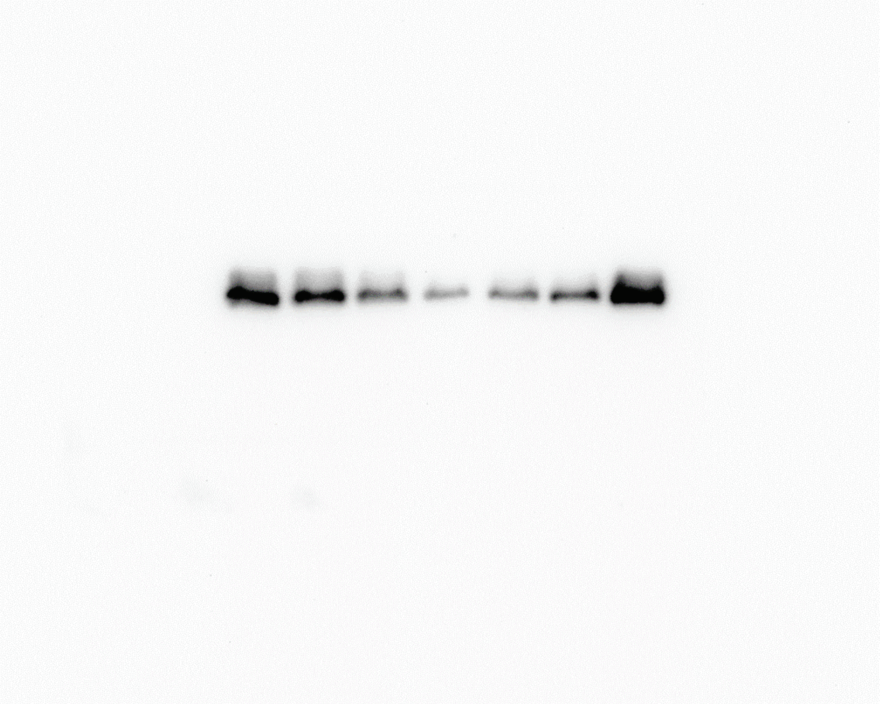

F4C NSC34 TDP43

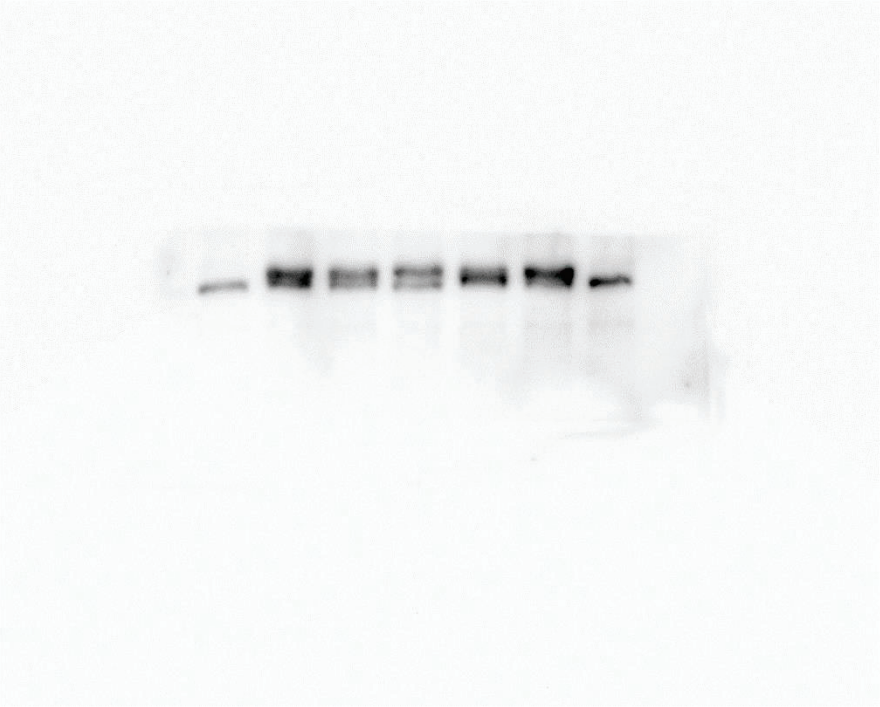

F4G NSC34 GAPDH

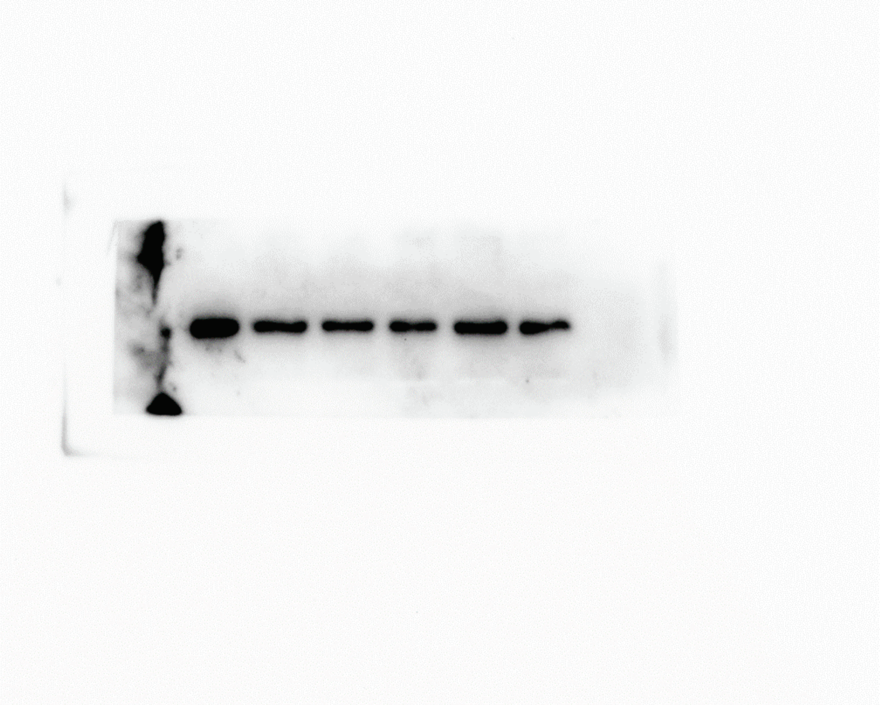

F4G NSC34 MYC-NOTCH

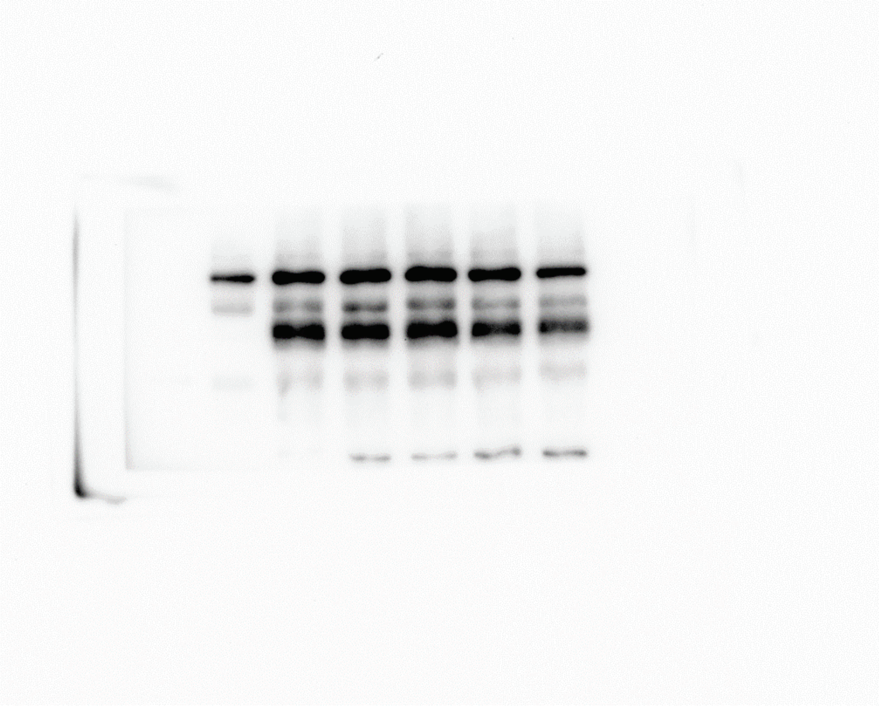

F4G NSC34 MYC-TDP43

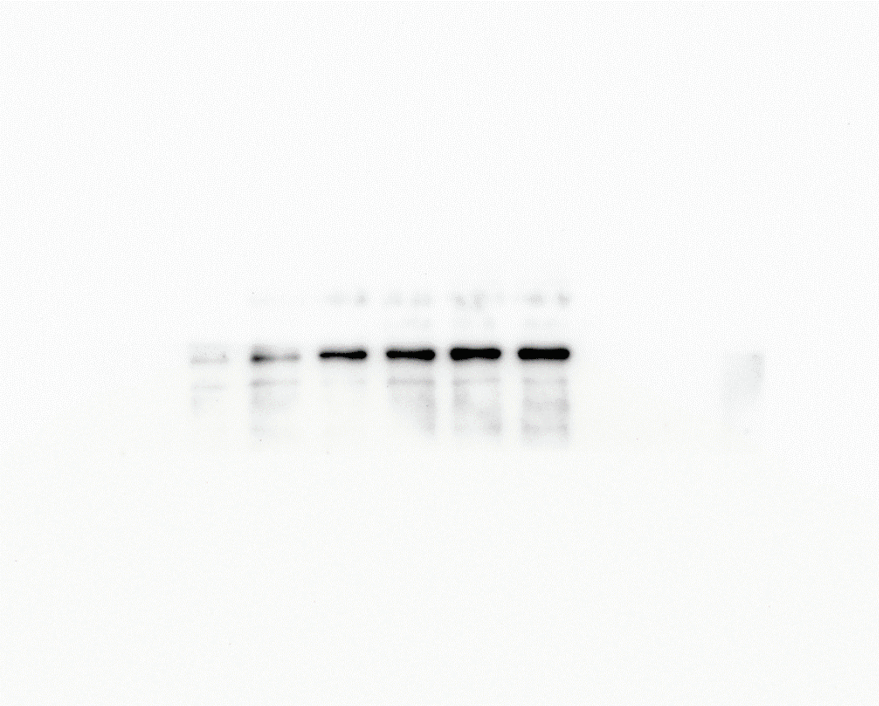

F4G NSC34 TDP43

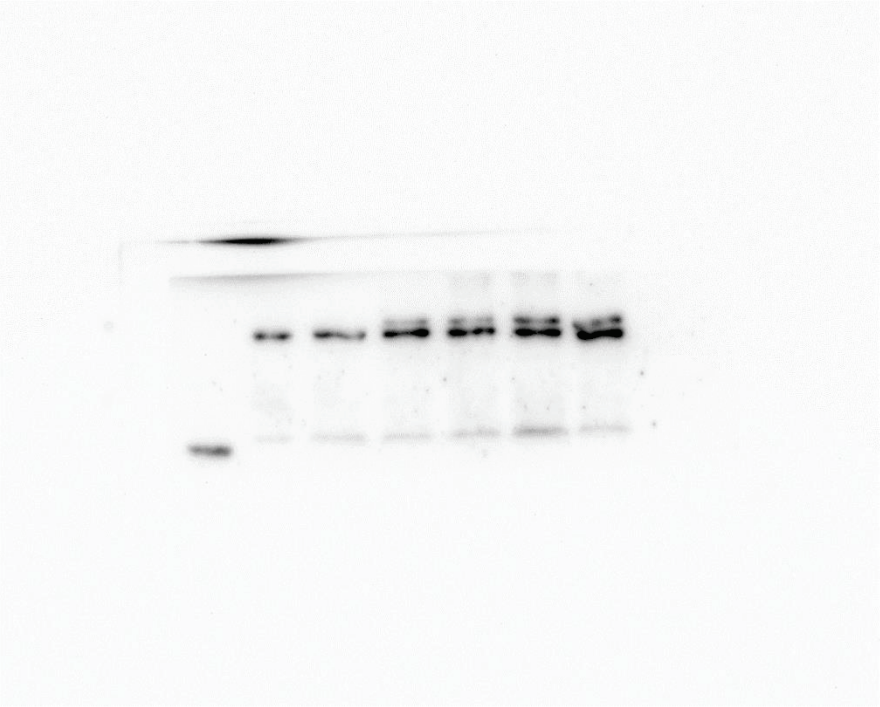

F5A HELA APP

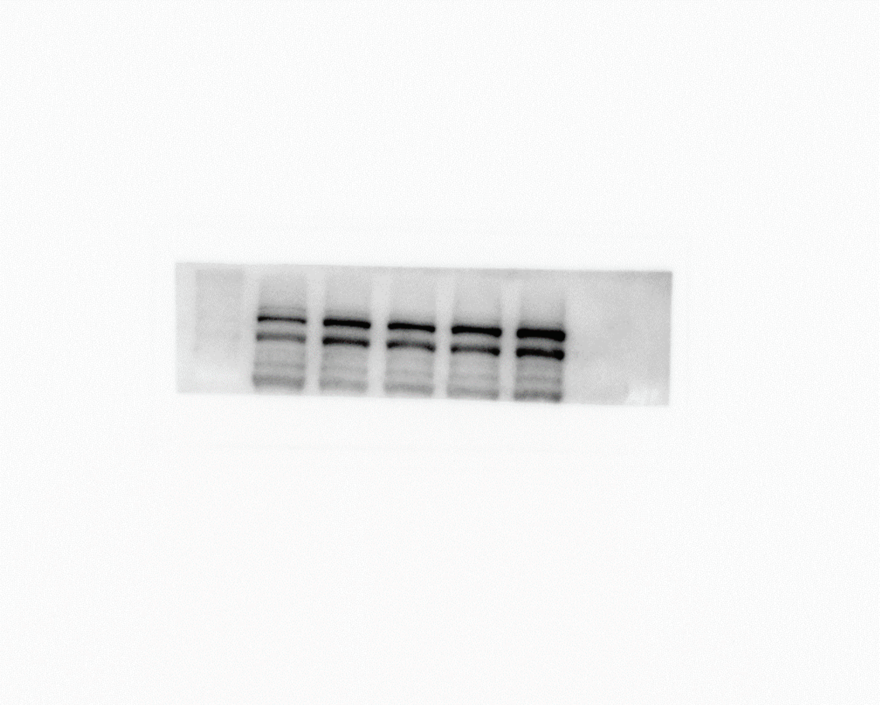

F5A HELA E-cadherin

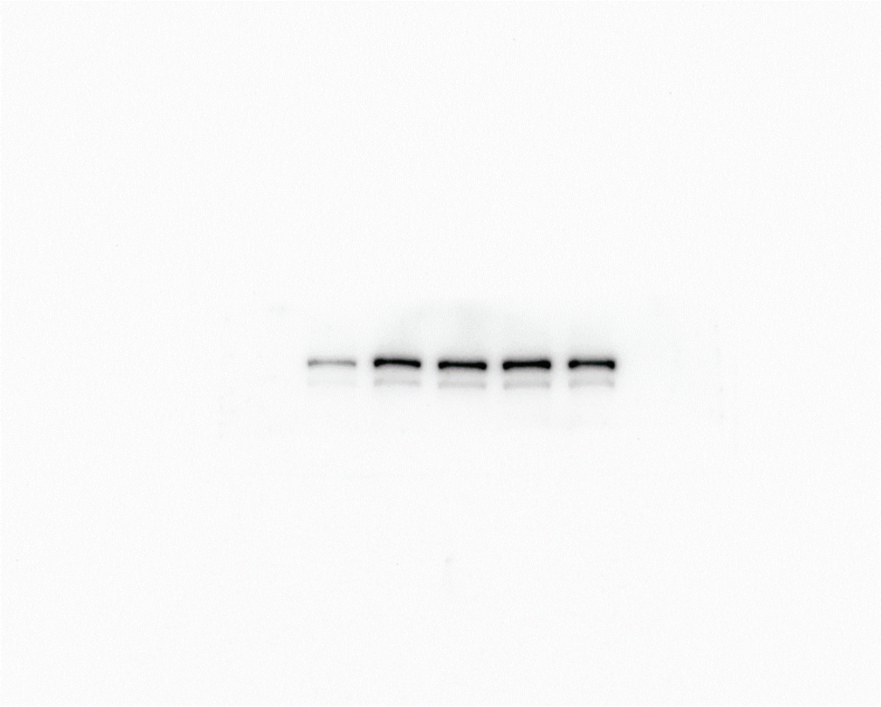

F5A HELA GAPDH

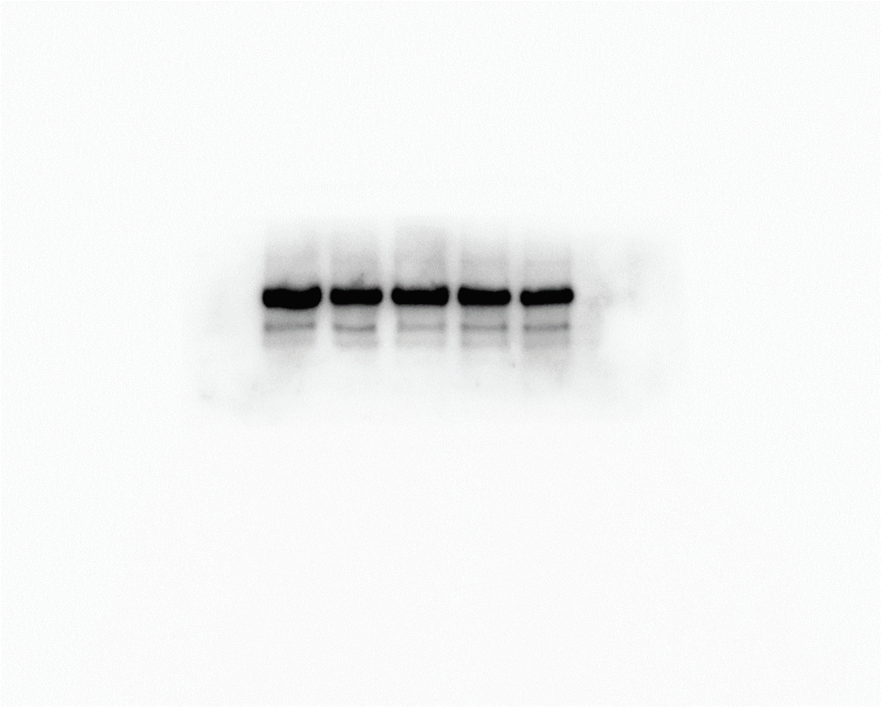

F5A HELA MYC

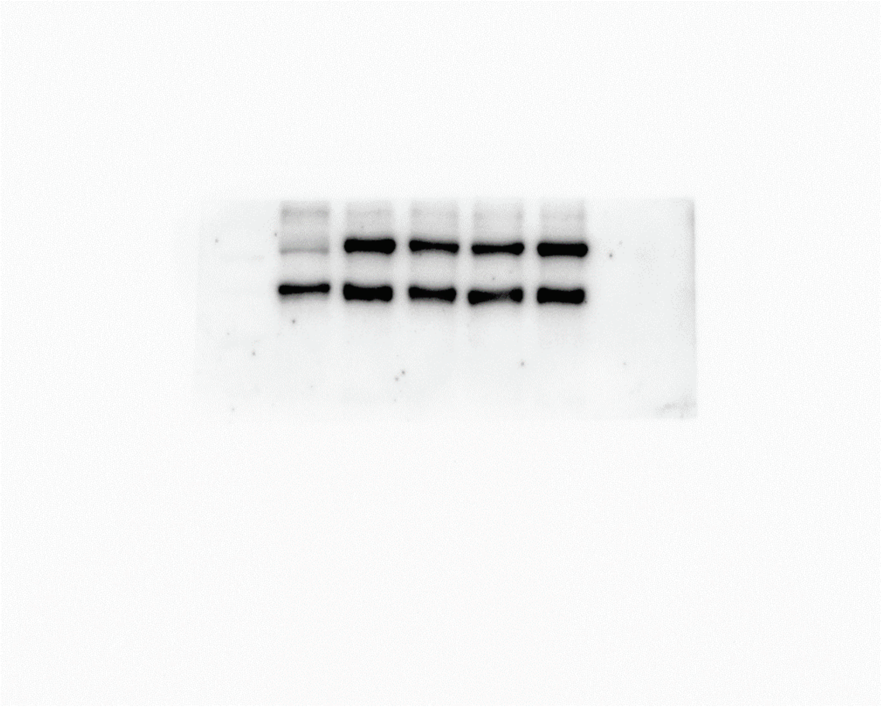

F5A HELA TDP43

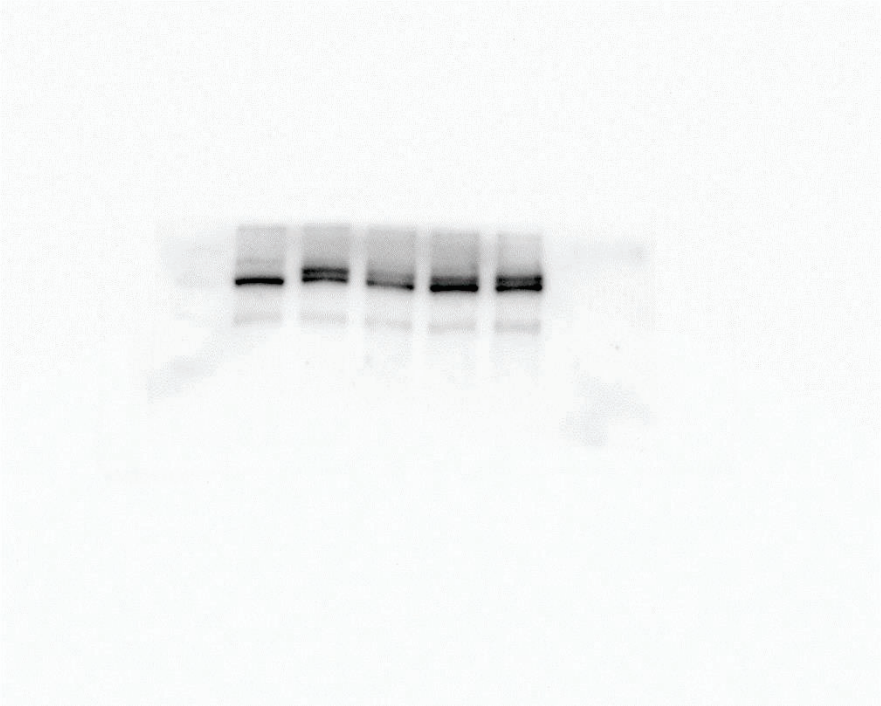

F5C NSC34 APP

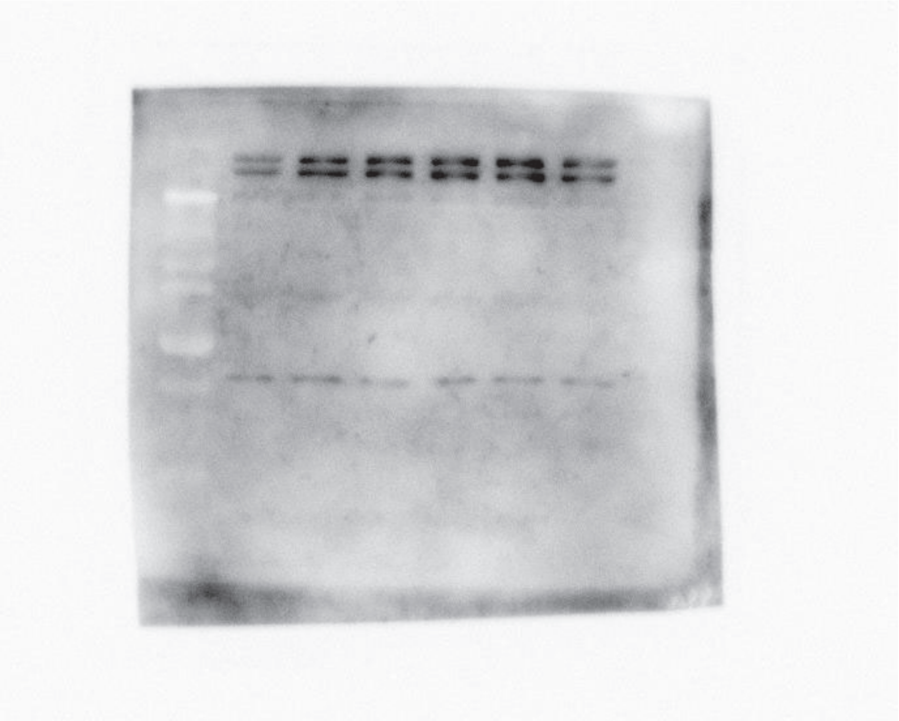

F5C NSC34 E-cadherin

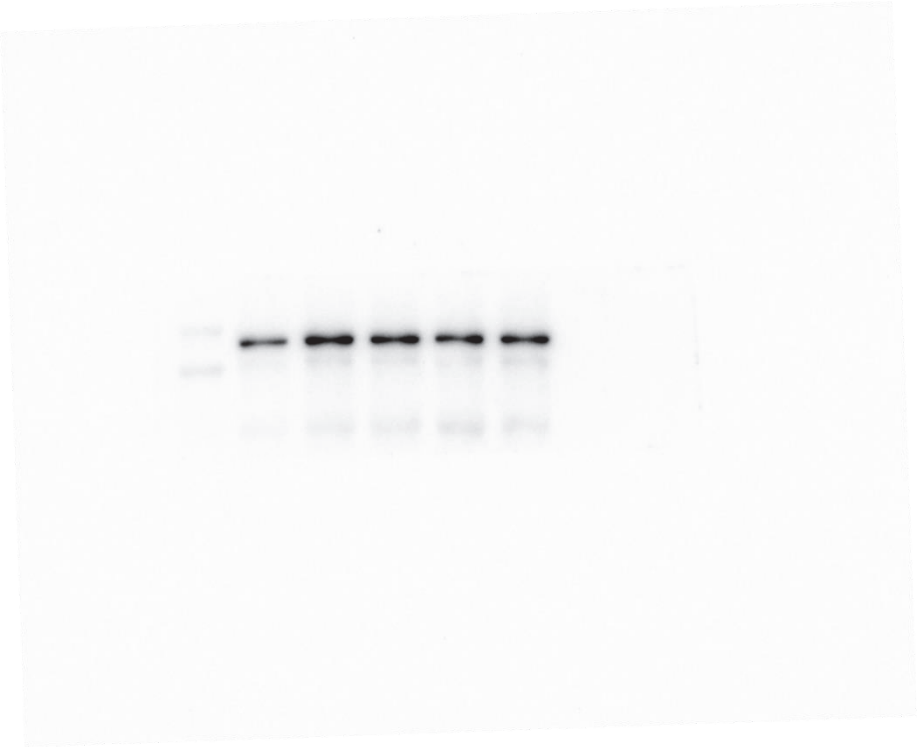

F5C NSC34 GAPDH

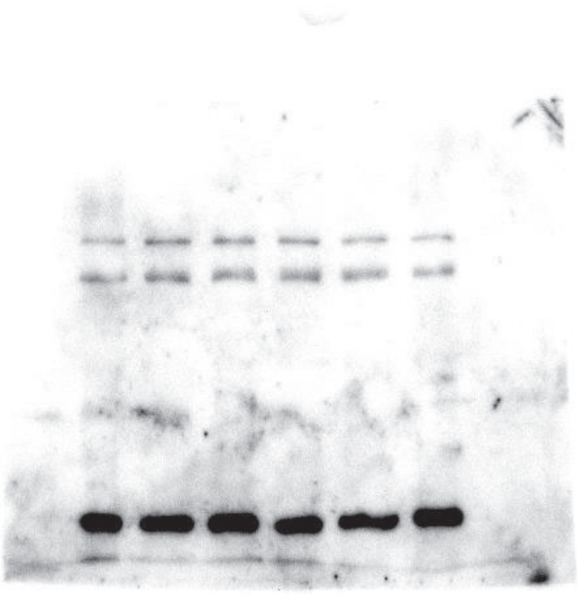

F5C NSC34 MYC

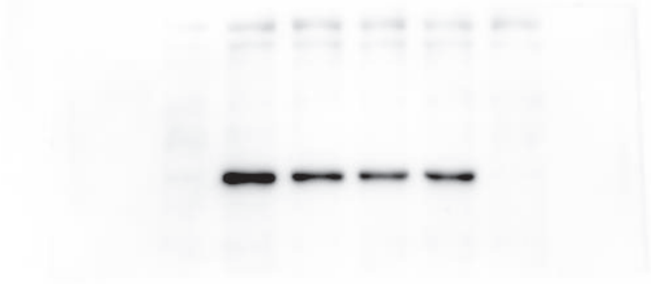

F5C NSC34 TDP43

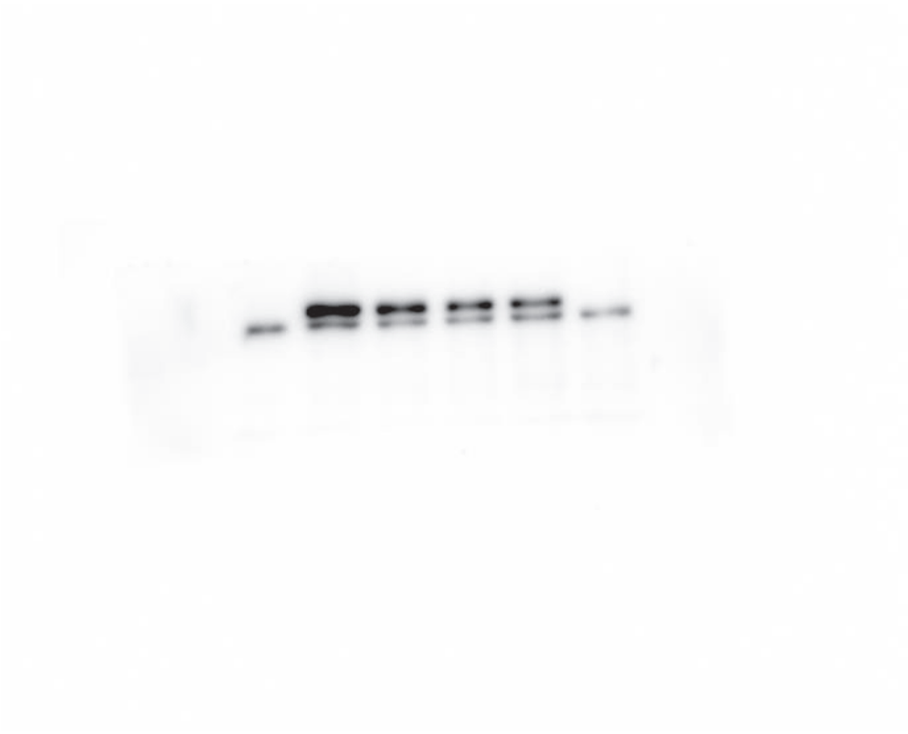

Supplementary figure 5C AD mice primary neuron cell APP

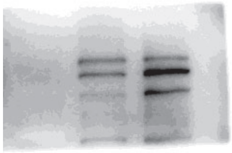

Supplementary figure 5C AD mice primary neuron cell GAPDH

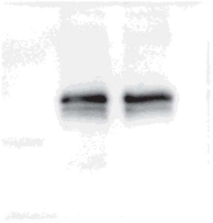

Supplementary figure 5C AD mice primary neuron cell TDP43

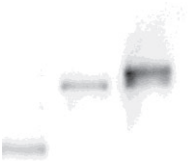

Supplementary figure 5C AD mice primary neuron cell MYC

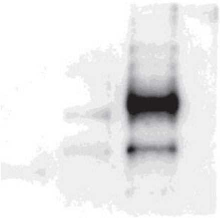

Supplement: Supplementary file 1 — supplemental data. [file 41420_2025_2340_MOESM1_ESM.pdf]
